# Highly Crosslinked Polybenzoxazines from Monobenzoxazines: The Effect of Meta-Substitution in the Phenol Ring

**DOI:** 10.3390/polym12020254

**Published:** 2020-01-21

**Authors:** Alba Martos, Marc Soto, Hannes Schäfer, Katharina Koschek, Jordi Marquet, Rosa M. Sebastián

**Affiliations:** 1 Department of Chemistry, Universitat Autònoma de Barcelona and Centro de Innovación en Química (ORFEO-CINQA), Cerdanyola del Vallés, 08193 Barcelona, Spain; alba.martos@uab.cat; 2 Fraunhofer Institute for Manufacturing Technology and Advanced Materials IFAM, Adhesive Bonding Technology and Surfaces, Wiener Strasse 12, 28359 Bremen, Germany; masohe@gmail.com (M.S.); hannes.schaefer@ifam.fraunhofer.de (H.S.); katharina.koschek@ifam.fraunhofer.de (K.K.)

**Keywords:** monobenzoxazines, para and meta phenols, thermosets, crosslinking, thermal stability, gel content, phenolic catalyst, differential scanning calorimetry, dynamic mechanical analysis

## Abstract

It is possible to control the crosslink density of polymers derived from monobenzoxazines by switching the type of substituents in the phenolic ring and their relative position with respect to the phenol group. We prepared several substituted monobenzoxazines in the para and meta positions of the phenolic ring and studied how these substituents affected the polymerization temperature of monomers and the thermal stability of the final polymers and, more extensively, how they affected the crosslink network of the final polymers. Gel content and dynamic mechanical analysis confirm that ortho- and para-orienting substituents in the meta position generate highly crosslinked materials compared to para ones. This fact can lead to the design of materials with highly crosslinked networks based on monobenzoxazines, simpler and more versatile monomers than the commercial bisbenzoxazines currently in use.

## 1. Introduction

Phenolic resins are currently highly used due to their low cost and their wide number of applications as in molding (electrical and mechanical fittings), resins (adhesive or binder for chipboard and plywood, and chemically resistant coatings for metals), laminates (general industrial use), and foams (thermal insulation). However, these resins present some limitations, such as the use of strong acids (for novolacs) or strong bases (for resoles) as catalysts, the release of water during curing, their brittleness, and their limited shelf life [1].

Polybenzoxazines show similar properties to previously described phenolic resins, such as excellent chemical and heat resistance, low dielectric constant, flame retardance, and good mechanical properties. Moreover, they present some significant advantages, namely near-zero shrinkage, no release of by-products when prepared, low water absorption, and good dimensional stability. These properties account for their increasing use in the aerospace and microelectronics industries, proton-exchange membrane fuel cells, self-lubricating and friction materials, corrosion protection, and fiber reinforcement, among others [2,3].

These thermosetting materials are obtained by high-temperature thermal polymerization of benzoxazines (T >200 °C). This constitutes one of the main drawbacks for their practical applications due to the high energetic cost required and the evaporation of the starting monomers. The use of catalysts to accelerate the polymerization reaction has been demonstrated to be a good alternative [3,4]. Benzoxazines can be easily prepared by Mannich condensations of phenols, formaldehyde, and amines, most of them being commercially available reactants and some even coming from biomass. This simple and versatile synthetic method allows a great molecular design flexibility of the final polymers, thus increasing their potential applications [2,3].

**Scheme 1:**
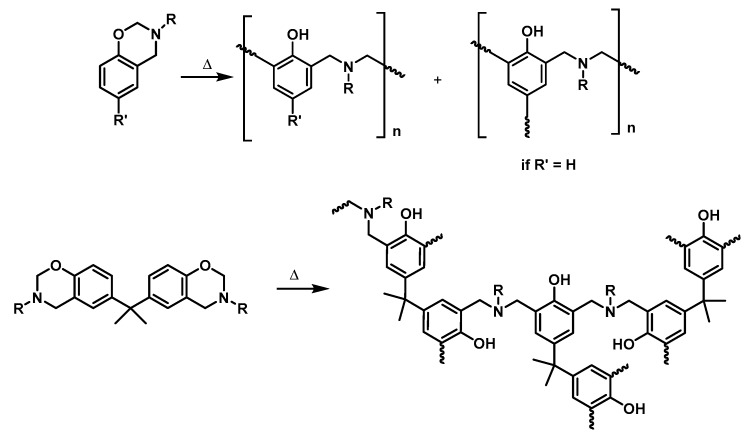
Polymerization of mono- and bisbenxozazines.

In the monobenzoxazines’ ring-opening polymerization, a covalent crosslinked network is formed in the presence of mainly Mannich methylene bridge structures (Scheme 1). Nonetheless, the degree of polymerization and the crosslinking density of these polymers are low, most probably because of the formation of intra- and intermolecular hydrogen-bonding in the polymer chainsthat deactivates the phenolic ring for further reactions [5,6]. In order to increase the crosslink density in polybenzoxazines, the use of diphenols or diamines (bisbenzoxazines) is currently common [3,7] (Scheme 1). Additional strategies have been developed to achieve this goal, such as the incorporation into monomers of additional functional groups (such as imides) [8,9], other polymerizable groups [10,11,12], and inorganic moieties (hybrid monomers) [13,14]. The copolymerization with other resins of high crosslink density [15] or the incorporation of additives increasing external hydrogen bonds [16] have also been studied.

In general, after thermal curing, monobenzoxazines give weakly crosslinked small oligomers with molecular weights usually below 2000 Da. For that reason, they are not used for the preparation of structural materials, in spite of their simpler synthesis, purification, and higher versatility compared to commercial bisbenzoxazines. The crosslinking reaction in the polymerization of monobenzoxazines proceeds through the electrophilic attack of the ring-opened benzoxazine structure with the available ortho (more activated, preferred site) and para positions of the phenolic ring of neighboring monomers (Scheme 1) [2]. The reactivity of the meta position is not so favored but has also been described at high temperatures and/or very long polymerization times [17]. If aniline derivatives are used, or other aromatic moieties are incorporated into the monomers, they also participate in the reaction increasing the crosslinking [18], an effect also previously studied by Soto et al. [19]. From the other perspective, Martos et al. have also demonstrated that the presence of different functional groups in the phenolic ring at different positions has in some particular cases a very significant effect on the kinetics of the polymerization process [20]. Following a related reasoning, we hypothesized that monobenzoxazines containing substituents in the meta positions could enhance the activity not only of the ortho position of the phenolic ring, but also of the para one during the curing process, thus increasing the crosslinking density.

In this work, we studied how the presence of three different substituents in the meta and para positions of the phenolic ring, always keeping the most active ortho position available, affected the thermal stability, the gel content, and the crosslink density of final materials. To the best of our knowledge, no studies about the influence of substituents in the meta positions of monobenzoxazines on the properties of the final materials have been reported.

## 2. Materials and Methods

### 2.1. Materials

The commercially available reagents used in the present study were purchased from Sigma Aldrich (Madrid, Spain), Alfa Aesar (Barcelona, Spain), and Fluorochem (London, UK). All reagents were used as received. 1,3,5-Triphenylhexahydro-1,3,5-triazine was synthesized by us following a previously reported methodology [21].

### 2.2. Methods of Monomer and Polymer Characterization

^1^H, ^19^F and ^13^C nuclear magnetic resonance (NMR) (Bruker, Madrid, Spain) methods were used to characterize benzoxazines. The spectra were performed in chloroform-d (CDCl_3_), using tetramethylsilane as an internal standard. The instruments used were a Bruker Advance DPX250 spectrometer (250 MHz ^1^H NMR; 235.2 MHz ^19^F NMR, 62.9 MHz ^13^C NMR), or a Bruker Advance DPX360 spectrometer (360 MHz ^1^H NMR; 90.5 MHz ^13^C NMR), or a Bruker Advance III AV400 spectrometer (400 MHz ^1^H NMR; 376.3 MHz ^19^F NMR; 100.6 MHz ^13^C NMR).

IR spectra were obtained, after averaging 16 scans in the interval from 4000 to 600 cm^−1^ (4 cm^−1^ of resolution), in a Bruker Tensor 27 FTIR spectrometer (Bruker, Madrid, Spain) equipped with a MKII Golden Gate accessory, Specac, with a diamond crystal as ATR element at a nominal incidence angle of 45° with a ZnSe lens.

The identification of some new benzoxazines was done using a high-resolution Bruker MicroTOF-Q with electrospray ionization (HRMS-ESI) (Bruker, Madrid, Spain).

Differential scanning calorimetry (DSC) tests were carried out with a TA Instrument Q20 (TA Instrument, Barcelona, Spain) using Tzero^TM^, from 0 to 300 °C, using a temperature heating rate of 10 °C/min, with standard airtight lids and pans. Experiments were performed under N_2_ flow. The instrument was previously calibrated with indium (*T*_m_ = 429.75 K, Δ*H*_m_ = 3267 kJ/mol). Samples from 2 to 5 mg were analyzed.

Dynamic mechanical analysis (DMA) was performed on a 2980 Dynamic Mechanical Analyzer from TA Instruments (Barcelona, Spain). The samples were measured using a heating rate of 2 K·min^−1^, an oscillation frequency of 1 Hz, and a strain below 0.1%.

Thermogravimetric analysis was performed on a PerkinElmer TGA 8000^TM^ instrument (Perkinelmer España S.L., Barcelona, Spain) from room temperature up to 800 °C under nitrogen atmosphere and a heating rate of 10 °C/min.

#### 2.2.1. General Procedure for the Preparation of Benzoxazine Monomers

First, 3.2 mmol of 1,3,5-triphenylhexahydro-1,3,5-triazine, 0.01 mol of corresponding phenol, and 0.01 mol of paraformaldehyde were introduced into a round-bottom flask. The mixture was then heated under magnetic stirring (for benzoxazines **2** and **7**, **8** dioxane was added as solvent). Once the reaction was finished, followed by ^1^H NMR, the crude mixture was allowed to cool at room temperature. Then, the residue was solubilized in diethyl ether, and the organic phase was washed first with 2 M NaOH aqueous solution (3 times), and then with water (3 times). After drying the organic phase with Na_2_SO_4 anh._, the solvent was evaporated, and the residue was purified by flash chromatography or recrystallized (see Supplementary Materials Figures S1, S2, S6–S12, S16, S17, S21, S22, S26, S27, S31–S33, S37–S39, S43–S45). Products obtained were compared with those previously described in the literature (**1** and **3** [22], **2** [23], **4**, **5**, **6**, **8** [20], and **7** [24]).

#### 2.2.2. General Procedure for the Polymerization of Benzoxazines

Benzoxazine monomers were melted and degassed under vacuum for 90 min. The curing program included several heating steps: 1 h heating from 60 to 180 °C, 2 h holding at 180 °C, 20 min heating from 180 to 200 °C, and 2 h holding at 200 °C. This heating profile was selected in order to achieve complete polymerization monomers according to the Huntsman Corporation [25]. Polymerizations were performed in rectangular silicon molds of 3.5 × 1.0 × 0.3 cm. The resulting polymers were quite brittle, translucent brown-orange materials.

## 3. Results

**Scheme 2:**
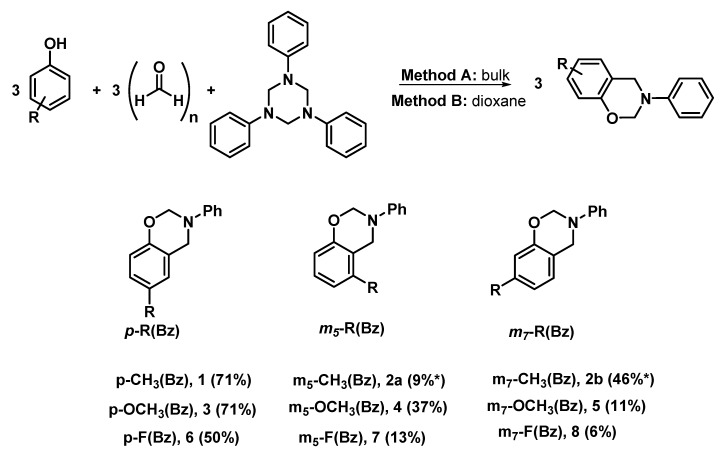
Synthesized monobenzoxazines (isolated yields). Yields marked with * were determined by ^1^H NMR.

To prepare new materials showing interesting properties, the most common and simple strategy is the incorporation of functional moieties into the phenolic ring of the benzoxazine monomers. This route is quite simple due to the availability of a large amount of different commercial phenols that can also be easily modified. The main aim of this work was to establish how the introduction of substituents into the phenolic ring of monobenzoxazines affects the crosslink density of the final polymeric material. A high degree of crosslinking is generally reflected in higher thermal stability, char yield, and better mechanical properties. We decided to maintain the ortho position free in all cases, because it is the most active position for the electrophilic attack by the oxazine ring or the iminium cation during the reaction, thus ensuring that the basic structure of the polybenzoxazines is kept. Three families of para- and meta-phenolic-substituted benzoxazines containing CH_3_, MeO, and F groups (ortho- and para-orienting groups) that could promote crosslinking were prepared. The synthetic strategy used for the preparation of those monofunctional benzoxazine monomers was described in the literature by Ronda et al. (Scheme 2) [24]. We performed the Mannich condensation of meta- and para-substituted phenols with paraformaldehyde and 1,3,5-triphenylhexahydro-1,3,5-triazine in the molar ratio 3:3:1 without solvent. This simple method allowed the preparation of compounds **1**, **3**, **4**, **5**, and **6** (Method A, Scheme 2). Yields obtained were comparable to the ones previously reported [20,22]. When uncontrolled polymerization occurred during the synthesis of monomers, the use of 1,4-dioxane as solvent under reflux conditions allowed us to minimize this process (Method B, Scheme 2), making it possible to obtain compounds **2a/b**, **6**, and **7** [20,23,24]. In this case, the reactions were performed at lower reaction temperatures compared to the bulk methodology. Nevertheless, lower yields were obtained after longer reaction times, due mainly to the formation of oligomers. Column chromatography was used to purify most of the compounds, except **1** and **6** that were recrystallized.

We used the nomenclature *p*-R(Bz), *m*_5_-R(Bz), and *m*_7_-R(Bz), where *p* (para), *m*_5_ (meta), and *m*_7_ (meta) referred to the positions of the R substituent in the phenolic ring of the monobenzoxazine monomer. When *m*-substituted phenols were used as reactants, two isomeric monobenzoxazines were obtained. Both isomers derived from the methoxy and fluorine groups were isolated by column chromatography; however, this process was difficult and yields were quite low. Separation was not possible for the isomers derived from *m*-cresol; however, the yield of each isomer is indicated in Scheme 2, determined by ^1^H NMR integration of the pure mixture. To identify each meta isomer, 2D NMR (COSY, HSQC, HMBC) experiments were performed, allowing us to determine the ratio of the isomers as 17:83, **2a**:**2b** (*m*_5_-CH_3_(Bz):*m*_7_-CH_3_(Bz) (see Supplementary Materials Figures S6–S10).

### 3.1. Differential Scanning Calorimetry (DSC) Analysis

**Figure 1:**
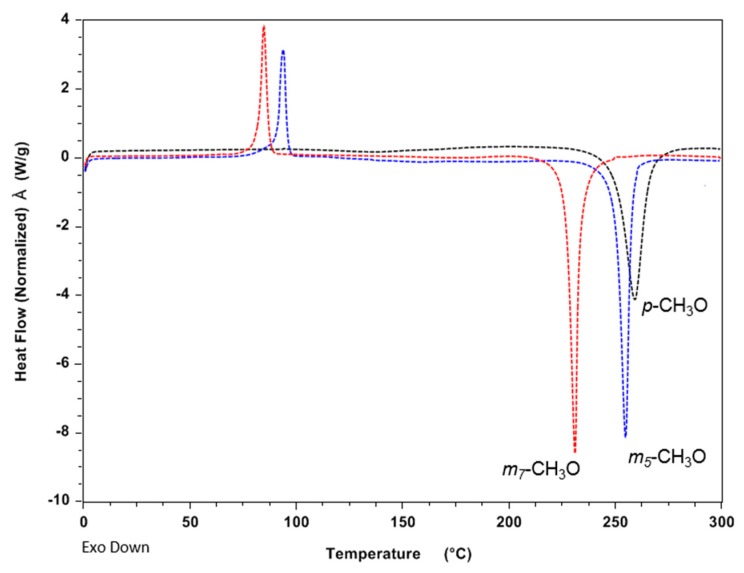
DSC thermogram of benzoxazines **3**, **4**, and **5** at heating rate 10 °C/min from 0 to 300 °C.

The polymerization temperature of the synthesized monofunctional benzoxazines was determined by DSC (heating rate of 10 °C/min under nitrogen atmosphere). The thermograms showed a typical profile, in which a highly exothermic peak corresponds to the thermal ring-opening polymerization (ROP) of monomers. The melting point of the solid monomers was also identified. No degradation process was observed by this technique up to 300 °C. Table 1 summarizes all data obtained from the DSC experiments of all studied compounds. The DSC thermograms of –OCH_3_(Bz) isomers are shown in Figure 1.

The polymerization process is clearly influenced by the electronic effects generated by the substituents anchored at different positions of the phenolic ring of the monomers [20,24]. These substituents can favor or not the rate-limiting step of the ring-opening process. The fluorine atom anchored in the *p*-position shows an almost neutral electronic effect on the phenoxy group (Hammett constant, σ*_para_* 0.062), which reflects the highest polymerization temperature observed for compound **6** (T_p(peak)_ = 276 °C). A similar value has been reported for non-substituted benzoxazines (272 °C [3]). However, methyl and methoxy groups, showing a larger electron-donor effect (σ*_para_* −0.170 and −0.268, respectively), favor the proton-catalyzed ring-opening process [20], and T_p(peak)_ is reduced by more than 10 °C (compare compounds **1** and **3** with **6**, Table 1). The electronic effects are completely different if the substituents are located in meta positions. In this case, methyl has nearly no effect on the ring-opening process (σ*_meta_* −0.069), whereas the methoxy and fluorine groups yield a significant reduction of the polymerization temperature (σ*_meta_* 0.115 and 0.337), up to 45 °C for *m*_5_-F(Bz) (compare **3** with **4** and **5,** and **6** with **7** and **8**, respectively, Table 1). Taking into account the acidity of *m*-MeO-PhOH and *m*-F-PhOH (pKa ~9.65 and 9.28, respectively), an auto-catalysis is probably responsible for this significant reduction of T_p_ [19]. Moreover, the most active positions in the phenolic ring are free and contribute to the observed polymerization temperature reduction. Differences observed between *m*_5_ and *m*_7_ positions were discussed in a previous work; however, no clear general explanation was achieved for different functional groups [20].

These electronic effects should affect not only the rate of the polymerization process, but also the reactivity of the free positions of the aromatic ring that can participate in the crosslinking process through electrophilic aromatic substitution reactions. This has a potential effect on the crosslinking density of the final materials, as we analyze below. The first signs of this behavior appeared when analyzing the energy released in the polymerization reactions. The enthalpy of the polymerization process related to monofluorinated benzoxazines **7** and **8** is 17 to 26 KJ/mol higher than the average of other studied monomers (~72.9 KJ/mol). This fact makes us suspect that extra curing reactions exist, compared to the other substituents, increasing the crosslink density of the final materials. The fluorine atom should activate the ortho and para positions of the phenolic ring (very small ortho- and para-orienting substituent) for potential electrophilic aromatic substitution. In fact, aromatic fluorine substituents in the side chains (aniline component) of bisbenzoxazines have been very recently used to increase the crosslinking density in their derived polybenzoxazine materials [26].

### 3.2. Polymerization of Monobenzoxazines

All synthesized monobenzoxazines depicted in Scheme 2 were subjected to thermal treatment under vacuum for 5 h using the curing program described in the experimental section. In all cases, complete polymerization was observed by ^1^H NMR spectroscopy of the soluble part of the final materials, which showed the disappearance of the methylene groups’ signals of the monobenzoxazines’ oxazine ring around 4.6 and 5.3 ppm (see Supplementary Materials Figures S58–S65). Moreover, IR spectra of the final materials were performed and the disappearance of the intense absorption band in the region of 920–950 cm^−1^ related to the skeletal vibration mode of cyclic oxazine also confirmed the ring opening of all monomers [2,3] (see Supplementary Materials Figures S49–S57). The obtained materials were subjected to thermogravimetric analysis (TGA), gel content determination, and dynamic mechanical analysis (DMA), and the respective results are described and discussed in the following sections.

### 3.3. Thermogravimetric Analysis (TGA)

The internal structure of polymers can play an important role in their thermal stability. This property is one of the key points in many applications. The results of the thermogravimetric analysis of the polybenzoxazine materials prepared from the substituted monobenzoxazines indicated in Scheme 2 are summarized in Table 2. The analyses were performed from room temperature up to 800 °C under nitrogen atmosphere, and temperatures at 5% and 10% weight loss (T_5%_ and T_10%_, respectively) and char yield were determined. C–H, C–C, and C–N bonds of aliphatic polymers generally become unstable at temperatures above 300 °C even in a nitrogen or vacuum environment; however, when aromatic rings and internal hydrogen bonds are present in the chemical structures of polymers, the stability increases [27].

**Scheme 3:**
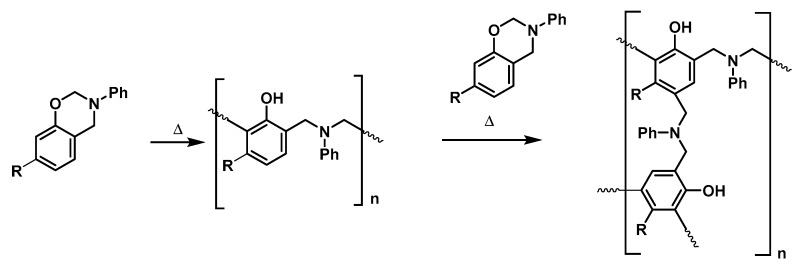
Proposed crosslinked structures for *m*-substituted polybenzoxazines derived from monobenzoxazines considering only reactions on the phenolic ring.

No clear effects were observed in the thermal stability data when substituents were introduced into the para or meta positions of the phenolic ring, except for the case of the methyl derivative where the temperature at 5% weight loss was 67 °C higher for the meta isomer (*m*-CH_3_ T_5%_ = 328 versus T_5%_ = 261 for *p*-CH_3_). In all cases, a better stability of substituted polymers was observed when compared to the previously reported polybenzoxazine prepared from the corresponding unsubstituted monobenzoxazine (T_5%_ = 237 °C and T_10%_ = 281 °C, [19]). Interestingly enough, fluorine-substituted polybenzoxazines showed a significantly higher stability regardless of the position of the substituent [27]. Moreover, in the cases of methoxy and fluorine substituents, the meta isomers showed a significant and consistent higher char yield value than their para isomers. For three of the meta-substituted derived polymers, higher values than the previously reported ones for the corresponding unsubstituted polybenzoxazine material (39.8%) [28] were obtained, again making the results remarkable for the fluorinated materials. A plausible explanation for these results is that the final meta-substituted polymers probably have a higher crosslink density due to the fact that they can react at both the ortho and the para positions during curing. Electronic effects (ortho- and para-orienting) and low steric hindrance of the substituents (especially in the case of fluorine) promote electrophilic aromatic substitution reaction, responsible for the crosslinking (Scheme 3). We tried to support this hypothesis by studying gel content and dynamic mechanical analysis of the polymeric samples.

Finally, the polymers’ flame retardancy was calculated from the limiting oxygen index (LOI), defined as the minimum fraction of O_2_ in a mixture of O_2_ and N_2_ that just supports flaming combustion [29]. Char yield of a material can be used to estimate LOI according to the Van Krevelen and Hoftyzer equation (Equation (1)) [29,30]:

(1) LOI=17.5+0.4 (CY).

The LOI values of the polymers must be above the threshold value of 21 to render them self-extinguishing and to ensure their adequacy for applications requiring good flame resistance. In our case, all the monobenzoxazines exhibited good flame-retardant properties; values from 30.5 to 35.9 were obtained (Table 2).

### 3.4. Study of Gel Content

To obtain first evidence of the network formed after polymerization, the gel content of polybenzoxazines was quantified by 24 h Soxhlet extraction using acetone as solvent. Linear polymer chains were solved and removed, leaving behind the insoluble 3D network. Thus, gel content was calculated by the division of the final gel network weight after extraction (*m_network_*) by the initial polybenzoxazine weight (*m*_0_) (Equation (2)):

(2) $$Gel\;content\;\%=\frac{m_{network}}{m_{0}}\times 100.$$

**Figure 2:**
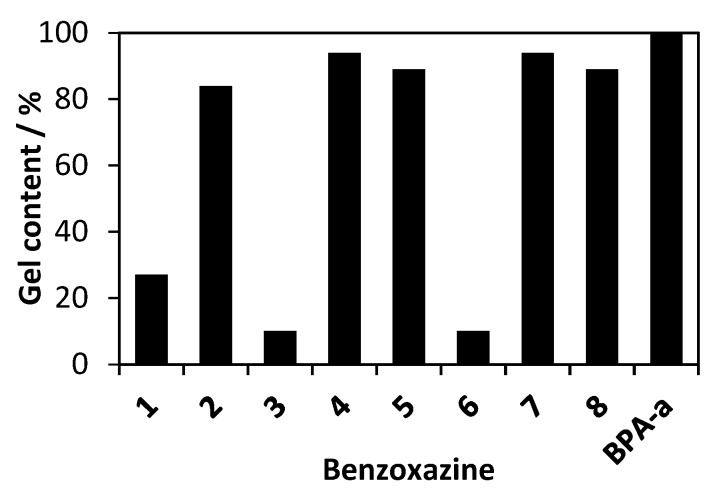
Gel content of polybenzoxazines derived from monobenzoxazines determined by acetone extraction. BPA-a corresponds to the polybenzoxazine prepared from the bisbenzoxazine, previously synthesized by us, derived from bisphenol A and aniline under our conditions.

The experimental results of the studied polybenzoxazines are shown in Figure 2. Benzoxazines **1**, **3**, and **6** produced materials with a low gel content (27%, 10%, and 10%, respectively), whereas materials from benzoxazines **2**, **4**, **5**, **7**, and **8** provided materials with gel contents higher than 84%.

These results correlate with the structure of the corresponding benzoxazines and confirm the trends observed in the results described in Table 2. Even though branching and crosslinking can take place through the aniline ring [19], these reactions seems to be less effective than the ones produced at the phenolic ring. Thus, on the one hand, polybenzoxazines prepared from **1**, **3**, and **6**, which have the para-phenolic positions blocked, were mainly formed by linear oligomers, so fewer crosslinked networks were obtained and they showed a low gel content. On the other hand, benzoxazines **2**, **4**, **5**, **7**, and **8**, containing substituents in the meta positions, promoted much higher crosslinked materials, as suggested by their high gel content. In these cases, both active positions can react and this reaction seems to be enhanced by the meta ortho- and para-orienting substituents (guiding the reactivity to their two free ortho relative positions). Gel contents of these polymers coming from monobenzoxazines are quite similar to the ones that we obtained and following the same polymerization procedure (see the experimental section) for the polybenzoxazine obtained from the bisbenzoxazine derived from bisphenol A and aniline (Figure 2). Steric hindrance of substituents seems to cause a small effect.

While gel content provided information about the relative amount of bounded chains to the network, it did not give information about the extent of the crosslinking, how dense and tight was this network. To obtain further information about the network and the crosslink density, a more accurate analysis was required.

### 3.5. Dynamic Mechanical Analysis (DMA) and Crosslink Density of Polymers

Crosslink density *v*_e_ was defined by Flory as the number of elastically effective network chains per unit of volume. This takes into account polymer chains between two crosslinking points, while leaving out terminal linear polymer chains [31]. In thermosets with a high crosslinking degree, *v*_e_ can be experimentally determined from the rubbery plateau modulus *E*’(*T*_r_), that is, the modulus above the glass transition range, using DMA. To achieve meaningful values, it is crucial to use small sample deformations, so that the deformation only induces conformational changes in the network rather than bending or breaking bonds. Based on the storage moduli determined by DMA measurements, the crosslinking density *v*_e_ was calculated as an approximation from the following equation (Equation (3)) [32]:

(3) ve=E′(Tr)3RTr,

where, in SI units, *v*_e_ is the crosslink density or number of elastically effective network chains per volume, in mol m^−3^; *E*’(*T*_r_) is the storage modulus in the rubbery plateau, modulus above *T*_g_, expressed in Pa; *R* is the universal gas constant, 8.314 J·K^−1^·mol^−1^; and *T*_r_ is the temperature above the glass transition range of the given storage modulus, in K.

As polybenzoxazines are highly crosslinked polymers, their crosslink density can be and has been calculated applying the above-mentioned equation [33]. Glass transition temperatures, rubbery plateau, elastic modulus at 25 °C and at *T*_r_, and calculated crosslink densities of our polybenzoxazines are summarized in Table 3.

For the three studied substituents (Me, OCH_3_, F), their position in the phenolic ring has a strong effect on the *T*_g_ of the final materials. When they occupy a para position, *T*_g_ is from 17 to 35 °C lower than when they are located in the meta positions (Table 3). A similar effect was observed when crosslink density was determined according to Equation (3). In all cases, polymers derived from para-substituted phenolic ring benzoxazines showed lower crosslink densities. For methyl and fluorine substituents, the differences observed were very large, namely 1050 and ~930 mol·m^−3^, respectively, being higher in the meta cases when compared to the para ones. The differences observed between polymers containing methoxy groups in para or meta positions of the phenolic ring were significant but more moderate, namely 242 (*m*_7_-OCH_3_(Bz) versus *p*-OCH_3_(Bz)) and 502 mol·m^−3^ (*m*_5_-OCH_3_(Bz) versus *p*-OCH_3_(Bz)). These results confirm the important role of the free para position of the phenolic ring in the crosslinking processes. As expected, the higher the *T*_g_ of polymers, the higher the crosslink density (Table 3).

It is worth highlighting that the polymers obtained from monofunctional benzoxazines **7** and **8** containing fluorine atoms in the meta position of the phenolic ring exhibited very high crosslinking densities (1652 and 1681 mol·m^−3^ for *m*_5_ and *m*_7_, respectively). These values are approximately two-thirds of the crosslinking density of the polymer derived from commercial bifunctional benzoxazine BPA-a obtained by us under our reaction conditions (2490 mol·m^−3^), in which every monomer is a branching point itself, but it has the para position of the phenolic ring blocked. The high conjugative electron-donor effect of the fluorine atom to the aromatic ring activates its neighboring positions, promoting the polymerization reaction at lower temperatures compared to the other studied substituents and the crosslinking processes. Moreover, the nearly zero steric effects generated by the presence of the fluorine atom, compared to the ones generated by the methoxy and methyl groups, have a clear effect on the final polymeric network. In these last cases, crosslink densities were significantly lower (around 1100 mol·m^−3^).

**Figure 3:**
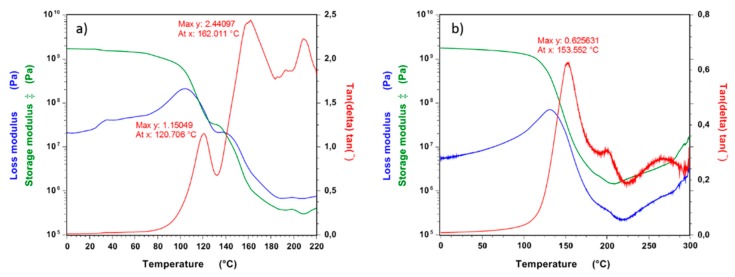
DMA of polymer derived from benzoxazine (**a**) **1** and (**b**) **6**. Storage (green) and loss (blue) moduli and tan(delta) tan (red) are represented.

In contrast to the monofunctional benzoxazines **7** and **8** containing fluorine atoms in the meta position, the polybenzoxazine containing a methyl group in the para position of the phenolic ring derived from benzoxazine **1** exhibited an extremely low crosslink density (36 mol·m^−3^), indicating that the material comprised mainly linear chains lacking a covalent network. This assumption was confirmed by a loss modulus that was higher than the storage modulus at elevated temperatures, which is typical for liquids (Figure 3a) [34]. In all other polymers studied (meta- or para-substituted), the loss modulus showed lower values than the storage modulus, pointing to a solid-like behavior in the studied temperature range (see, as an example, the polymer derived from **6** in Figure 3b).

These experiments confirmed that monobenzoxazines containing ortho- and para-orienting groups in their structure in the meta positions of the phenolic ring show highly crosslinked networks after polymerization compared to para-substituted ones. A remarkable increase is observed when the substituent is the fluorine atom (very low steric hindrance).

## 4. Conclusions

Currently, the general approach to obtaining highly crosslinked polybenzoxazines is the use of bifunctional monomers. However, monobenzoxazines are simpler to prepare and are more versatile, having the ability to introduce substituents into their structures, substituents that can provide special properties to final materials. In this sense, it seems appropriate to study the effect of substituents and their positions in the phenolic ring on the final properties of the derived polymeric materials. With this aim, we prepared eight monobenzoxazines containing one ortho- and para-orienting substituent in the phenolic ring and established how the nature (CH_3_, OCH_3_, F) and the position of this substituent (meta or para) affected the final properties of the final polymers, paying special attention to their effect on the crosslink density. First, it was observed that the presence of substituents in the meta position reduced the *T*_p_; when the substituent was fluorine, the reduction was more than 40 °C, in agreement with related studies described by some of us [20]. Moreover, we observed that when monobenzoxazines were substituted in meta positions, the corresponding polymers presented higher char yields, their gel content increased up to 84%, and *T*_g_ increased at the same time as the crosslink density. When the methyl group was the substituent, steric effects reduced the crosslink density. We would like to highlight the remarkable effect of the presence of a fluorine atom in the meta position of the monobenzoxazine (ortho- or para-orienting and small size), that not only facilitated the polymerization reaction at lower temperatures as commented before, but also activated the aromatic ring in such a way that crosslinking processes were promoted. This type of benzoxazine could be incorporated into other benzoxazine mixtures (as doping material) to increase the final crosslinking network.

## Figures and Tables

**Table 1:** Differential scanning calorimetry (DSC) analysis of *p*- and *m*-substituted monobenzoxazines.

Benzoxazine	DSC Thermogram			
	*T*_m(onset)_ (°C) ^a^	*T*_p(onset)_ (°C) ^b^	*T*_p(peak)_ (°C) ^c^	∆*H* (kJ·mol^−1^) ^d^
*p*-CH_3_(Bz), **1**	47.5	257	265	−72.8
*m*-CH_3_ (Bz), **2 ^e^**	oil	250	257	−76.4
*p*-OCH_3_(Bz), **3**	oil	250	259	−77.6
*m*_5_-OCH_3_(Bz), **4**	91.2	250	255	−68.5
*m*_7_-OCH_3_(Bz), **5**	82.5	227	231	−69.5
*p*-F(Bz), **6**	48.2	271	276	−72.7
*m*_5_-F(Bz), **7**	oil	226	231	−90.0
*m*_7_-F(Bz), **8**	oil	231	237	−99.6

^a^ Melting temperature. ^b^ Onset of polymerization of exotherm. ^c^ Peak maximum of exotherm. ^d^ Polymerization heat. ^e^ Mixture of isomers, ratio 17:83 (*m*_5_-CH_3_(Bz):*m*_7_-CH_3_(Bz). See thermograms in Supplementary Materials Figures S3, S13, S18, S23, S28, S34, S40, S46.

**Table 2:** Thermogravimetric analysis (TGA) of polybenzoxazines obtained from thermal treatment of substituted monobenzoxazines.

Polybenzoxazine	T_5%_^a^ (°C)	T_10%_^b^ (°C)	CY ^c^ (%)	LOI ^d^
*p*-CH_3_	261	298	32.9	30.7
*m*-CH_3_	328	362	32.4	30.5
*p*-OCH_3_	317	354	37.4	32.5
*m*_5_-OCH_3_	322	352	41.4	34.1
*m*_7_-OCH_3_	306	334	38.3	32.8
*p*-F	340	372	41.5	34.1
*m*_5_-F	338	374	45.0	35.5
*m*_7_-F	353	383	45.9	35.9

^a^ Temperature at 5% weight loss; ^b^ Temperature at 10% weight loss; ^c^ Char yield at 800 °C under nitrogen; ^d^ Limiting oxygen index value. See thermograms in Supplementary Materials Figures S4, S14, S19, S24, S29, S35, S41, S47.

**Table 3:** Dynamic mechanical analysis (DMA) results and calculated crosslink densities.

Polybenzoxazine	*E*’(25 °C) ^c^ /GPa	*T*_g_^a^ /°C	*T*_r_^b^ /°C	*E*’(*T*_r_) ^c^ /MPa	*v*_e_^d^ /mol·m^−3^
*p*-CH_3_(Bz)	1.67	120	185	0.41	36
*m*-CH_3_ (Bz) ^e^	3.11	155	210	13.1	1090
*p*-OCH_3_(Bz)	3.41	135	210	8.09	671
*m*_5_-OCH_3_(Bz)	2.79	158	210	14.1	1173
*m*_7_-OCH_3_(Bz)	2.76	153	210	11.4	943
*p*-F(Bz)	2.48	154	210	8.67	720
*m*_5_-F(Bz)	3.15	171	240	21.1	1652
*m*_7_-F(Bz)	2.87	172	220	20.7	1681
*BPA-a*	3.00	150	210	30.0	2490

^a^*T*_g_ measured as tan(delta) peak in DMA. ^b^ *T*_r_ Temperature of the rubbery plateau. ^c^ Storage modulus in the rubbery plateau. ^d^ Crosslink density. ^e^ Mixture of meta isomers. See thermograms in Supplementary Materials Figures S5, S15, S20, S25, S30, S36, S42, S48.

## Supplementary Materials

The following are available online at https://www.mdpi.com/2073-4360/12/2/254/s1. Contents: Experimental details, NMR, IR, HRMS, DSC, TGA, and DMA experiments of compounds 1, 2a and 2b, 3, 4, 5, 6, 7, and 8. Figure S1: ^1^H NMR (250 MHz) spectrum of 1 in CDCl_3_; Figure S2: IR (ATR) υ (cm^−1^) of 1; Figure S3: DSC thermogram of 1; Figure S4: TGA thermogram of 1; Figure S5: DMA thermogram of 1; Figure S6: ^1^H NMR (360 MHz) spectrum of 2a and 2b in CDCl_3_; Figure S7: ^13^C NMR (90.5 MHz) spectrum of 2a and 2b in CDCl_3_; Figure S8: ^1^H-^1^H COSY NMR (360 MHz) spectrum of 2a and 2b in CDCl_3_; Figure S9: HSQC NMR (360/90.5 MHz) spectrum of 2a and 2b in CDCl_3_; Figure S10: HMBC NMR (360/90.5) spectrum of 2a and 2b in CDCl_3_; Figure S11: IR (ATR) υ (cm^−1^) of 2a and 2b; Figure S12: HRMS (ESI/Q-TOF) [M+H^+^] of 2a and 2b; Figure S13: DSC thermogram of 2a and 2b; Figure S14: TGA thermogram of 2a and 2b; Figure S15: DMA thermogram of 2a and 2b; Figure S16: ^1^H NMR (250 MHz) spectrum of 3 in CDCl_3_; Figure S17: IR (ATR) υ (cm^−1^) of 3; Figure S18: DSC thermogram of 3; Figure S19: TGA thermogram of 3; Figure S20: DMA thermogram of 3; Figure S21: ^1^H NMR (250 MHz) spectrum of 4 in CDCl_3_; Figure S22: IR (ATR) υ (cm^−1^) of 4; Figure S23: DSC thermogram of 4; Figure S24: TGA thermogram of 4; Figure S25: DMA thermogram of 4; Figure S26: ^1^H NMR (400 MHz) spectrum of 5 in CDCl_3_; Figure S27: IR (ATR) υ (cm^−1^) of 5; Figure S28: DSC thermogram of 5; Figure S29: TGA thermogram of 5; Figure S30: DMA thermogram of 5; Figure S31: ^1^H NMR (250 MHz) spectrum of 6 in CDCl_3_; Figure S32: ^19^F NMR (235.2 MHz) spectrum of 6 in CDCl_3_; Figure S33: IR (ATR) υ (cm^−1^) of 6; Figure S34: DSC thermogram of 6; Figure S35: TGA thermogram of 6; Figure S36: DMA thermogram of 6; Figure S37: ^1^H NMR (250 MHz) of 7 in CDCl_3_; Figure S38: ^19^F NMR (235.2 MHz) of 7 in CDCl_3_; Figure S39: IR (ATR) υ (cm^−1^) of 7; Figure S40: DSC thermogram of 7; Figure S41: TGA thermogram of 7; Figure S42: DMA thermogram of 7; Figure S43: ^1^H NMR (250 MHz) spectrum of 8 in CDCl_3_; Figure S44: ^19^F NMR (235.2 MHz) spectrum of 8 in CDCl_3_; Figure S45: IR (ATR) υ (cm^−1^) of 8; Figure S46: DSC thermogram of 8; Figure S47: TGA thermogram of 8; Figure S48: DMA thermogram of 8; Figure S49: IR (ATR) ʋ (cm^−1^) of polymer derived from 1; Figure S50: IR (ATR) ʋ (cm^−1^) of polymer derived from 2; Figure S51: IR (ATR) ʋ (cm^−1^) of polymer derived from 3; Figure S52: IR (ATR) ʋ (cm^−1^) of polymer derived from 4; Figure S53: IR (ATR) ʋ (cm^−1^) of polymer derived from 5; Figure S54: IR (ATR) ʋ (cm^−1^) of polymer derived from 6; Figure S55: IR (ATR) ʋ (cm^−1^) of polymer derived from 7; Figure S56: IR (ATR) ʋ (cm^−1^) of polymer derived from 8; Figure S57: IR (ATR) ʋ (cm^−1^) of polymer derived from BPA-a; Figure S58: ^1^H NMR (250 MHz) of polymer derived from 1 in DMSO-d_6_; Figure S59: ^1^H NMR (360 MHz) of polymer derived from 2 in DMSO-d_6_; Figure S60: ^1^H NMR (360 MHz) of polymer derived from 3 in DMSO-d_6_; Figure S61: ^1^H NMR (360 MHz) of polymer derived from 4 in DMSO-d_6_; Figure S62: ^1^H NMR (360 MHz) of polymer derived from 5 in DMSO-d_6_; Figure S63: ^1^H NMR (360 MHz) of polymer derived from 6 in DMSO-d_6_; Figure S64: ^1^H NMR (360 MHz) of polymer derived from 7 in DMSO-d_6_; Figure S65: ^1^H NMR (360 MHz) of polymer derived from 8 in DMSO-d_6_.
