# Supplementary material for: Highly Crosslinked Polybenzoxazines from Monobenzoxazines: The Effect of Meta-Substitution in the Phenol Ring
Source: Polymers (Basel). 2020 Jan 21;12(2):254. doi: 10.3390/polym12020254 (PMC7077280; doi:10.3390/polym12020254)
Supplement: Supplementary file 1 [file polymers-12-00254-s001.pdf]

Supporting Information

Highly crosslinked polybenzoxazines from monobenzoxazines.The effect of *meta*-substitution in the phenol ring

Alba Martos,[1] Marc Soto,[2] Hannes Schäfer,[2] Katharina Koschek,[2] Jordi Marquet[1,*], Rosa M. Sebastián[1,*]

[1] Department of Chemistry, Universitat Autònoma de Barcelona and Centro de Innovación en Química (ORFEO-CINQA), Cerdanyola del Vallés, 08193, Barcelona, Spain; rosamaria.sebastian@uab.es

[2] Fraunhofer Institute for Manufacturing Technology and Advanced Materials IFAM, Adhesive Bonding Technology and Surfaces, Wiener Strasse 12, 28359, Bremen, Germany rosamaria.sebastian@uab.es

Contents
1D and 2D NMR spectra
IRs
HRMS
DSC thermograms
TGA thermograms
DMA thermograms

Table of contents

S1. Experimental details and descripcion of molecules......SI2

S2. 6-Methyl-3-phenyl-3,4-dihydro-2*H*-1,3-benzoxazine, **1**...... SI5

S3. 5-Methyl-3-phenyl-3,4-dihydro-2*H*-1,3-benzoxazine, **2a** and 7-Methyl-3-phenyl-3,4-dihydro-2*H*-1,3-benzoxazine, **2b**...... SI8

S4. 6-Methoxy-3-phenyl-3,4-dihydro-2*H*-1,3-benzoxazine, **3**...... SI13

S5. 5-Methoxy-3-phenyl-3,4-dihydro-2*H*-1,3-benzoxazine, **4**...... SI16

S6. 7-Methoxy-3-phenyl-3,4-dihydro-2*H*-1,3-benzoxazine, **5**...... SI19

S7. 6-Fluoro-3-phenyl-3,4-dihydro-2*H*-1,3-benzoxazine, **6**...... SI22

S8. 5-Fluoro-3-phenyl-3,4-dihydro-2*H*-1,3-benzoxazine, **7**...... SI25

S9. 7-Fluoro-3-phenyl-3,4-dihydro-2*H*-1,3-benzoxazine, **8**...... SI28

S10. IR spectra of polymers obtained from benzoxazines **1**-**8** and **BPA-a** SI32

S11. 1H-NMR spectra of soluble portion of polymers obtained from benzoxazines **1**-**8** SI37

S12. References, ... SI41

S1.Experimental details and spectroscopic data of benzoxazines synthesized by method A (without solvent).

6-Methyl-3-phenyl-3,4-dihydro-2H-1,3-benzoxazine, ***1***. [1] The resulting product was crystallized from hexane, obtaining a white powder (2.96 g, 71 % yield). Mp: 47.5 °C ($T_{m(onset)}$, DSC), Mp (lit.[1]): 55-56 °C. ^{1}H NMR (250 MHz, $CDCl_3$) δ(ppm): 2.25 (s, 3H, C_{Ar}-C$\underline{H_3}$), 4.60 (s, 2H, C_{Ar}-C$\underline{H_2}$-N), 5.34 (s, 2H, O-C$\underline{H_2}$-N), 6.71 (d, *J* = 10 Hz, 1H, Ar-$\underline{H}$), 6.83 (s, 1H, Ar-$\underline{H}$), 6.92 (t, *J* = 8.8 Hz, 2H, Ar-$\underline{H}$), 7.11 (d, *J* = 7.5 Hz, 2H, Ar-$\underline{H}$), 7.26 (t, *J* = 7.5 Hz, 2H, Ar-$\underline{H}$). IR (ATR) ν(cm^{-1}): 1601, 1493, 1455, 1416, 1365, 1326, 1298, 1258, 1219, 1168, 1143, 1120, 1091, 1035, 941, 915, 884, 820, 796, 745, 687.

6-Methoxy-3-phenyl-3,4-dihydro-2H-1,3-benzoxazine, ***3*** [1]. The resulting orange oil was purified by flash chromatography (silica gel, hexane: DCM = 2:1 v/v as eluent), obtaining a light orange oil (13.15 g, 71 % yield). ^{1}H NMR (250 MHz, $CDCl_3$) δ(ppm): 3.77 (s, 3H, C_{Ar}-O-C$\underline{H_3}$), 4.63 (s, 2H, C_{Ar}-C$\underline{H_2}$-N), 5.34 (s, 2H, O-C$\underline{H_2}$-N), 6.59 (s, 1H, Ar-$\underline{H}$), 6.76 (m, 2H, Ar-$\underline{H}$), 6.95 (t, *J* = 7.1 Hz, 1H, Ar-$\underline{H}$), 7.12 (d, *J* = 7.9 Hz, 2H, Ar-$\underline{H}$), 7.28 (m, 3H, Ar-$\underline{H}$). IR (ATR) ν(cm^{-1}): 1655, 1599, 1493, 1429, 1367, 1328, 1273, 1256, 1221, 1191, 1143, 1036, 947, 914, 801, 755, 694.

Methoxy-3-phenyl-3,4-dihydro-2H-1,3-benzoxazines ***4*** *and* ***5***. The resulting yellow oil was directly subjected to flash column chromatography (silica gel, hexane: EA = 9.5:0.5 v/v as eluent). Total yield for *m*-OCH_3(Bz): 48 % (1.15 g) (Product **4**: white solid 37 % yield (888 mg) and product **5**: white solid 11% yield (264 mg)).

5-Methoxy-3-phenyl-3,4-dihydro-2H-1,3-benzoxazine, ***4*** [2]. Mp: 91.2 °C ($T_{m(onset)}$, DSC). ^{1}H NMR (250 MHz, $CDCl_3$) δ(ppm): 3.81 (s, 3H, O-C$\underline{H_3}$), 4.55 (s, 2H, Ar-C$\underline{H_2}$-N), 5.32 (s, 2H, O-C$\underline{H_2}$-N), 6.42 (d, *J* = 8.3 Hz, 1H, Ar-$\underline{H}$), 6.46 (d, *J* = 8.5 Hz, 1H, Ar-$\underline{H}$), 6.91 (t, *J* = 7.3 Hz, 1H, Ar-$\underline{H}$), 7.05 (d, *J* = 8.3 Hz, 1H, Ar-$\underline{H}$), 7.11 (d, *J* = 8.8 Hz, 2H, Ar-$\underline{H}$), 7.13-7.29 (m, 2H, Ar-$\underline{H}$). IR (ATR) ν(cm^{-1}): 2901, 1589, 1497, 1470, 1456, 1435, 1375, 1268, 1237, 1032, 1105, 1070, 943, 892, 780, 771, 752, 696.

7-Methoxy-3-phenyl-3,4-dihydro-2H-1,3-benzoxazine, ***5*** [2]. Mp: 82.5 °C ($T_{m(onset)}$, DSC). ^{1}H NMR (400 MHz, $CDCl_3$) δ(ppm): 3.73 (s, 3H, O-C$\underline{H_3}$), 4.57 (s, 2H, Ar-C$\underline{H_2}$-N), 5.34 (s, 2H, O-C$\underline{H_2}$-N), 6.37 (s, 1H, Ar-$\underline{H}$), 6.48 (d, *J* = 8.4 Hz, 1H, Ar-$\underline{H}$), 6.90 (d, *J* = 8.0 Hz, 1H, Ar-$\underline{H}$), 6.93 (app. t, *J* = 7.2 Hz, 1H, Ar-$\underline{H}$), 7.10 (d, *J* = 8.4 Hz, 2H, Ar-$\underline{H}$), 7.26 (t, *J* = 7.6 Hz, 2H, Ar-$\underline{H}$). IR (ATR) υ(cm^{-1}): 3004, 2889, 2834,1620, 1591, 1501, 1442, 1405, 1363, 1269, 1246, 1203, 1145, 1077, 1030, 929, 834, 826, 814, 748, 689.

6-Fluoro-3-phenyl-3,4-dihydro-2H-1,3-benzoxazine, ***6*** [2]. The obtained product was purified by crystallization in hexane (1,00 g, 50 % yield). Mp: 48.2 °C ($T_{m(onset)}$, DSC). ^{1}H

NMR (250 MHz, $CDCl_3$) δ(ppm): 4.61 (s, 2H, C_{Ar}-C$\underline{H_2}$-N), 5.34 (s, 2H, O-C$\underline{H_2}$-N), 6.73-6.86 (complex abs., 3H, Ar-H), 6.96 (t, *J* = 7.3 Hz, 1H, Ar-H), 7.11 (d, *J* = 7.8 Hz, 2H, Ar-H), 7.28 (m, 2H, Ar-H). ^{19}F NMR (235.2 MHz, $CDCl_3$) δ(ppm): -123.28. IR (ATR) ν(cm^{-1}): 3068, 2989, 2910, 2862, 1603, 1487, 1431, 1364, 1217, 918, 687.

Experimental details and spectroscopic data for benzoxazines synthesized by method B (using dioxane as solvent).

5-methyl-3-phenyl-3,4-dihydro-2H-1,3-benzoxazine, ***2a*** and *7-methyl-3-phenyl-3,4-dihydro-2H-1,3-benzoxazine,* ***2b*** [3]. The resulting yellow oil was directly subjected to flash column chromatography (silica gel, hexane: EA = 9.5:0.5 v/v as eluent). Total yield for *m*-CH_3: 56 % (1.26 g). (17:83). To identify each *meta* isomer, 2D NMR (COSY, HSQC, HMBC) experiments were performed, being able to determine the mixture isomers of 17:83 (m_5-CH_3(Bz) : m_7-CH_3(Bz)).

5-methyl-3-phenyl-3,4-dihydro-2H-1,3-benzoxazine, ***2a*** ^{1}H RMN (360 MHz, $CDCl_3$) δ(ppm): 2.22 (s, 3H, Ar-CH_3), 4.53 (s, 2H, C_{Ar}-CH_2-N), 5.32 (s, 2H, O-CH_2-N), 6.66-6.78 (m, 1H, Ar-H), 6.85-6.96 (complex abs., 2H, Ar-H), 7.04 (t, 1H, Ar-H),7,11 (d, *J* = 10.8 Hz, 2H, Ar-H), 7.26 (t, *J* = 7.2 Hz, 2H, Ar-H). ^{13}C RMN (90.5 MHz, $CDCl_3$): δ(ppm): 18.41, 49.00, 79.09, 114.83, 118.30, 118.40, 121.50, 122.45, 127.49, 129.40, 135.68, 148.80, 154.65.

7-methyl-3-phenyl-3,4-dihydro-2H-1,3-benzoxazine, ***2b***. ^{1}H RMN (360 MHz, $CDCl_3$) δ (ppm): 2.27 (s, 3H, Ar-CH_3), 4.60 (s, 2H, C_{Ar}-CH_2-N), 5.35 (s, 2H, O-CH_2-N), 6.63 (s, 1H, Ar-H), 6.66-6.78 (m, 1H, Ar-H), 6.85-6.96 (complex abs., 2H, Ar-H), 7,11 (d, *J* = 10.8 Hz, 2H, Ar-H), 7.26 (t, *J* = 7.2 Hz, 2H, Ar-H). ^{13}C RMN (90.5 MHz, $CDCl_3$) δ (ppm): 21.30, 50.42, 79.60, 117.44, 117.93, 118.40, 121.50, 121.87, 126.64, 129.40, 138.02, 148.61, 154.29.

IR (ATR) ν(cm^{-1}) of mixture **2a**/**2b**: 3027, 2361, 1654, 1624, 1600, 1580, 1498, 1386, 1288, 1245, 1199, 1142, 1114, 1032, 982, 956, 894, 797, 758, 695, 631. HRMS (ESI/Q-TOF) m/z: [M+Na^+] Calcd for $C_{15}H_{15}NONa$ 248.2755; Found 248.1036.

Fluoro-3-phenyl-3,4-dihydro-2H-1,3-benzoxazines ***7*** *and* ***8***. The resulting yellow oil was directly subjected to flash column chromatography (silica gel, hexane: EA = 9.5:0.5 v/v as eluent). Total yield for *m*-F(Bz): 19 % (190 mg) (Product **7**: yellow oil 13 % yield (130 mg) and product **8**: yellow oil 6% yield (60 g)).

5-Fluoro-3-phenyl-3,4-dihydro-2H-1,3-benzoxazine, ***7*** [4]. ^{1}H NMR (250 MHz, $CDCl_3$) δ(ppm): 4.62 (s, 2H, C_{Ar}-C$\underline{H_2}$-N), 5.38 (s, 2H, 2H, O-C$\underline{H_2}$-N), 6.56-6.68 (complex abs.,

2H, Ar-H), 6.94-7.02 (complex abs., 2H, Ar-H), 7.14 (d, *J* = 7.5 Hz, Ar-H), 7.31 (t, *J* = 7.5 Hz, 2H, Ar-H). ^{19}F NMR (235.2 MHz, $CDCl_3$) δ(ppm): -114.15. IR (ATR) ν(cm^{-1}): 1622, 1599, 1500, 1436, 1264, 1135, 1103, 1031, 991, 964, 631.

7-Fluoro-3-phenyl-3,4-dihydro-2H-1,3-benzoxazine, ***8*** [2]. ^{1}H NMR (250 MHz, $CDCl_3$) δ(ppm): 4.66 (s, 2H, C_{Ar}-CH_2-N), 5.36 (s, 2H, O-CH_2-N), 6.58-6.64 (complex abs., 2H, Ar-H), 6.96 (t, *J* = 7.5 Hz, 1H, Ar-H), 7.06 (m, 1H, Ar-H), 7.13 (d, *J* = 7.5 Hz, 2H, Ar-H), 7.29 (t, *J* = 7.7, 2H, Ar-H). ^{19}F NMR (235.2 MHz, $CDCl_3$) δ(ppm): -119.72. IR (ATR) ν(cm^{-1}): 1623, 1598, 1495, 1467, 1369, 1345, 1256, 1234, 1160, 1020, 985, 949, 777, 754, 694, 631.

S2. 6-Methyl-3-phenyl-3,4-dihydro-2*H*-1,3-benzoxazine, 1 [1]

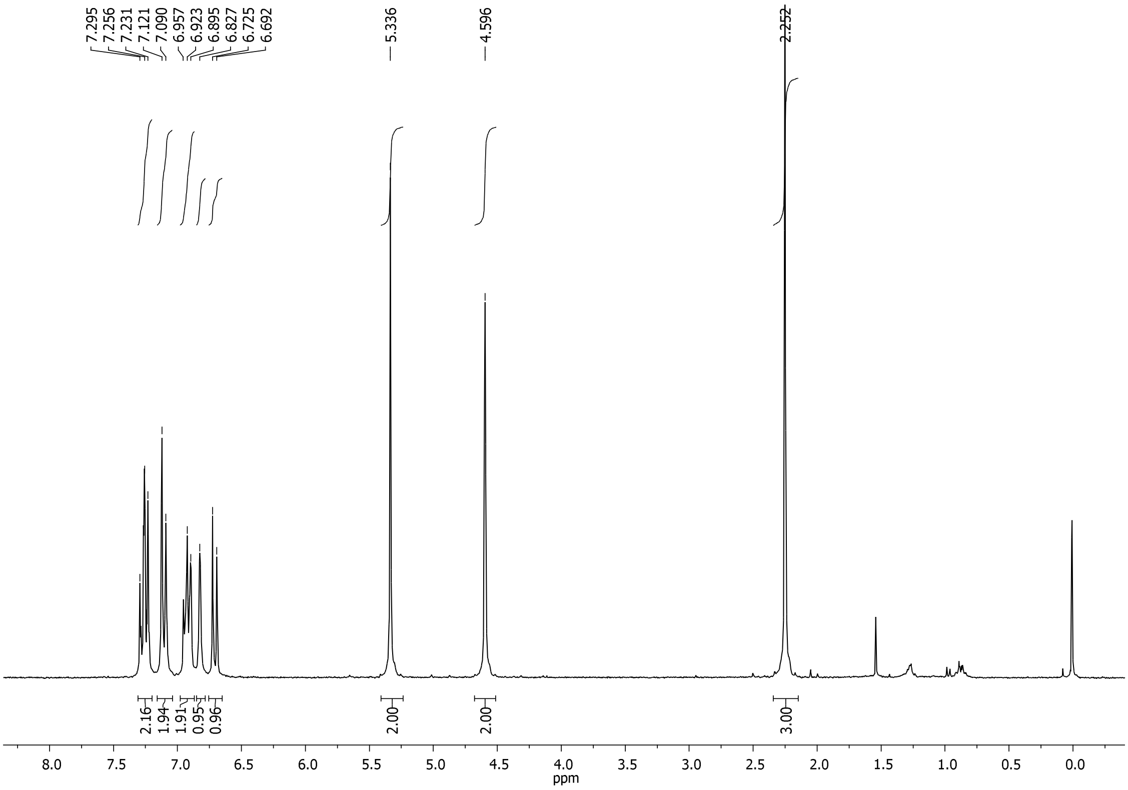

Figure S1. ^{1}H NMR (250 MHz) spectrum of **1** in $CDCl_3$.

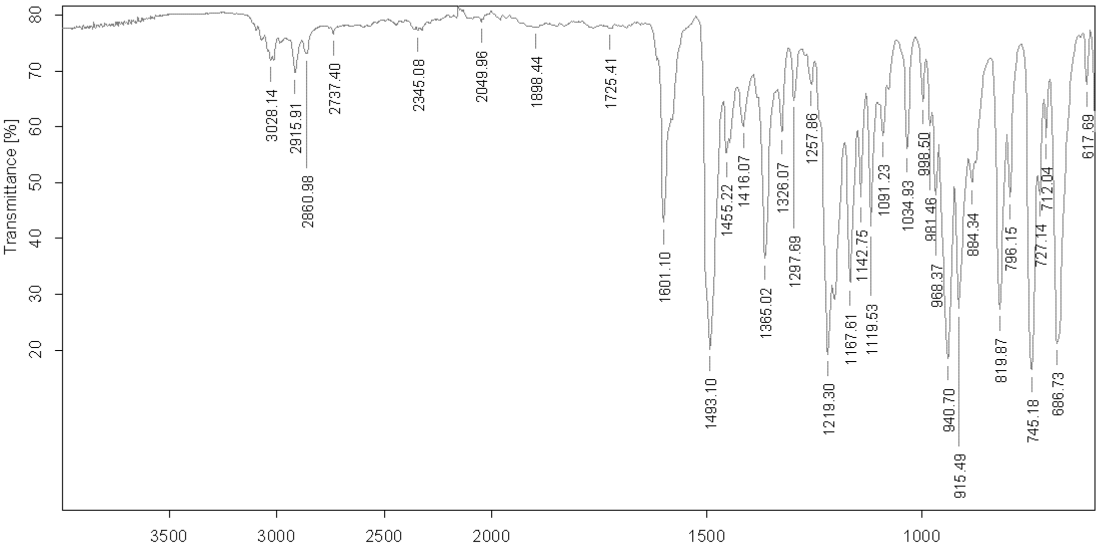

Figure S2. IR (ATR) υ (cm^{-1}) of **1**.

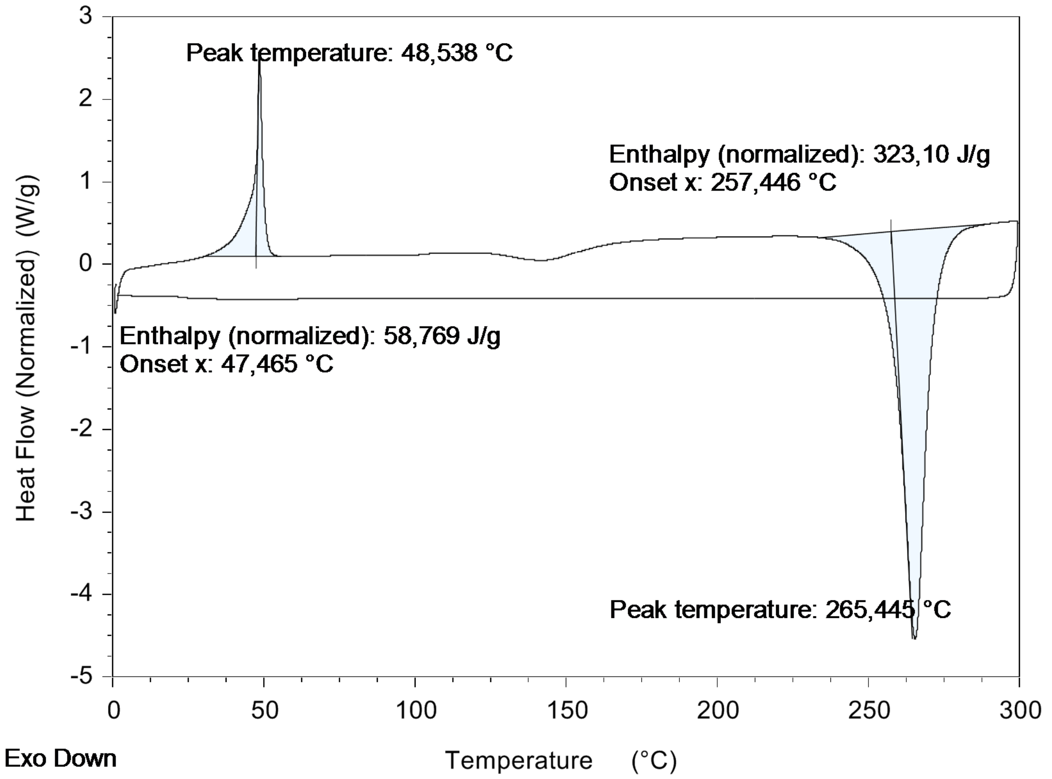

Figure S3. DSC thermogram of **1**.

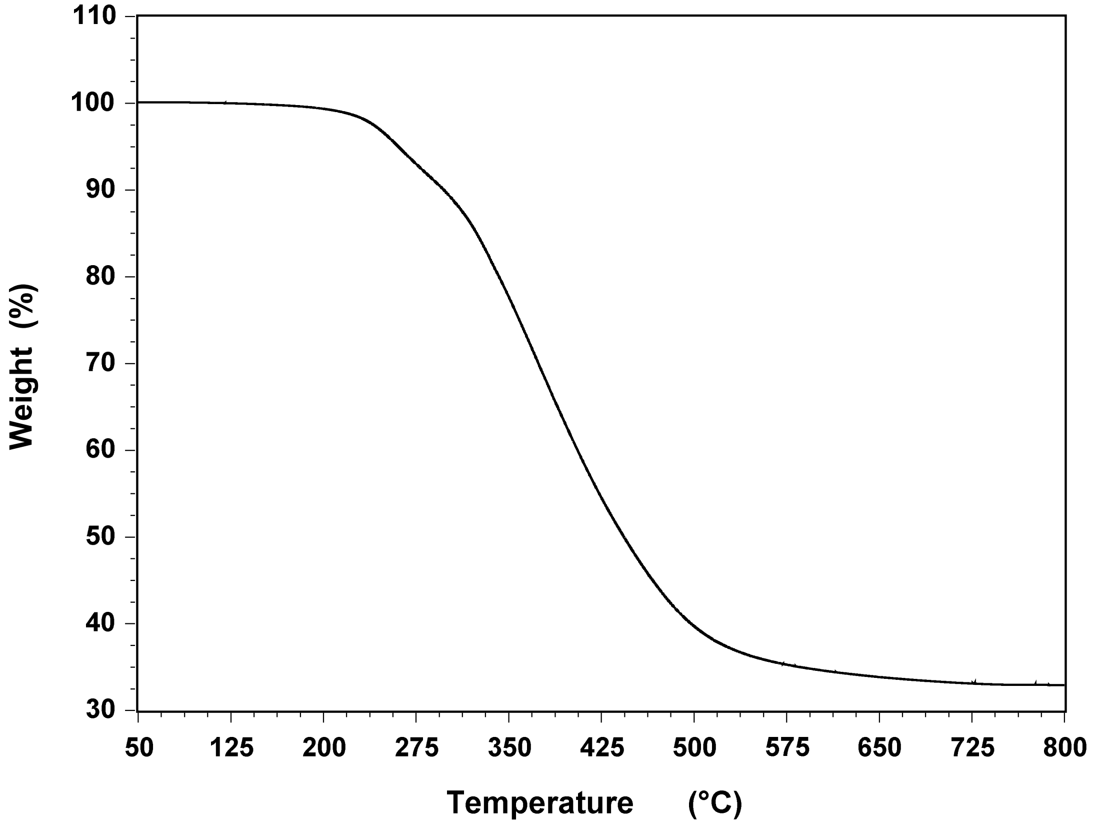

Figure S4. TGA thermogram of **1**.

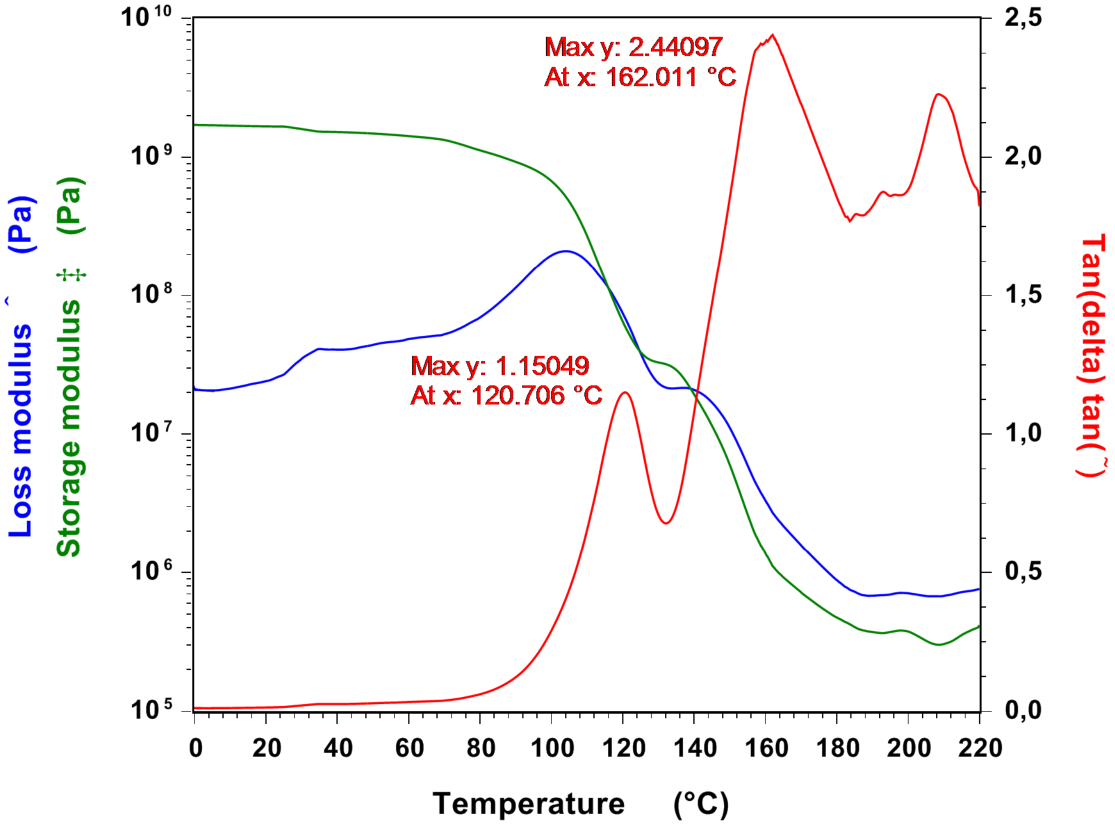

Figure S5. DMA thermogram of **1**.

S3. 5-Methyl-3-phenyl-3,4-dihydro-2*H*-1,3-benzoxazine, 2a and 7-Methyl-3-phenyl-3,4-dihydro-2*H*-1,3-benzoxazine, 2b

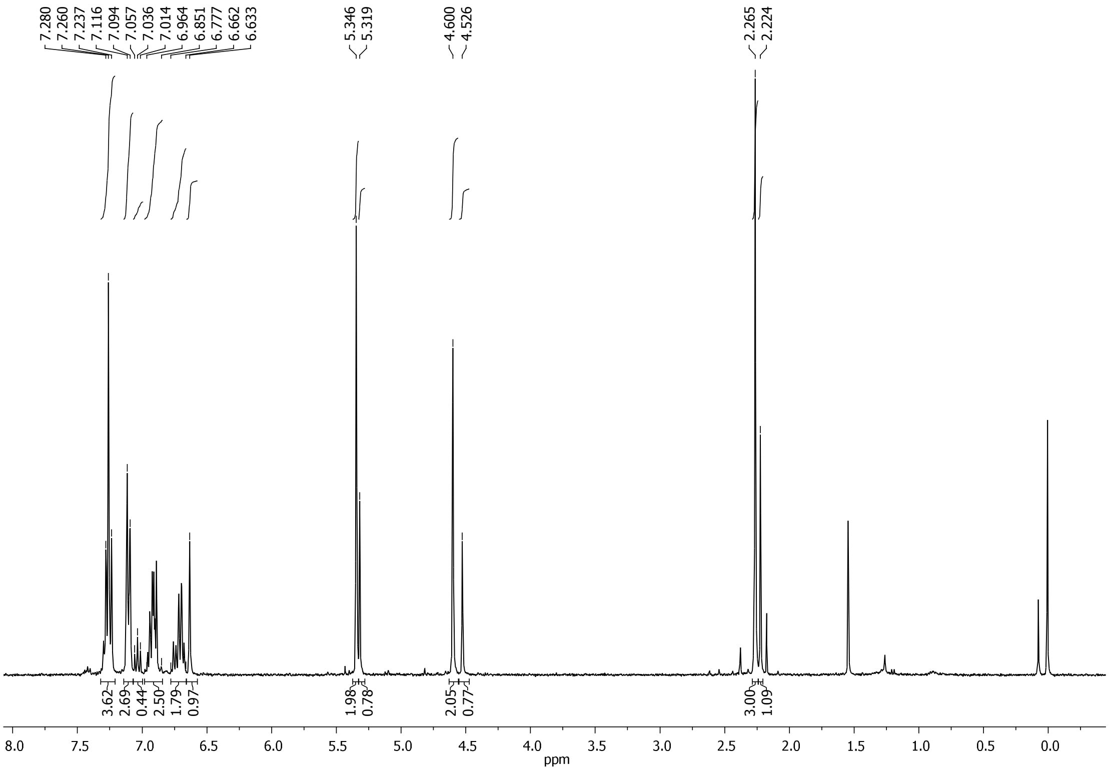

Figure S6. ^{1}H NMR (360 MHz) spectrum of **2a** and **2b** in $CDCl_3$.

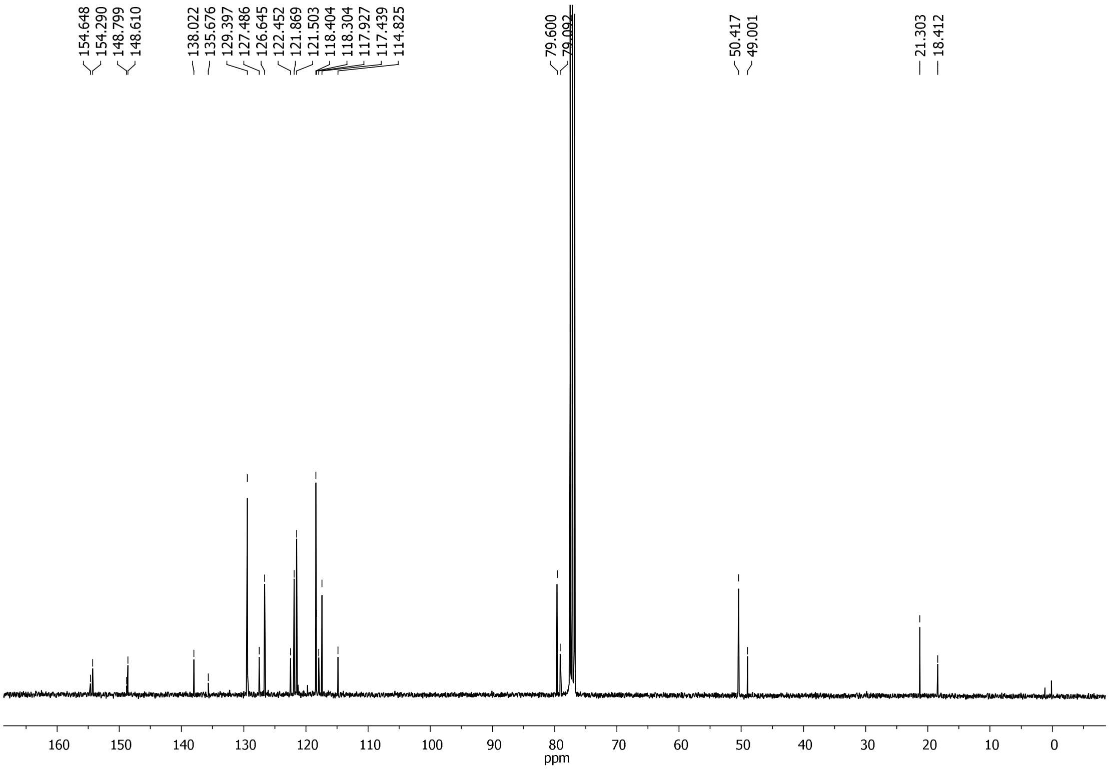

Figure S7. ^{13}C NMR (90.5 MHz) spectrum of **2a** and **2b** in $CDCl_3$.

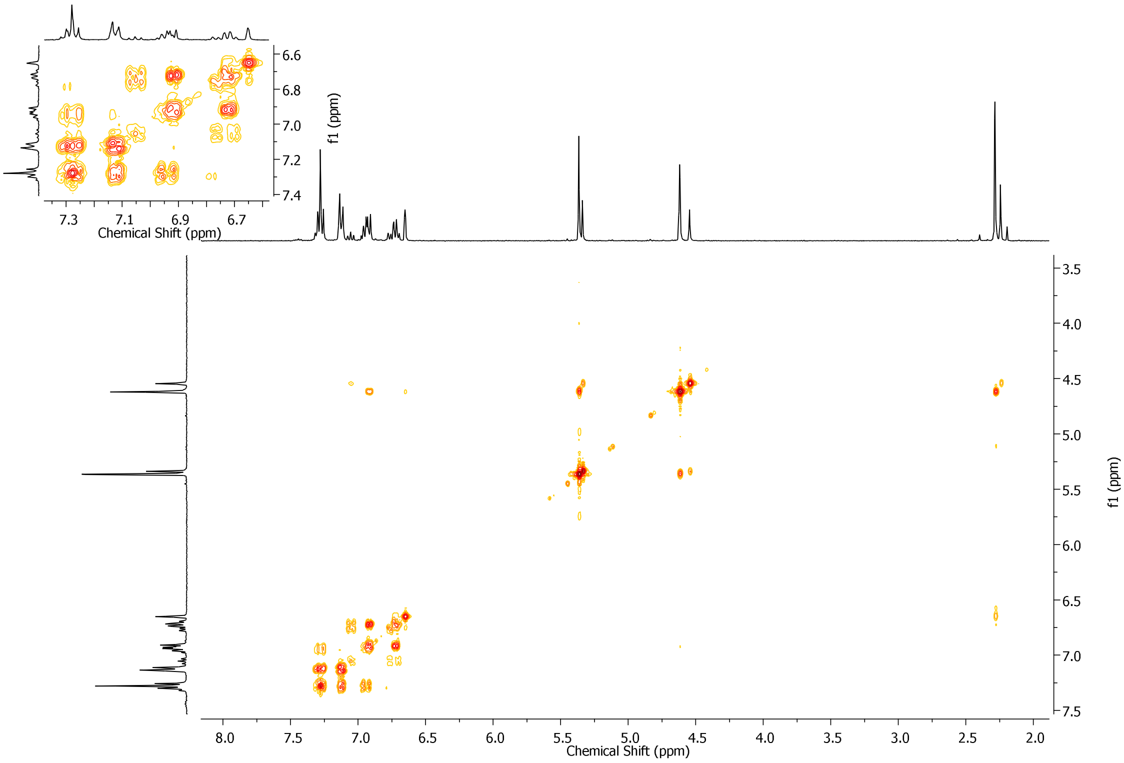

Figure S8. ^1H-^1H COSY NMR (360 MHz) spectrum of **2a** and **2b** in $CDCl_3$.

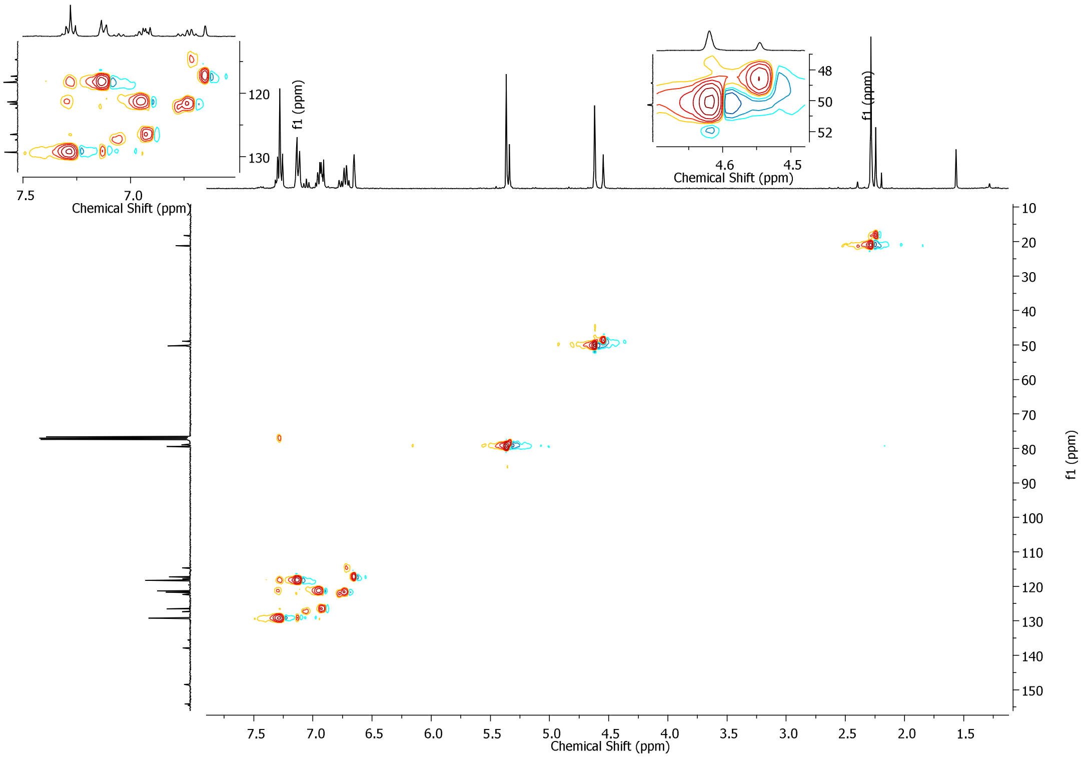

Figure S9. HSQC NMR (360/90.5 MHz) spectrum of **2a** and **2b** in $CDCl_3$.

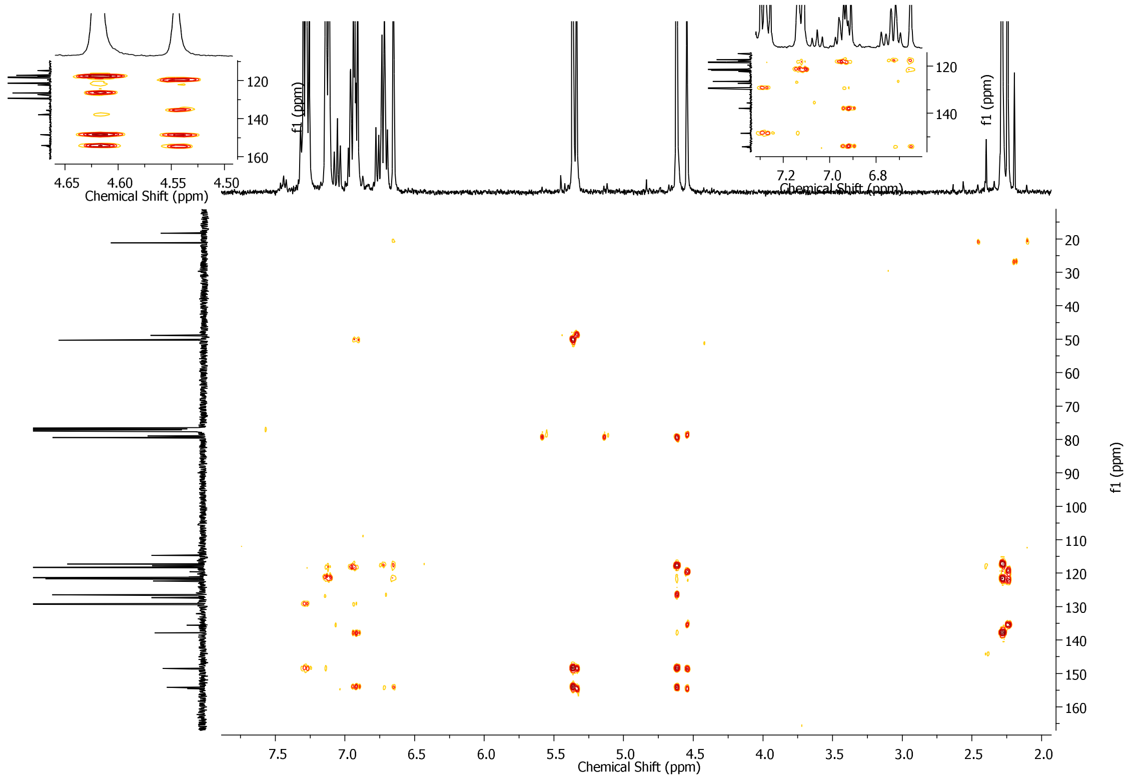

Figure S10. HMBC NMR (360/90.5) spectrum of **2a** and **2b** in $CDCl_3$.

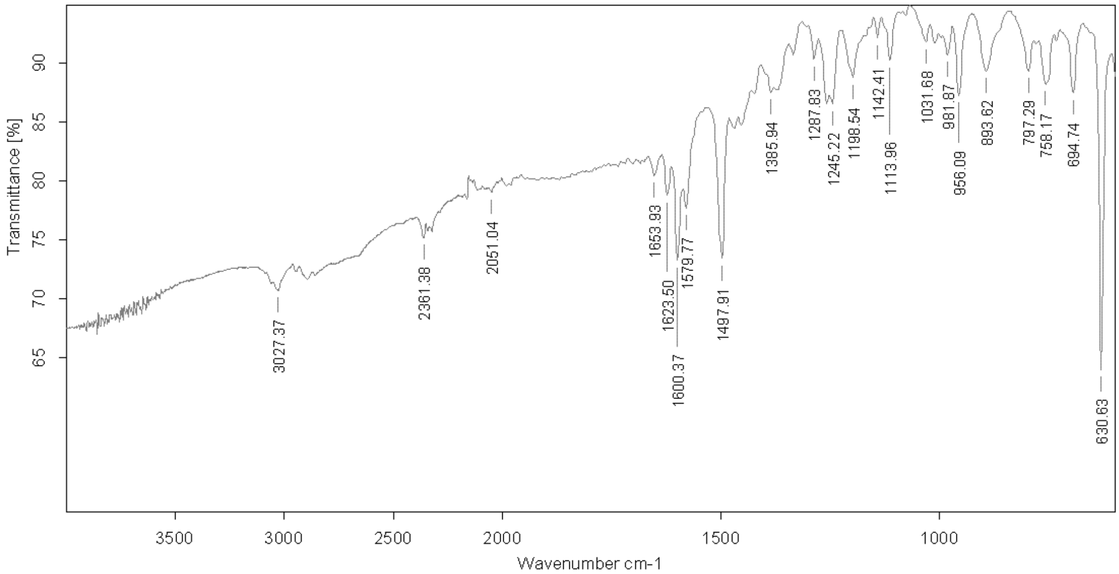

Figure S11. IR (ATR) υ (cm^{-1}) of **2a** and **2b**.

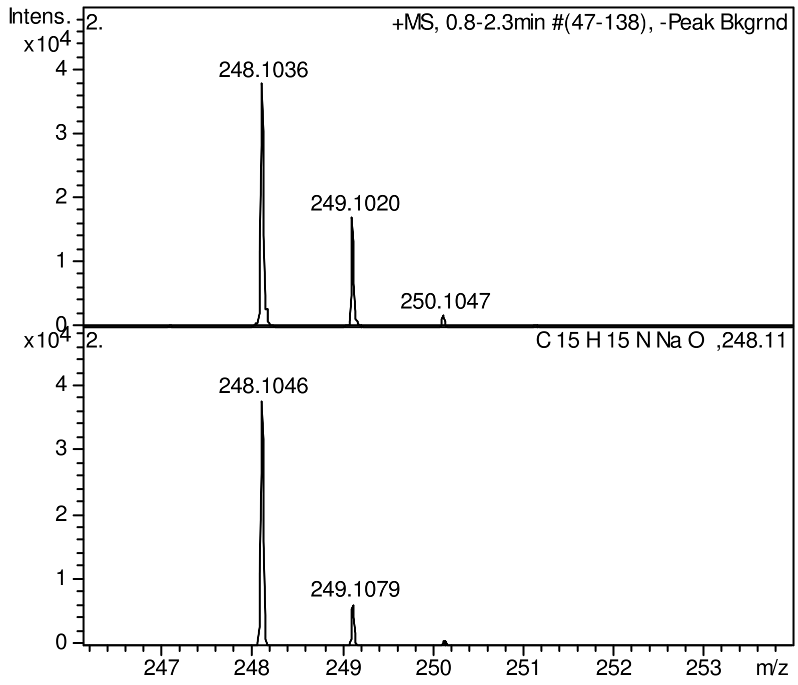

Figure S12. HRMS (ESI/Q-TOF) [M+H+] of **2a** and **2b**.

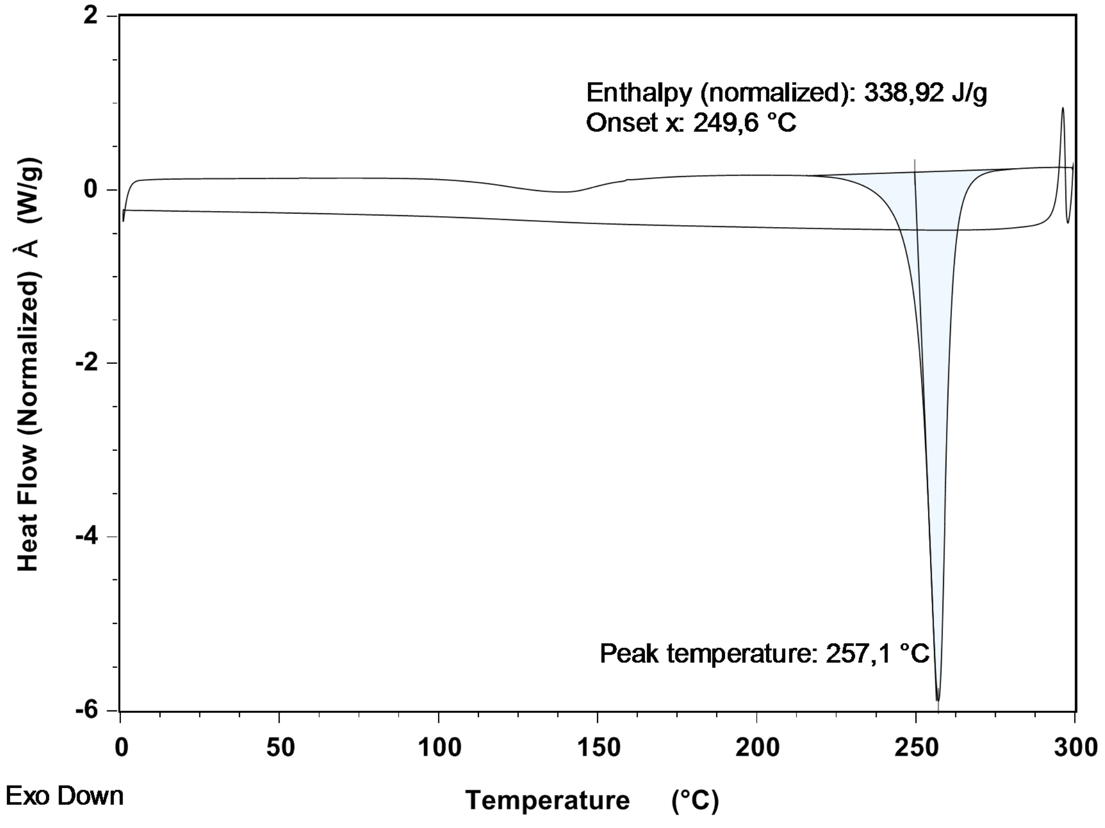

Figure S13. DSC thermogram of **2a** and **2b**.

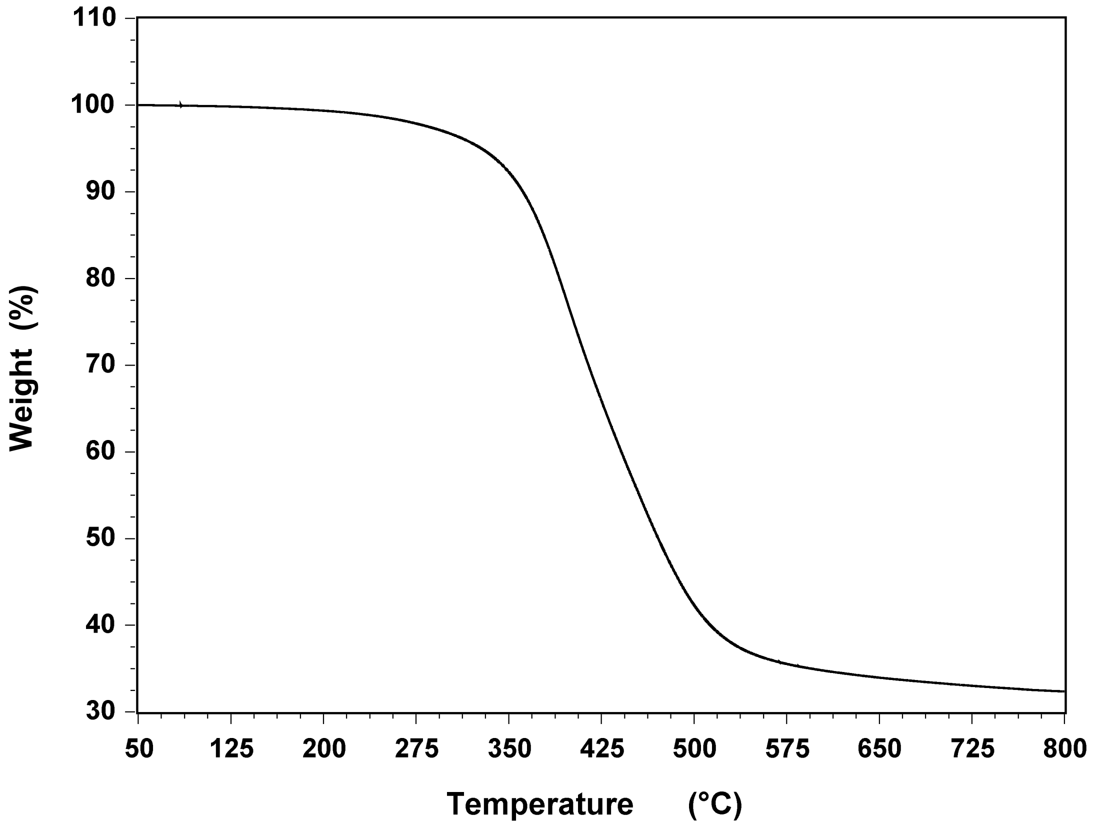

Figure S14. TGA thermogram of **2a** and **2b**.

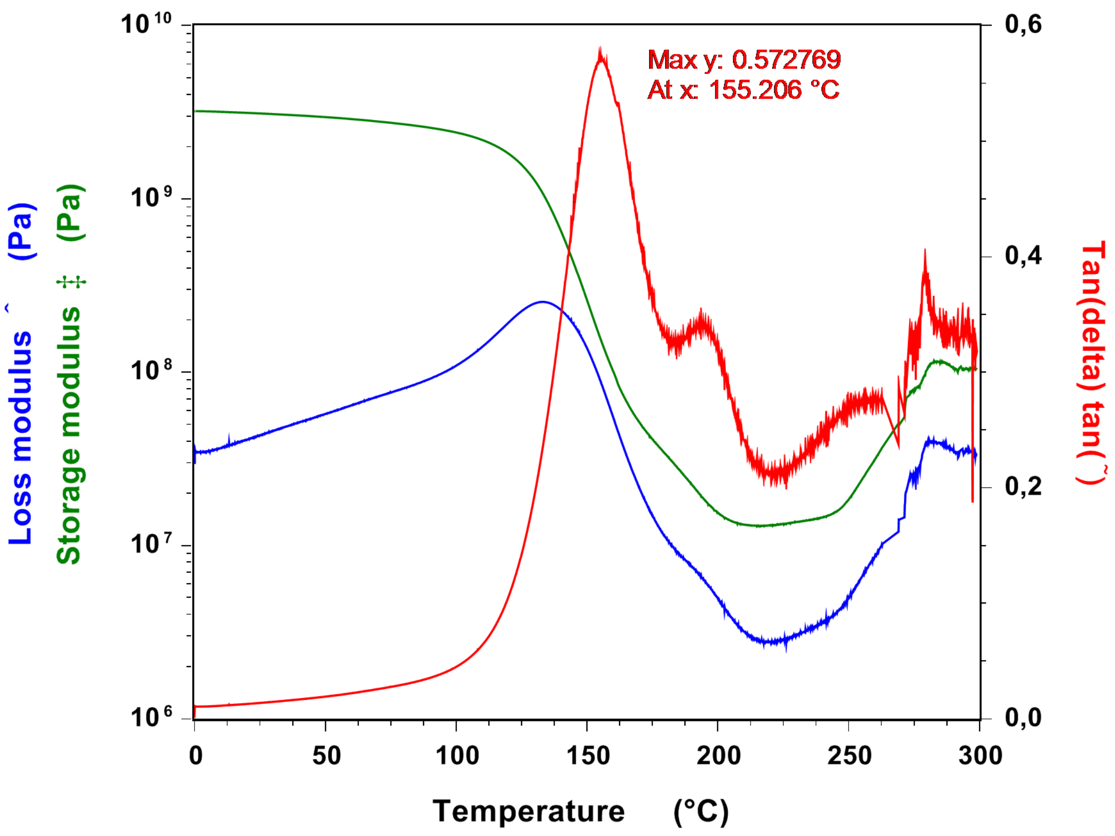

Figure S15. DMA thermogram of **2a** and **2b**.

S4. 6-Methoxy-3-phenyl-3,4-dihydro-2*H*-1,3-benzoxazine, 3 [1]

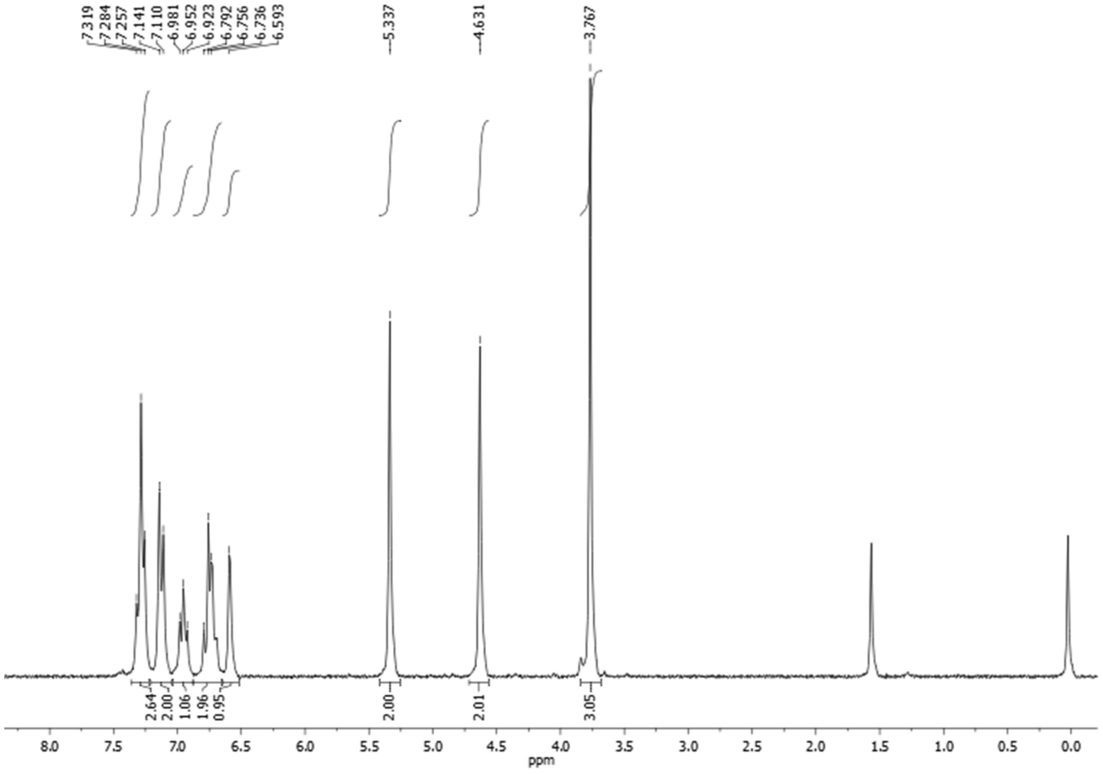

Figure S16. ^{1}H NMR (250 MHz) spectrum of **3** in $CDCl_3$.

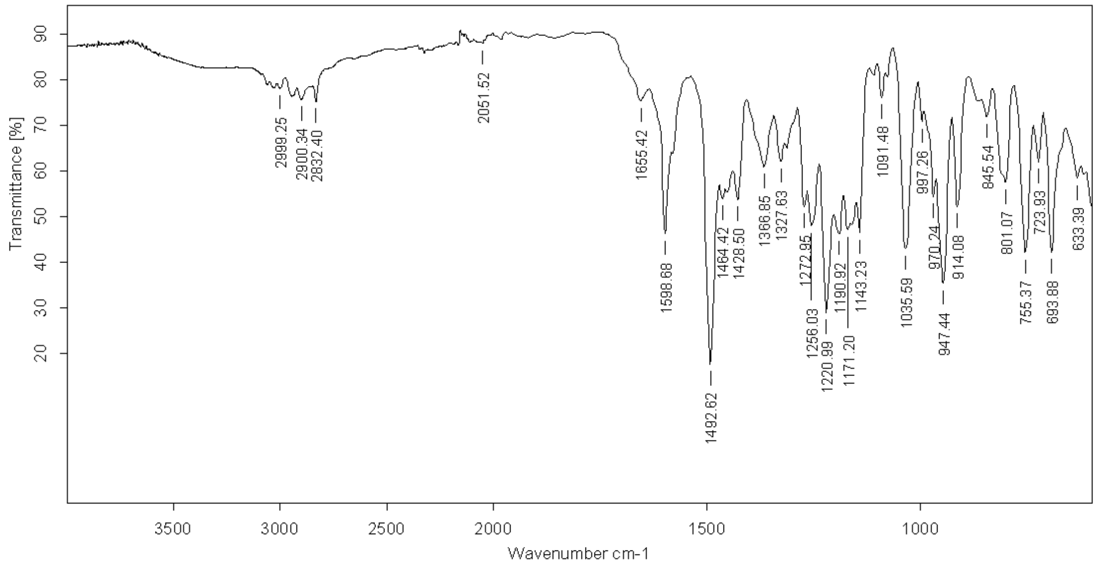

Figure S17. IR (ATR) υ (cm^{-1}) of **3**.

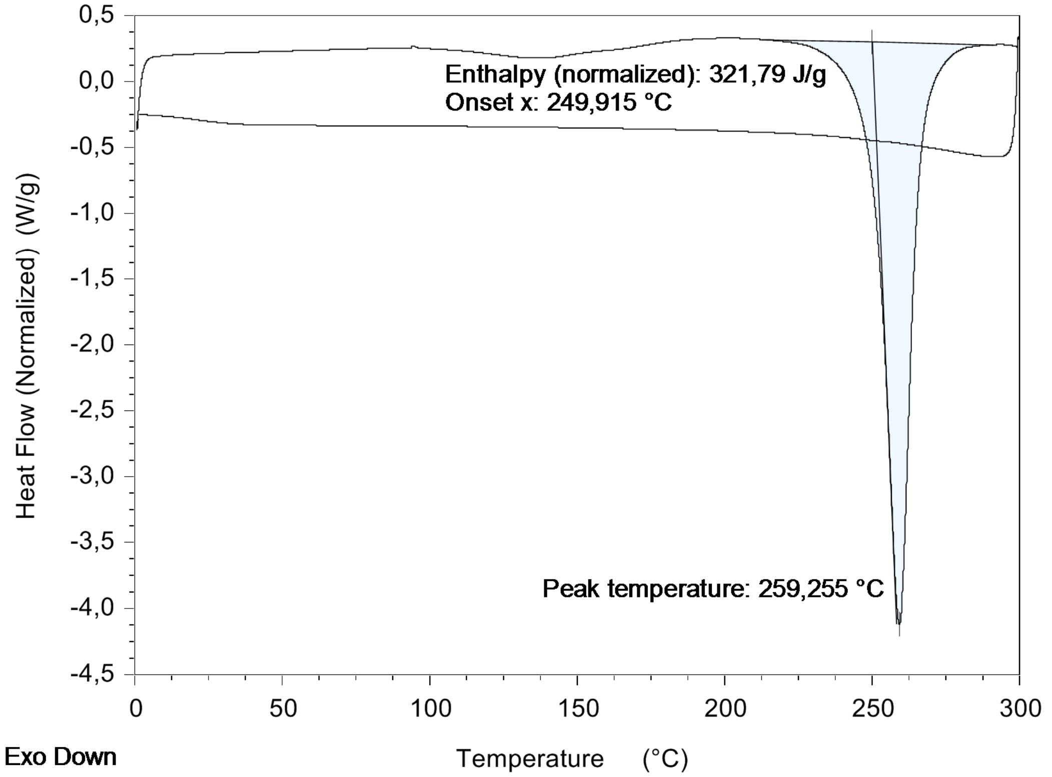

Figure S18. DSC thermogram of **3**.

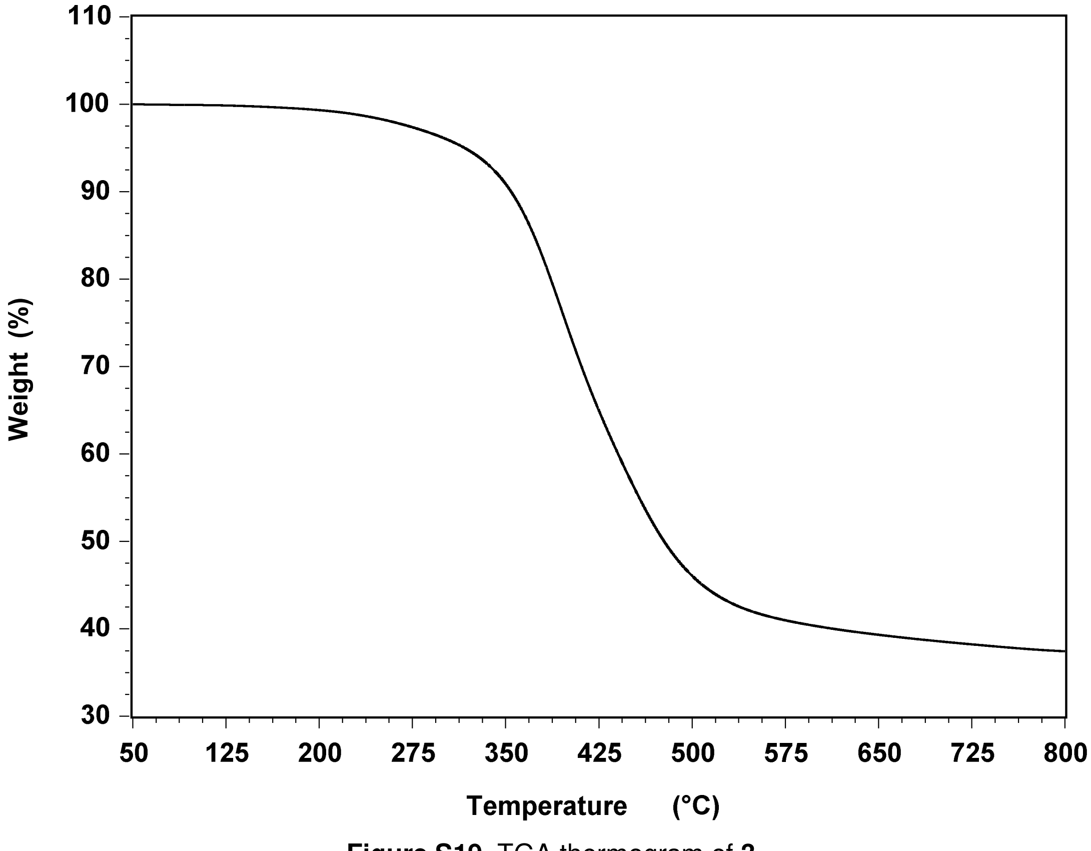

Figure S19. TGA thermogram of **3**.

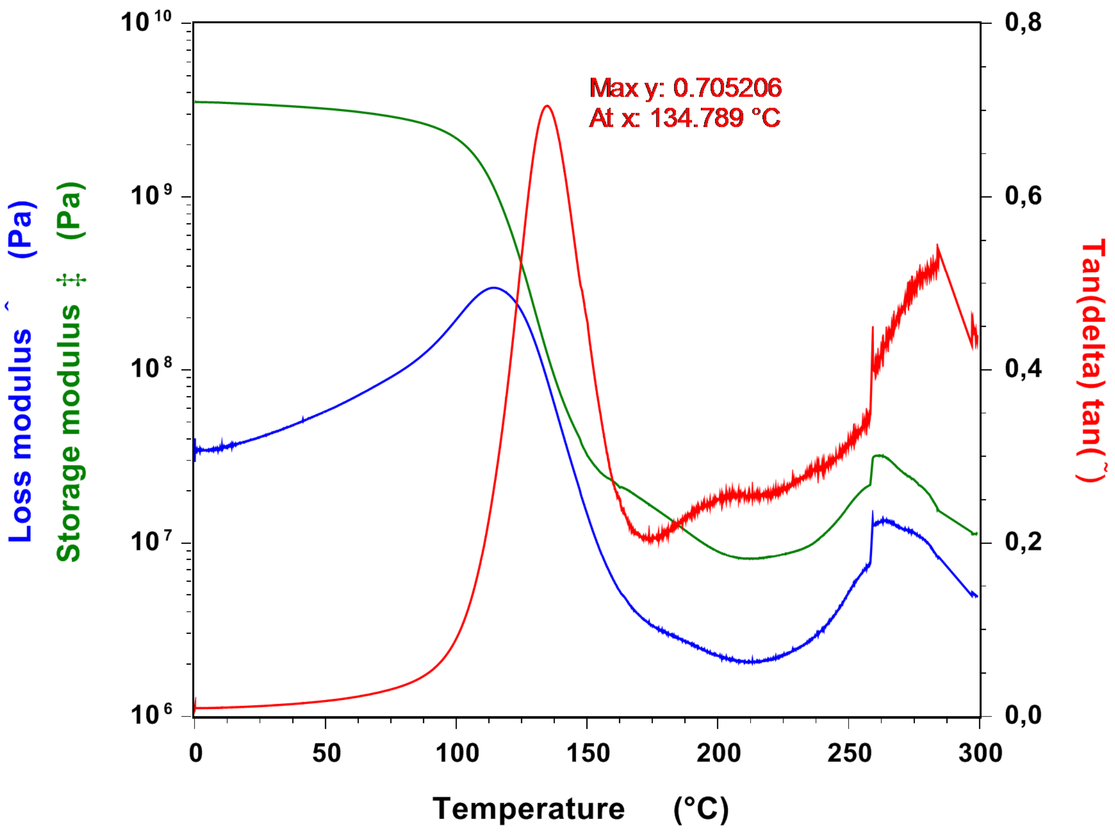

Figure S20. DMA thermogram of **3**.

S5. 5-Methoxy-3-phenyl-3,4-dihydro-2*H*-1,3-benzoxazine, 4 [2]

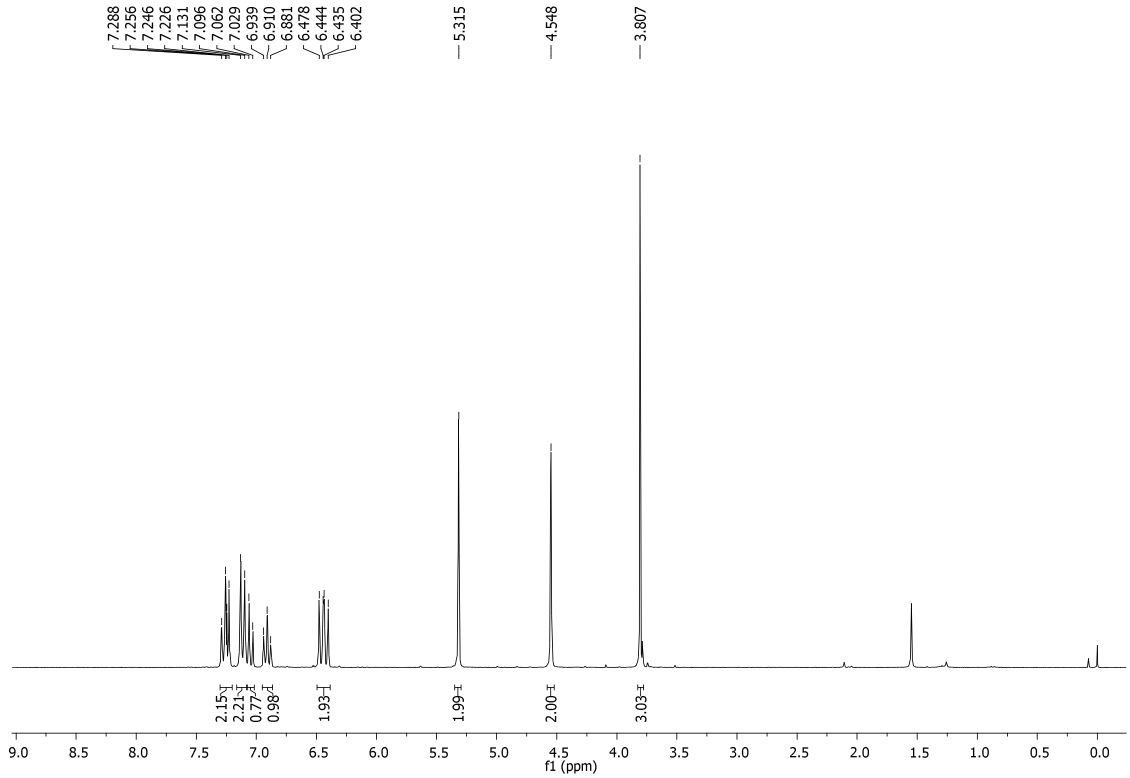

Figure S21. ^{1}H NMR (250 MHz) spectrum of **4** in $CDCl_3$.

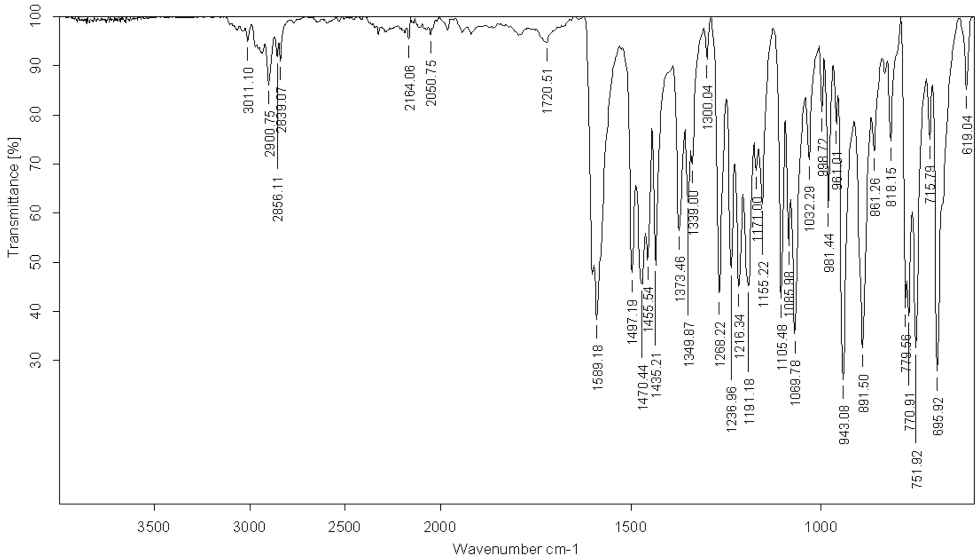

Figure S22. IR (ATR) υ (cm^{-1}) of **4**.

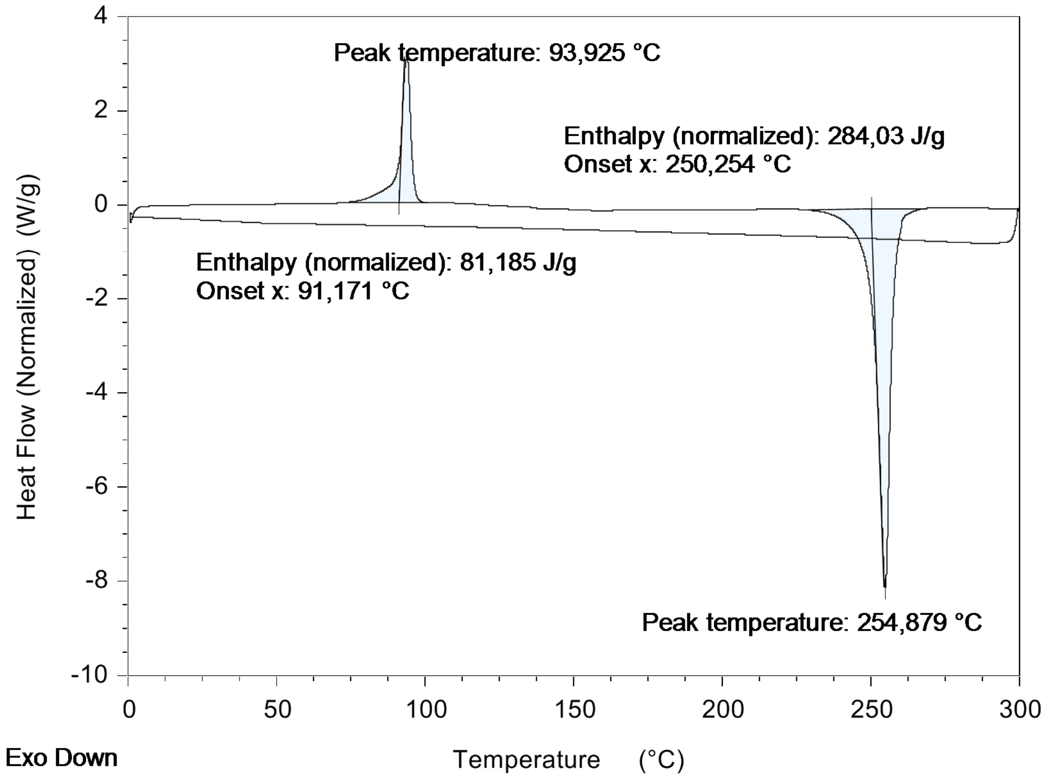

Figure S23. DSC thermogram of **4**.

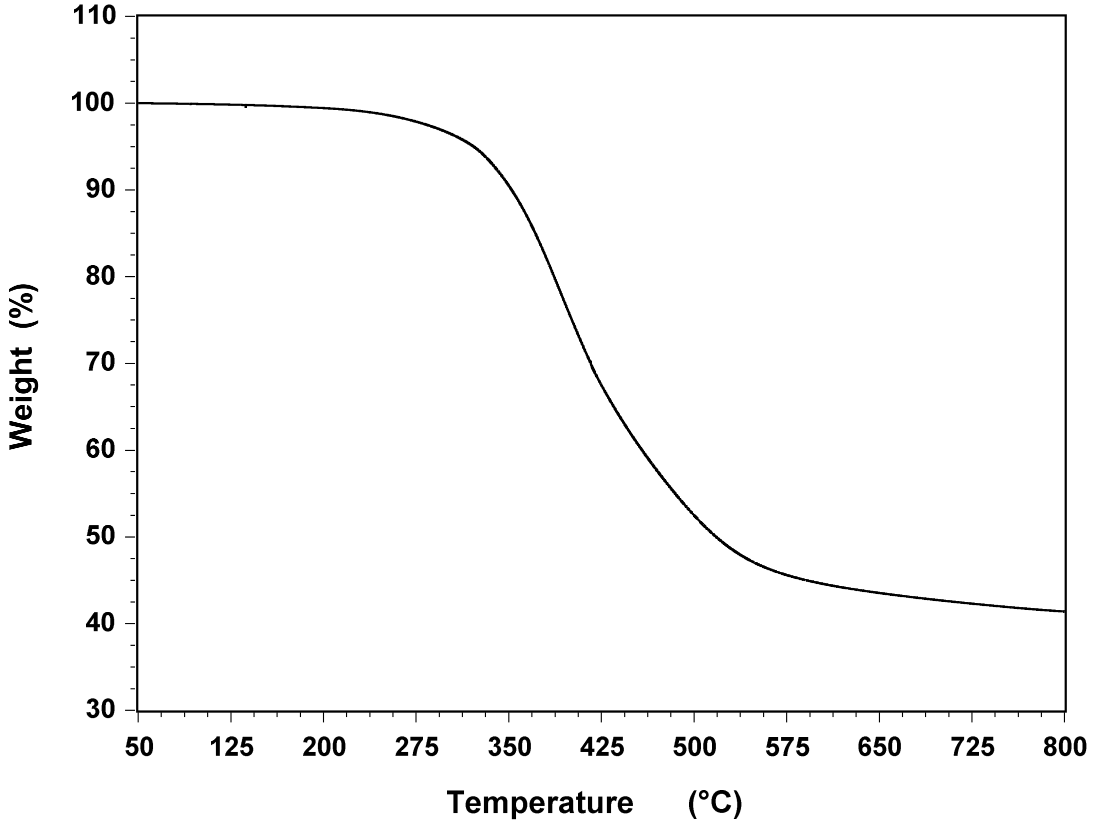

Figure S24. TGA thermogram of **4**.

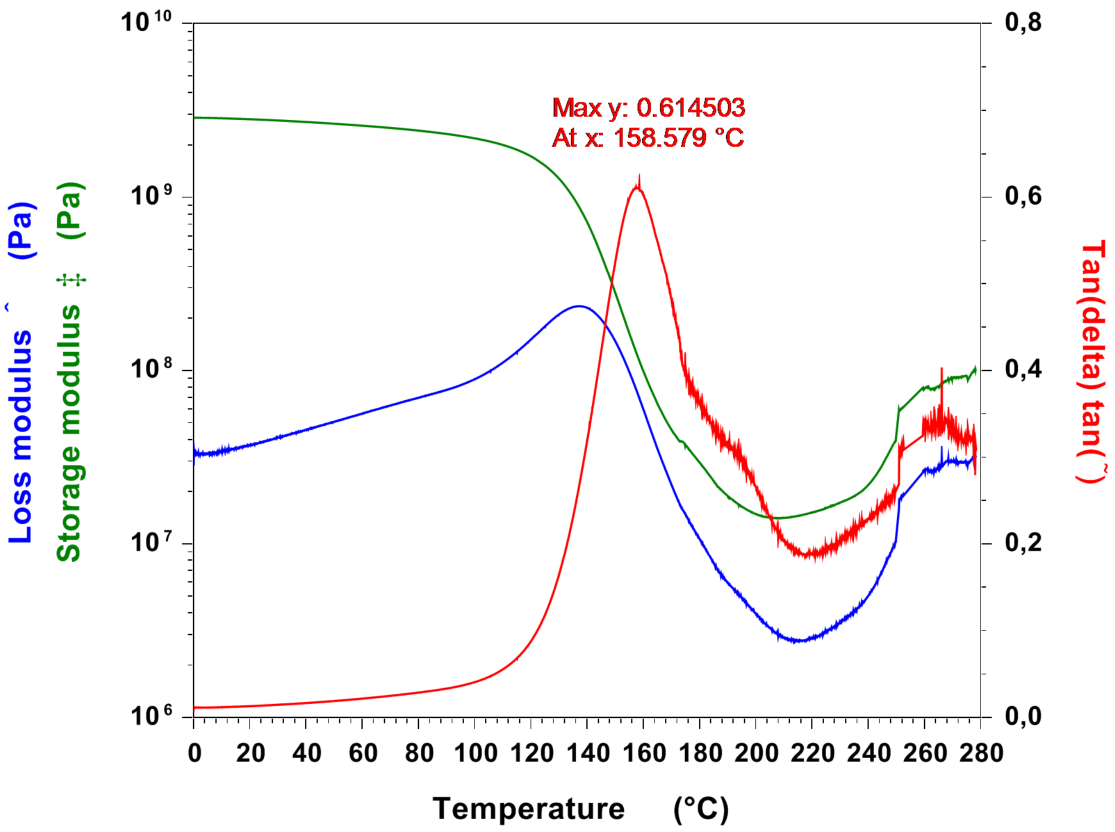

Figure S25. DMA thermogram of **4**.

S6. 7-Methoxy-3-phenyl-3,4-dihydro-2*H*-1,3-benzoxazine, 5 [2]

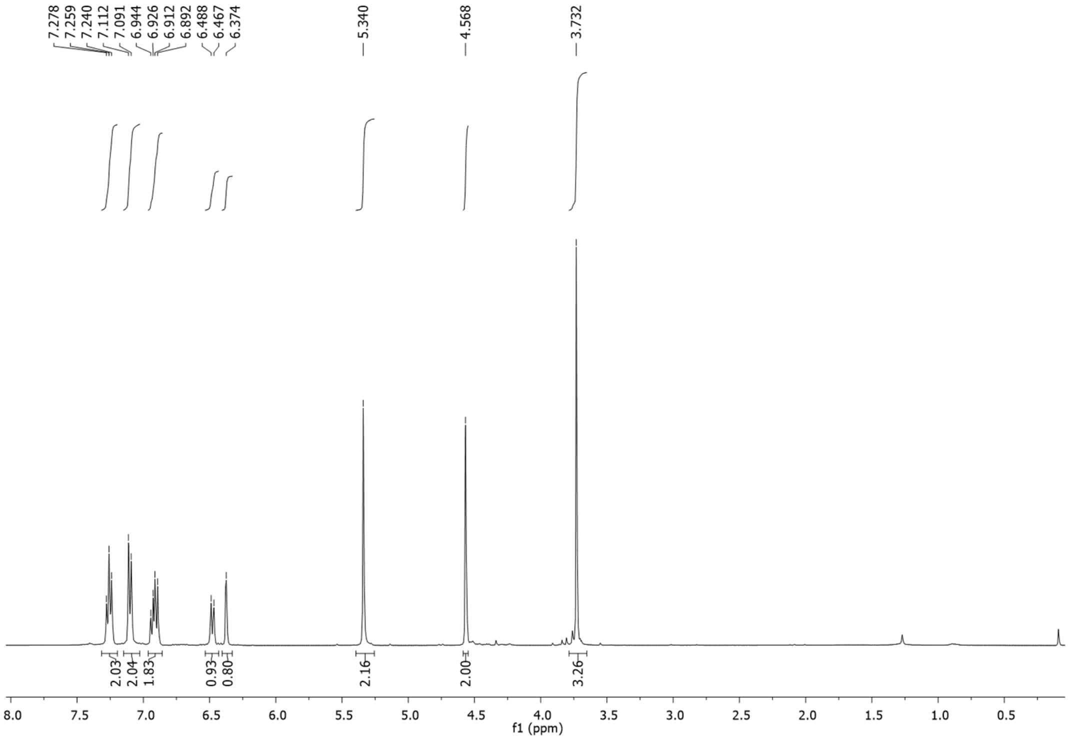

Figure S26. ^1H NMR (400 MHz) spectrum of **5** in $CDCl_3$.

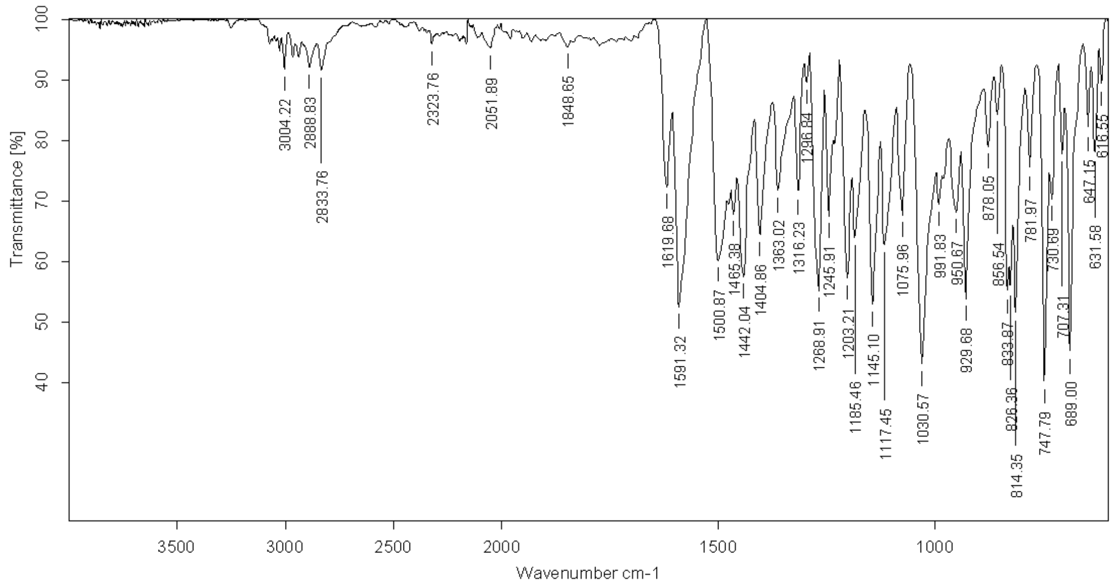

Figure S27. IR (ATR) υ (cm^{-1}) of **5**.

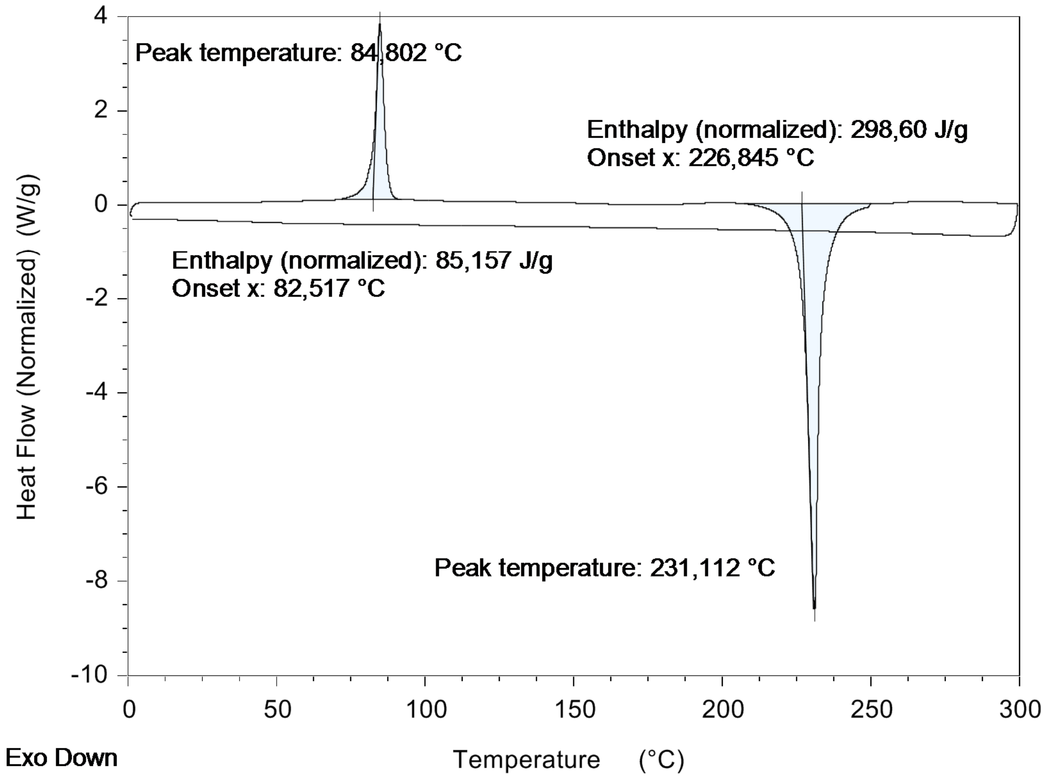

Figure S28. DSC thermogram of **5**.

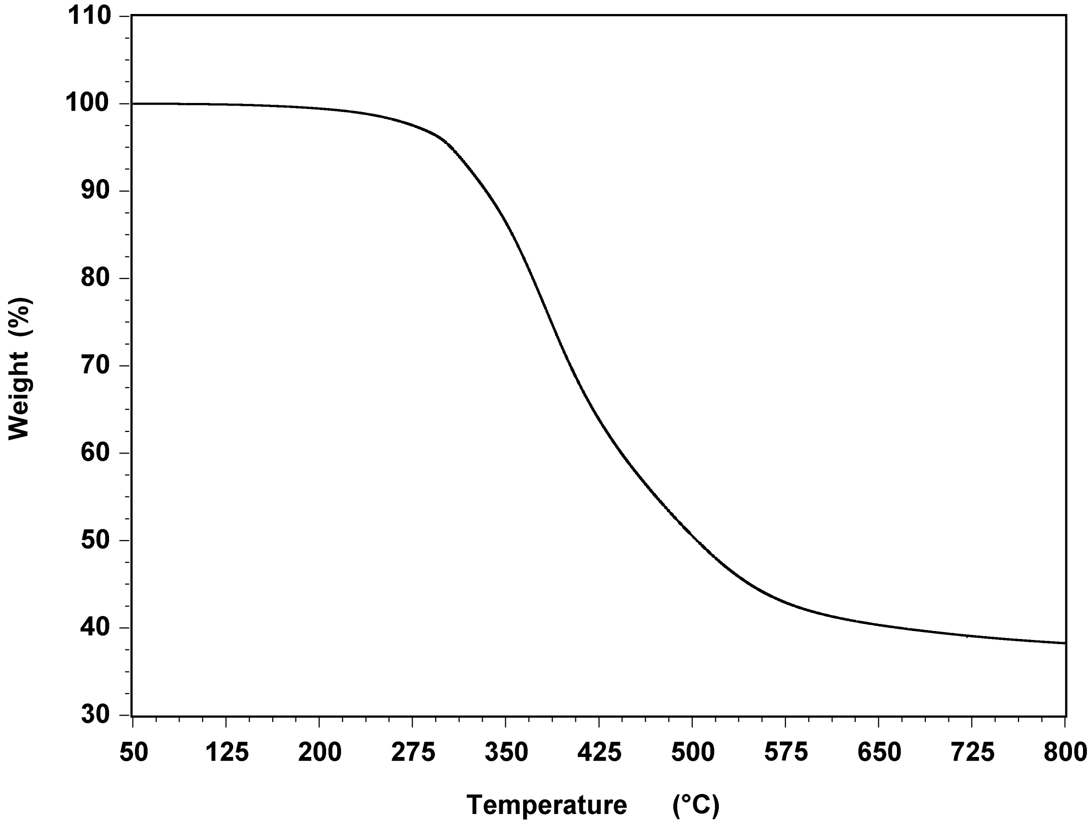

Figure S29. TGA thermogram of **5**.

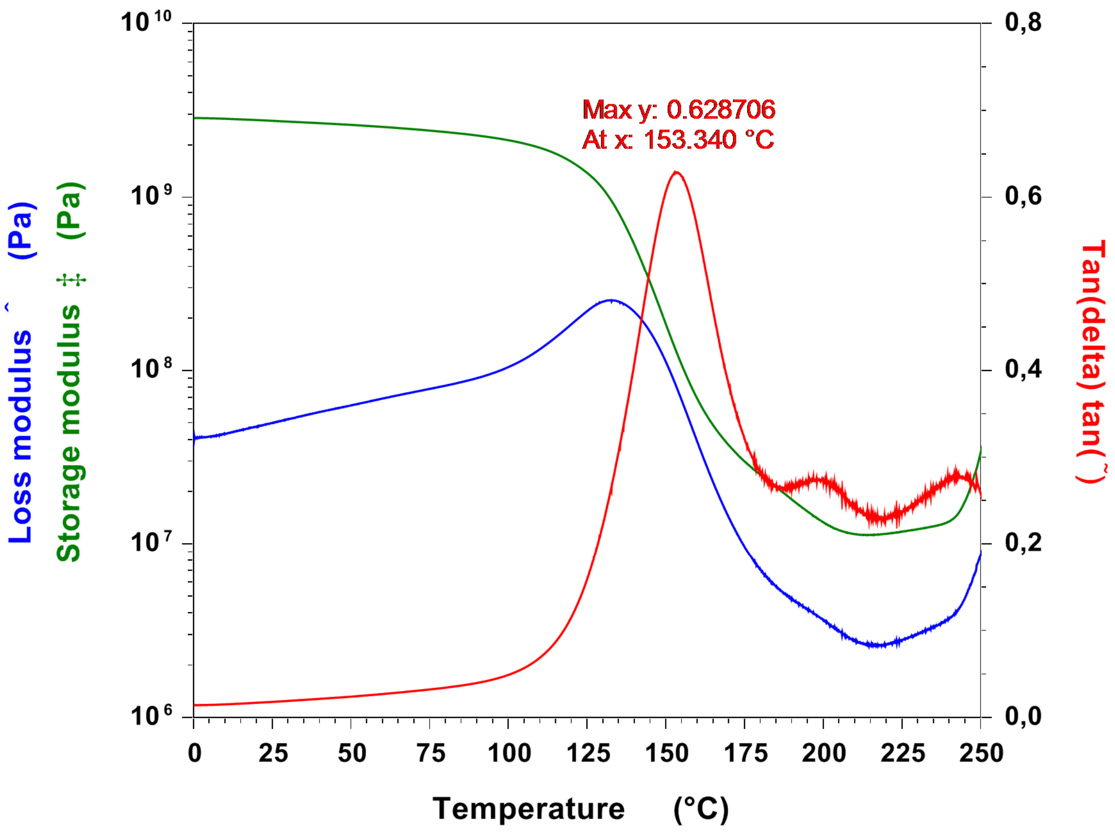

Figure S30. DMA thermogram of **5**.

S7. 6-Fluoro-3-phenyl-3,4-dihydro-2*H*-1,3-benzoxazine, 6 [2]

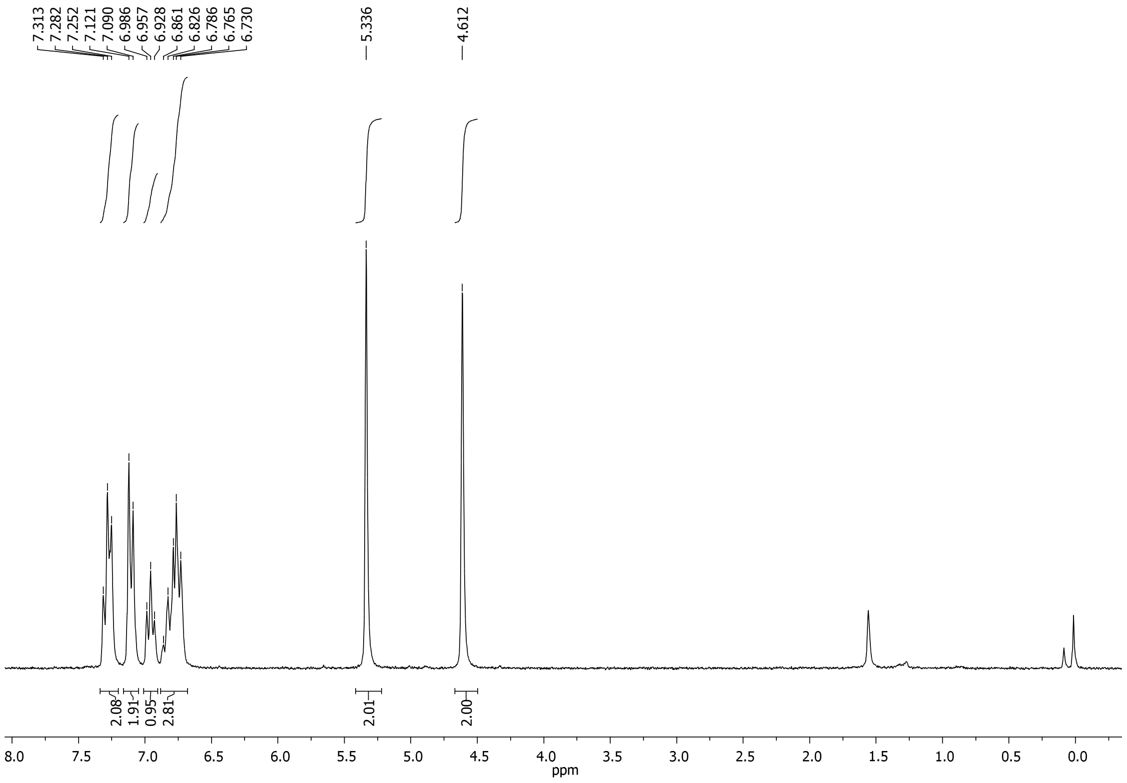

Figure S31. ^{1}H NMR (250 MHz) spectrum of **6** in $CDCl_3$.

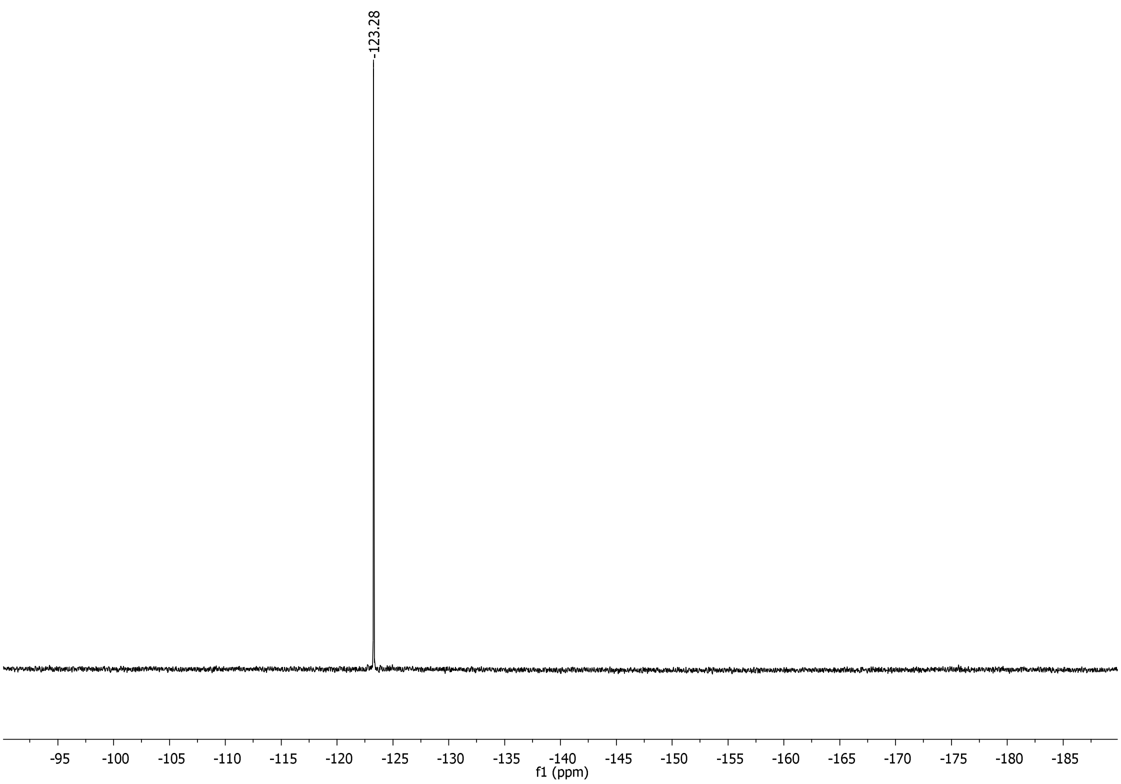

Figure S32. ^{19}F NMR (235.2 MHz) spectrum of **6** in $CDCl_3$.

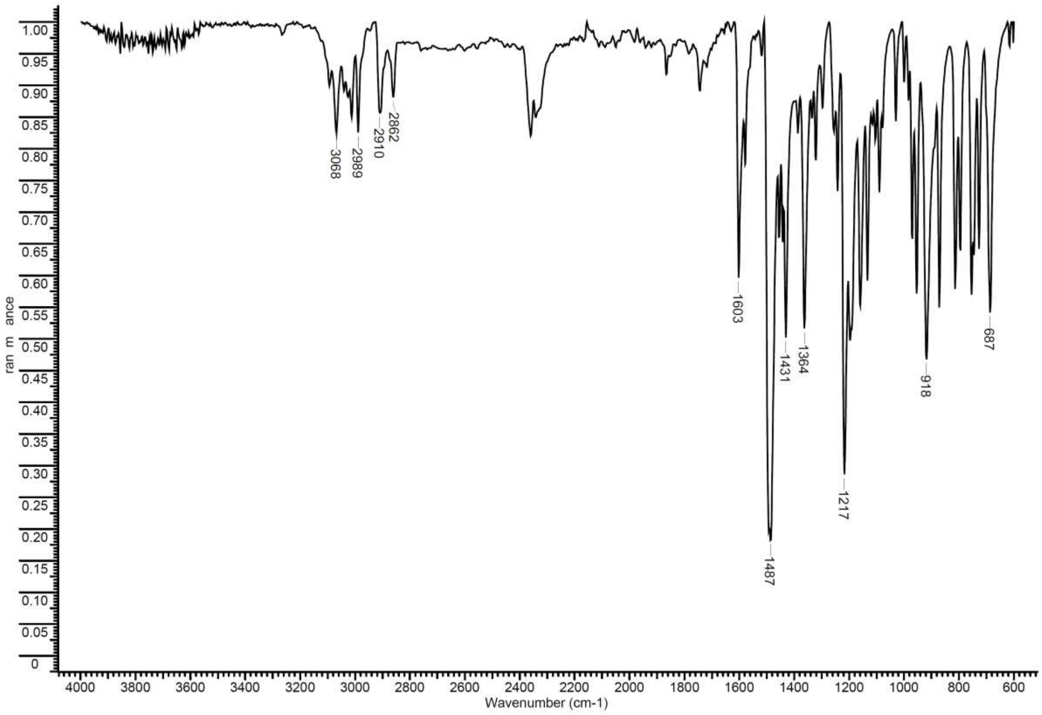

Figure S33. IR (ATR) υ (cm^{-1}) of **6**.

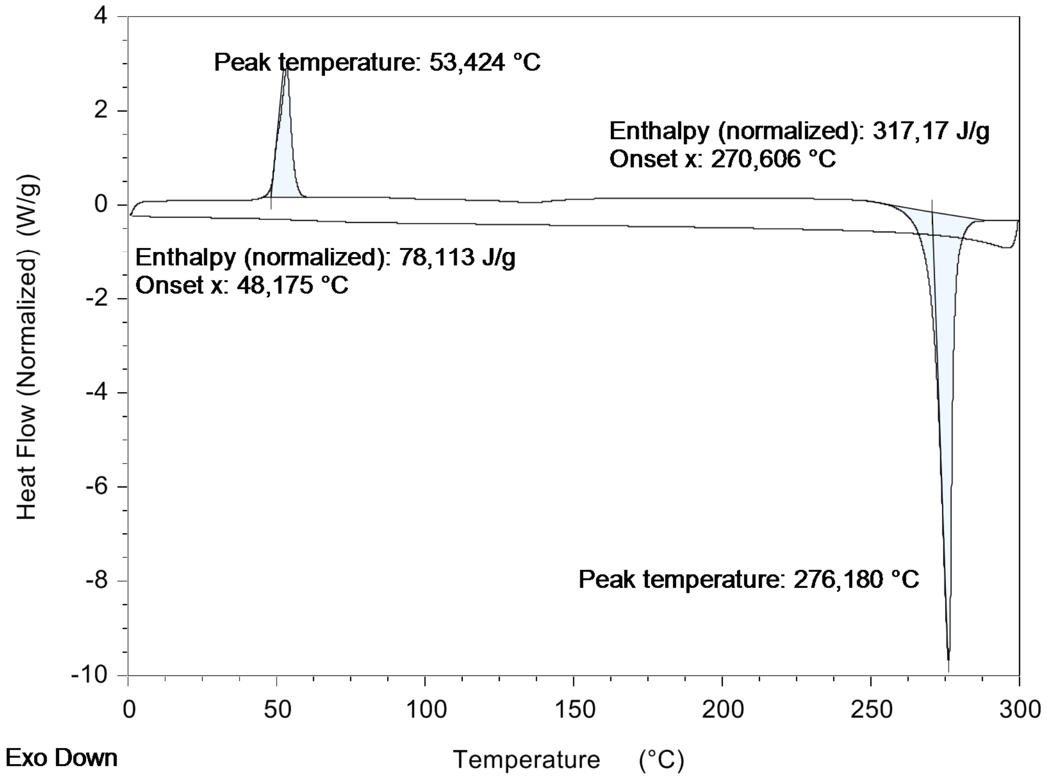

Figure S34. DSC thermogram of **6**.

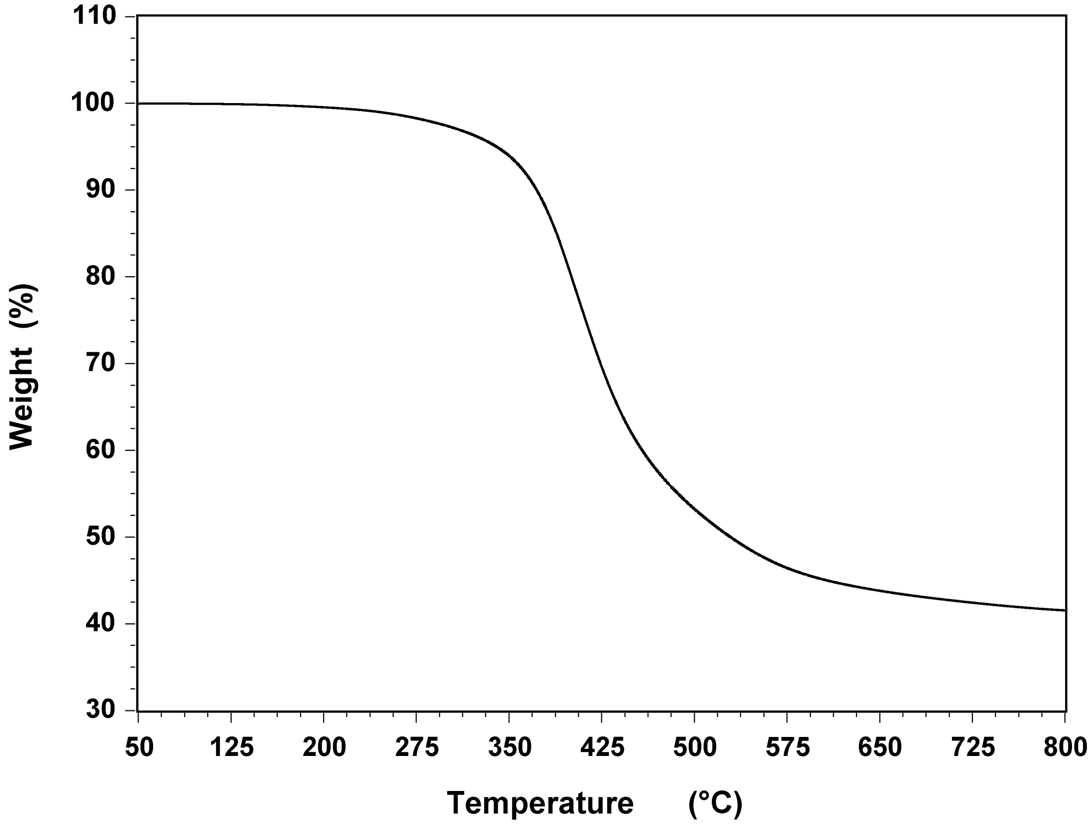

Figure S35. TGA thermogram of **6**.

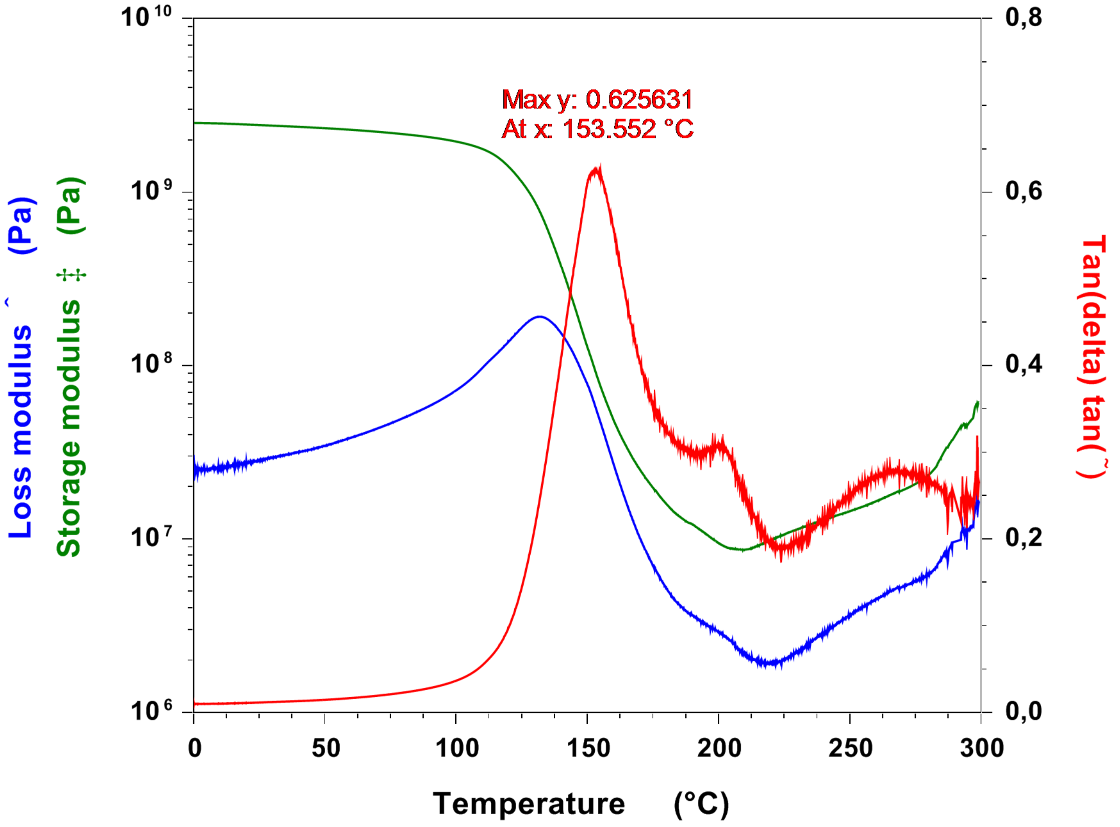

Figure S36. DMA thermogram of **6**.

S8. 5-Fluoro-3-phenyl-3,4-dihydro-2*H*-1,3-benzoxazine, 7 [4]

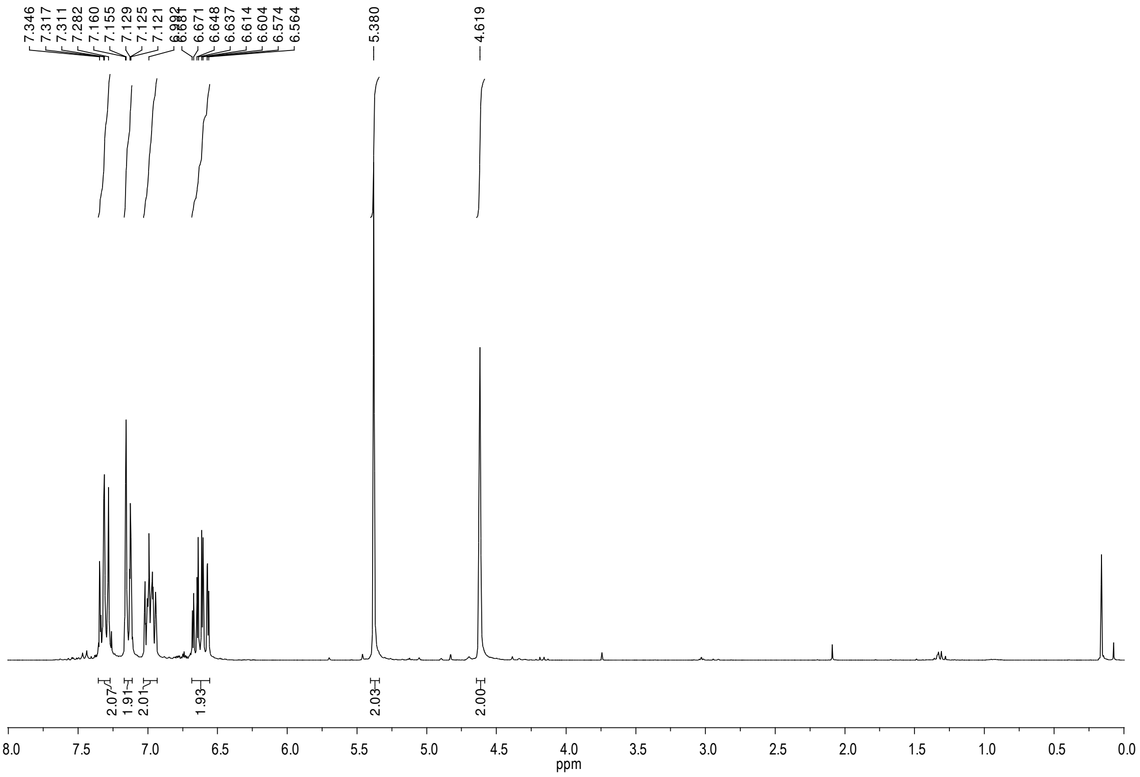

Figure S37. ^{1}H NMR (250 MHz) of **7** in $CDCl_3$.

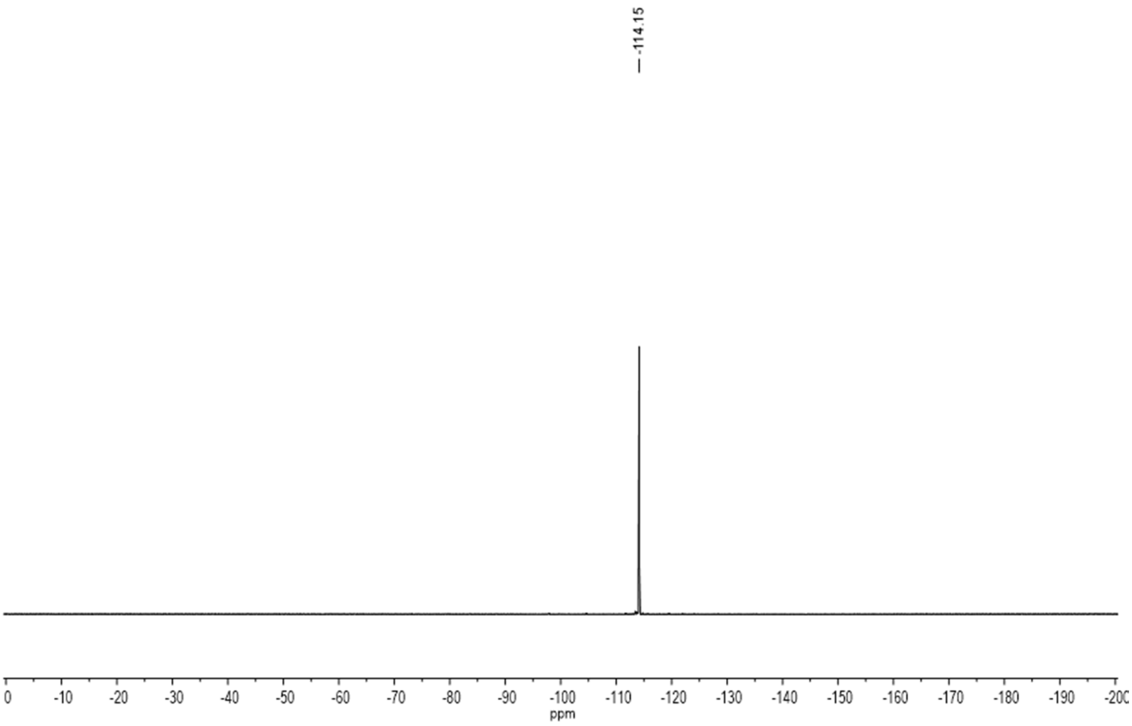

Figure S38. ^{19}F NMR (235.2 MHz) of **7** in $CDCl_3$.

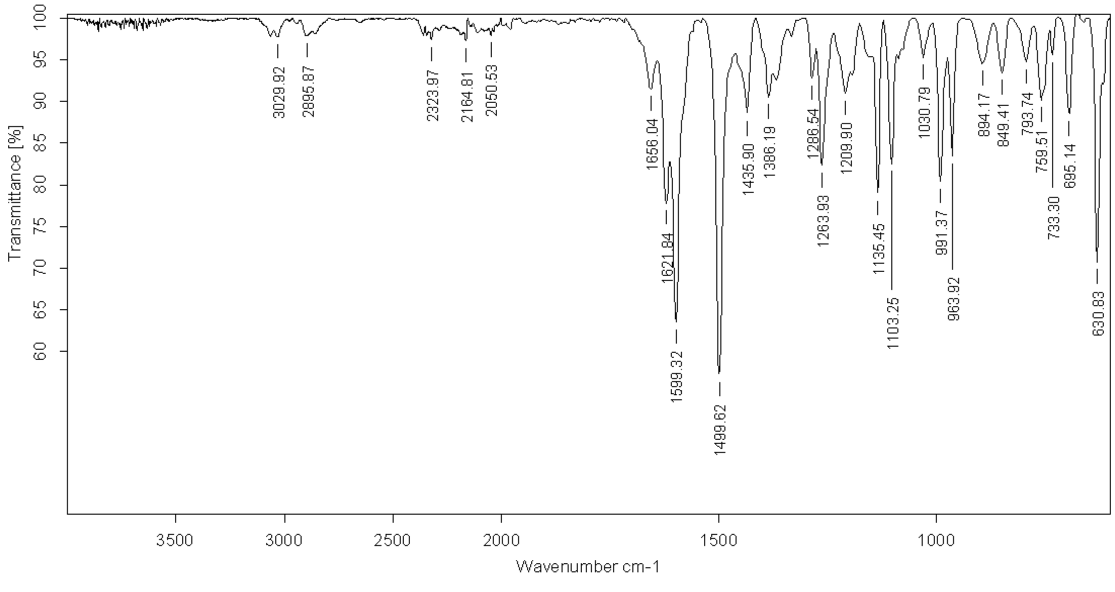

Figure S39. IR (ATR) υ (cm^{-1}) of **7**.

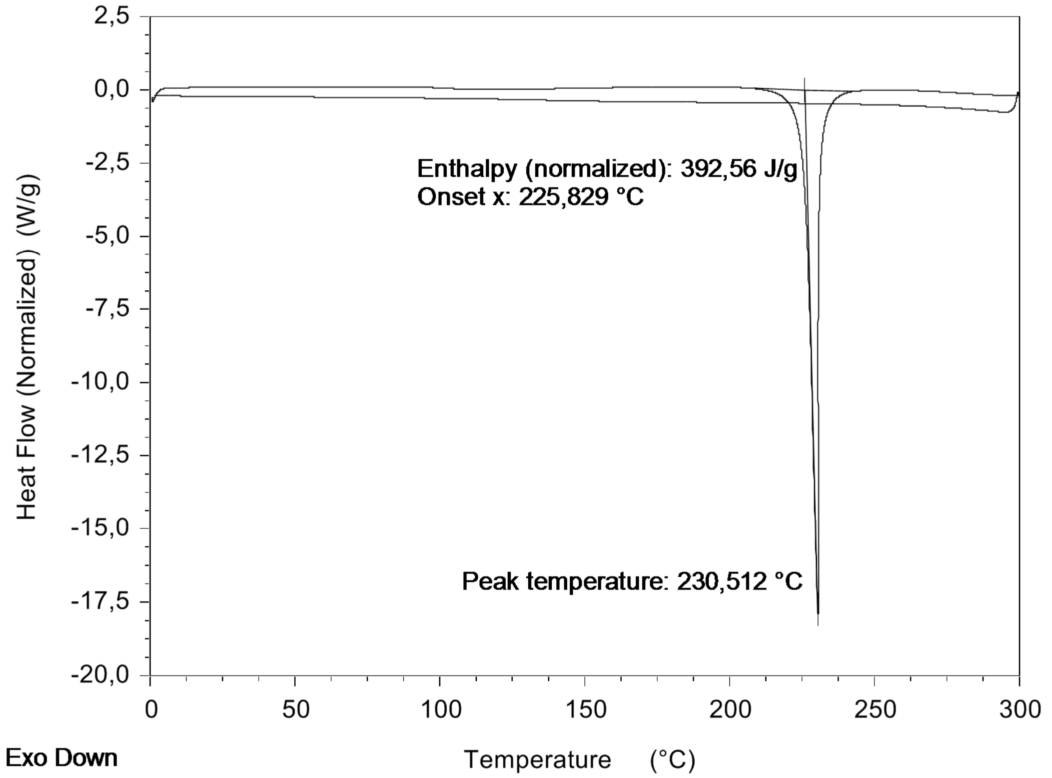

Figure S40. DSC thermogram of **7**.

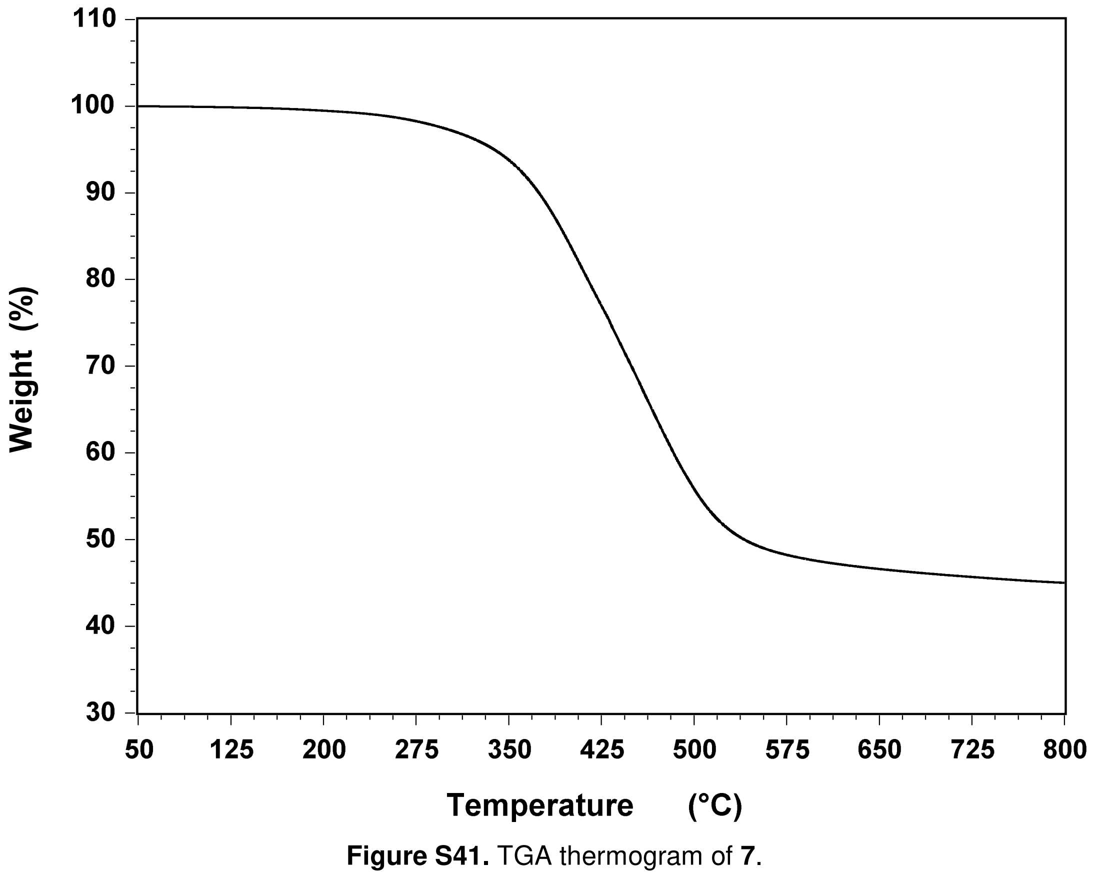

Figure S41. TGA thermogram of **7**.

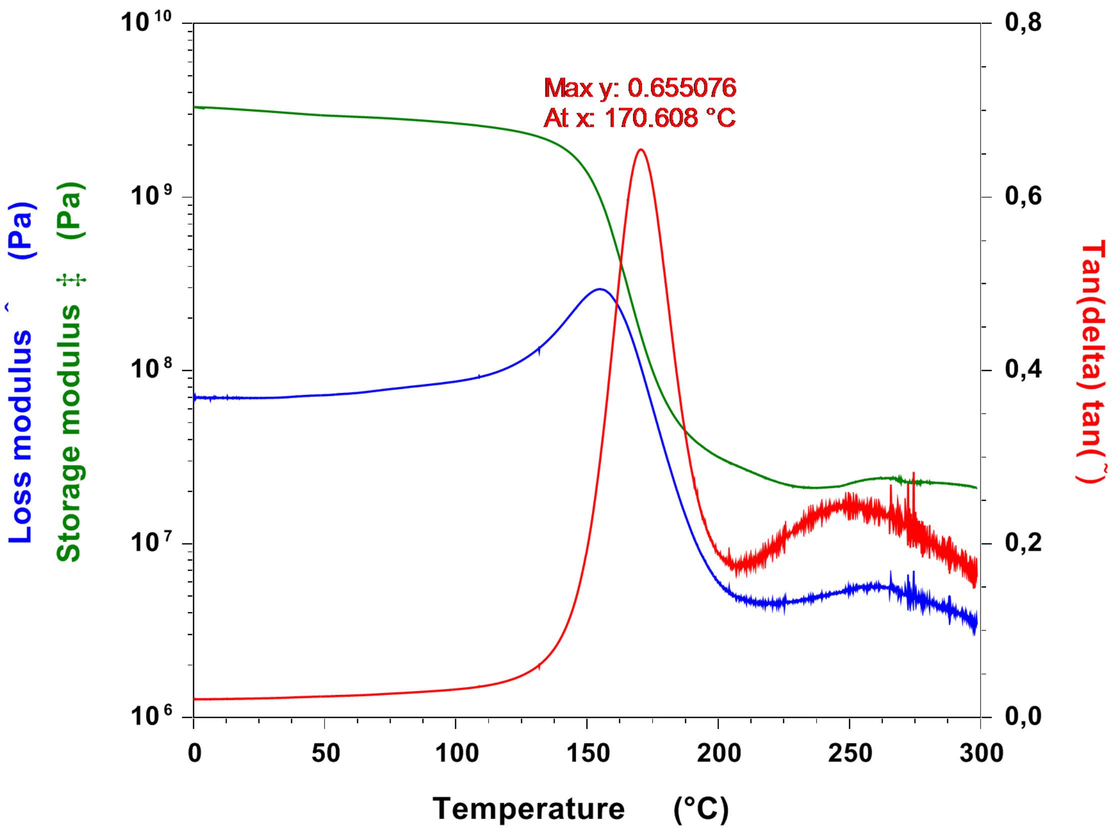

Figure S42. DMA thermogram of **7**.

S9. 7-Fluoro-3-phenyl-3,4-dihydro-2*H*-1,3-benzoxazine, 8 [2]

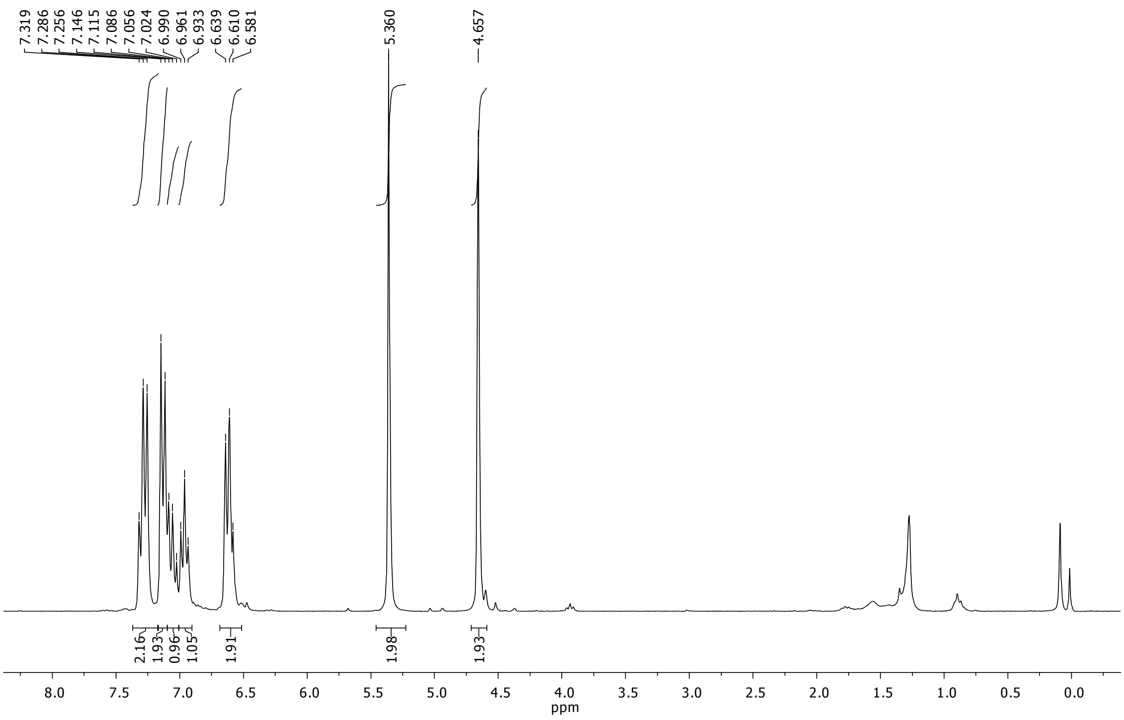

Figure S43. ^{1}H NMR (250 MHz) spectrum of **8** in $CDCl_3$.

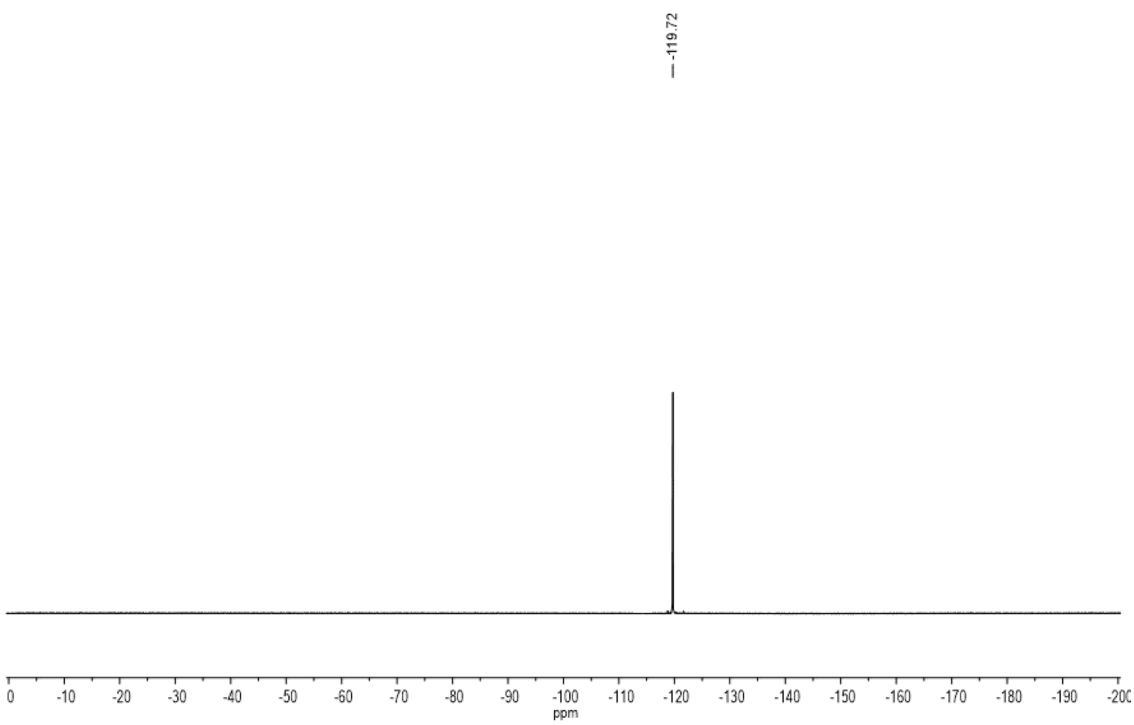

Figure S44. ^{19}F NMR (235.2 MHz) spectrum of **8** in $CDCl_3$.

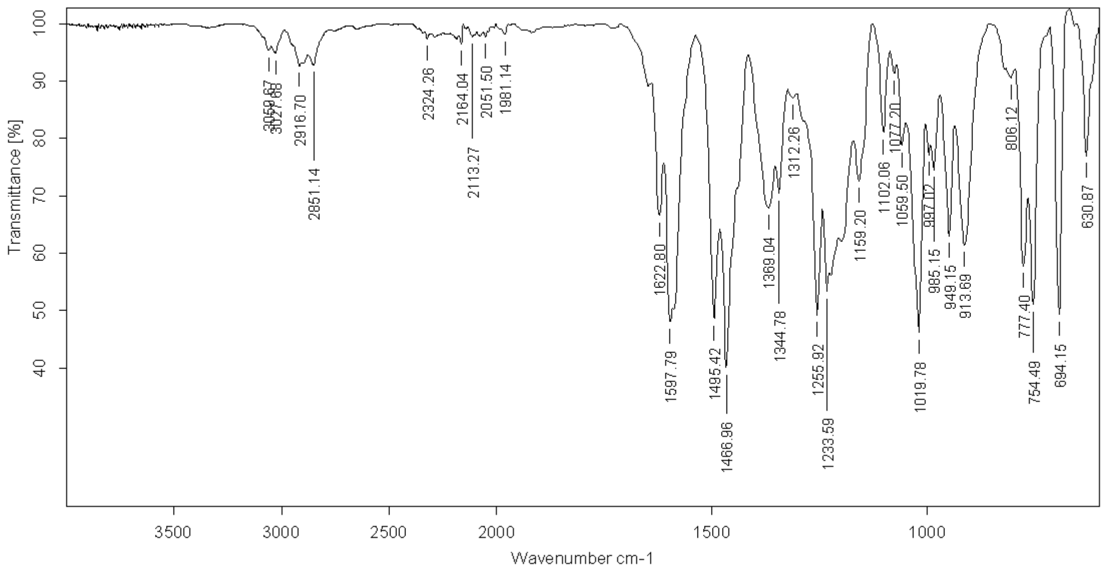

Figure S45. IR (ATR) υ (cm^{-1}) of **8**.

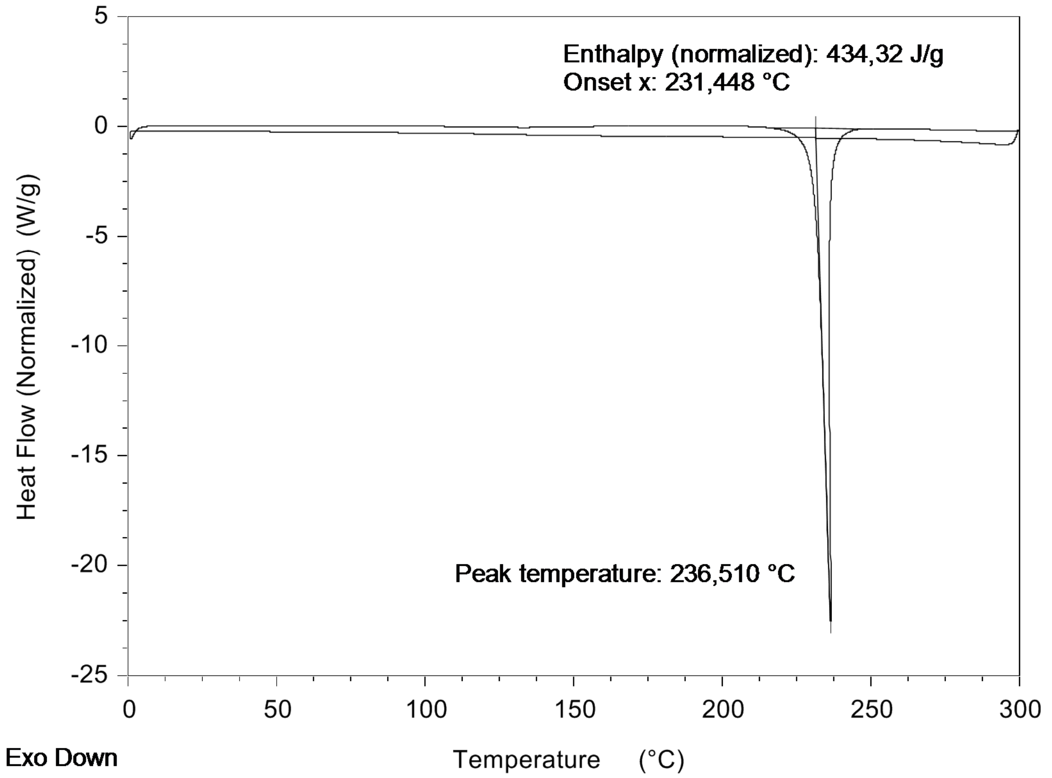

Figure S46. DSC thermogram of **8**.

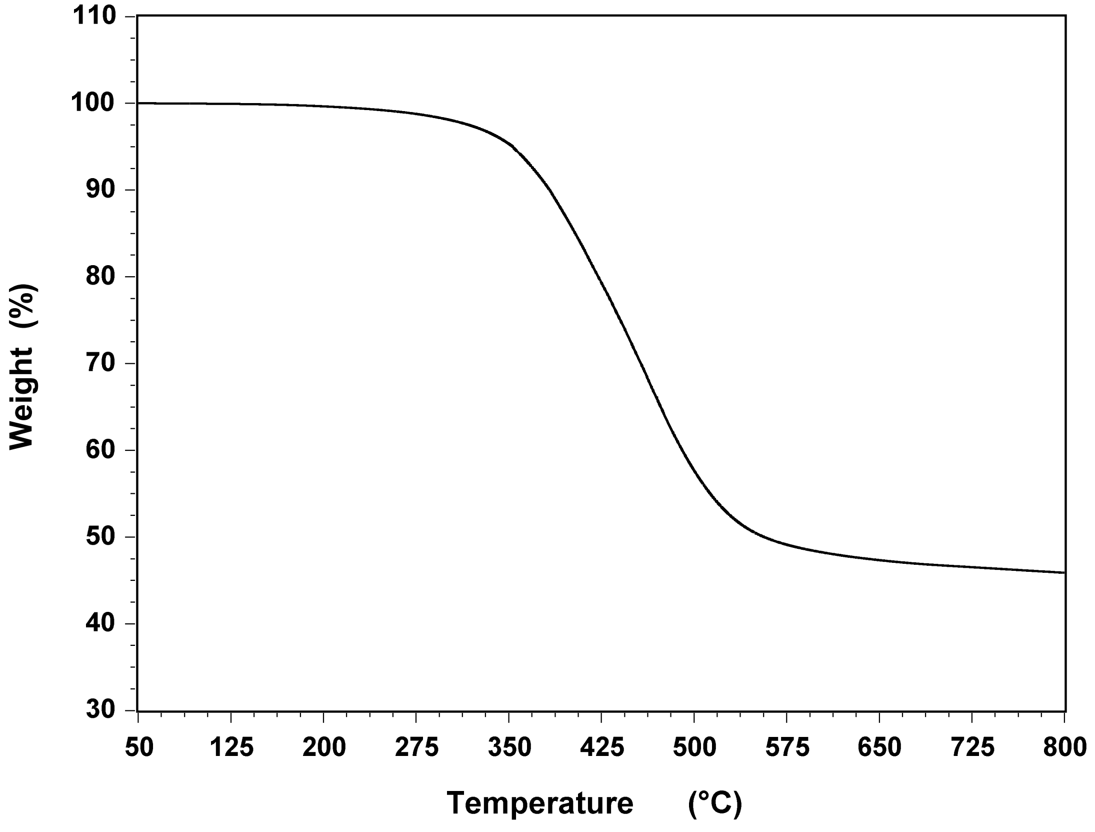

Figure S47. TGA thermogram of **8**.

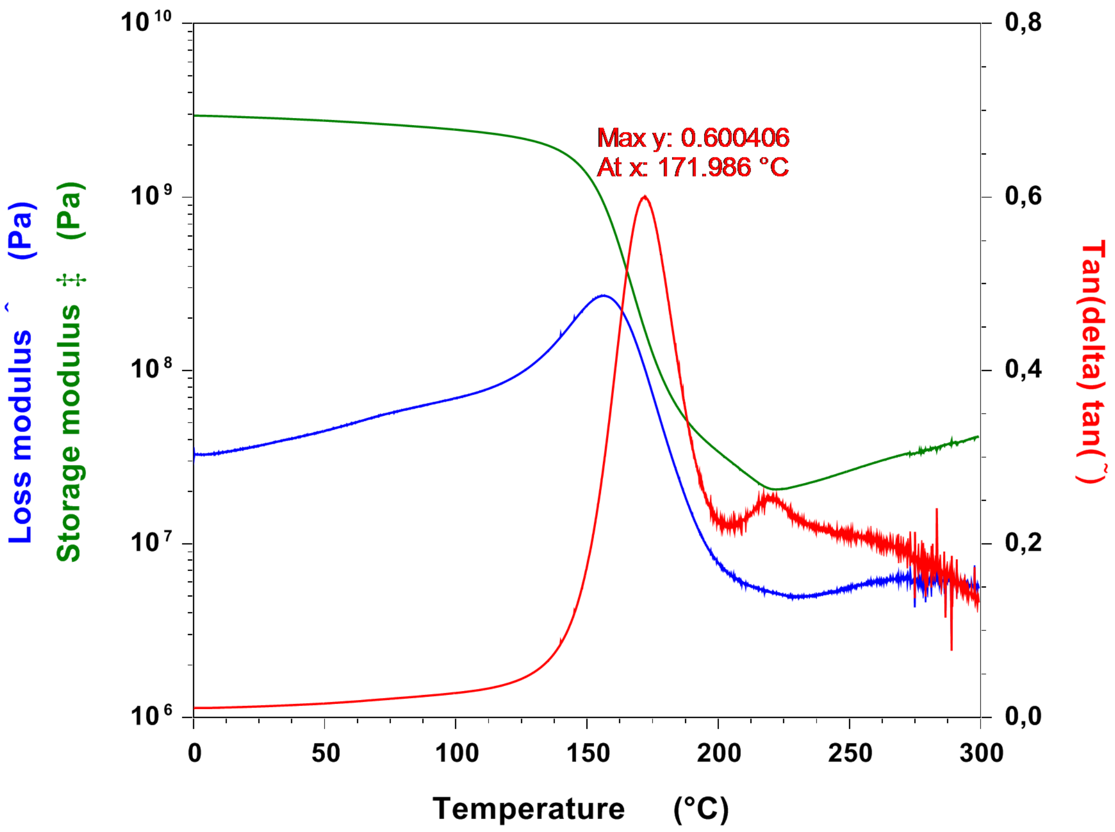

Figure S48. DMA thermogram of **8**.

S10. IR spectra of polymers obtained from benzoxazines 1-8 and BPA-a

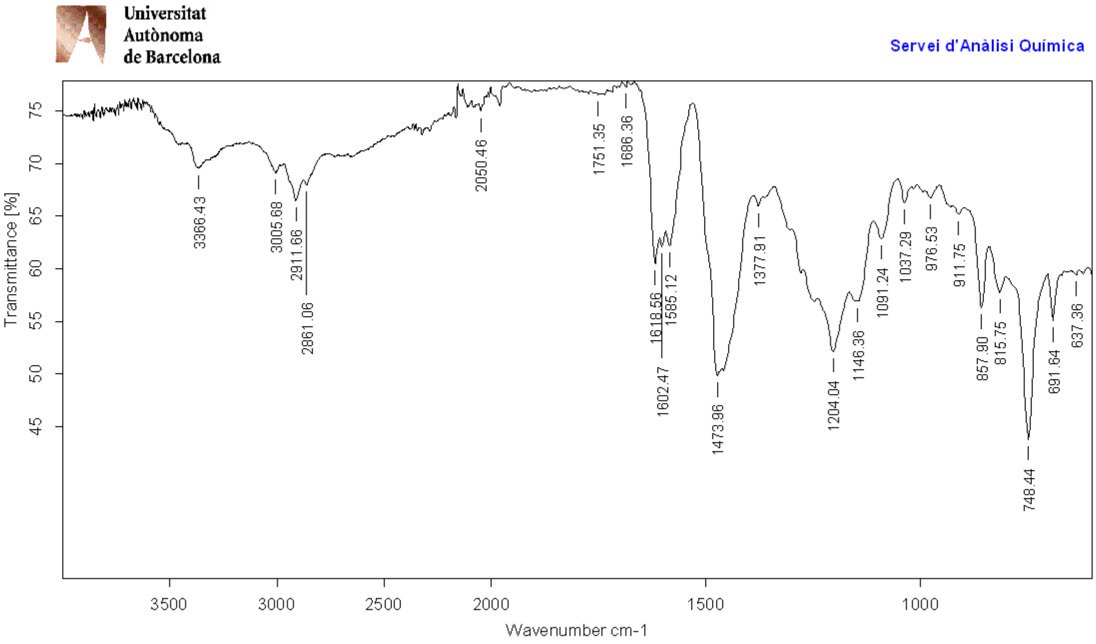

Figure S49. IR (ATR) υ (cm^{-1}) of polymer derived from **1**.

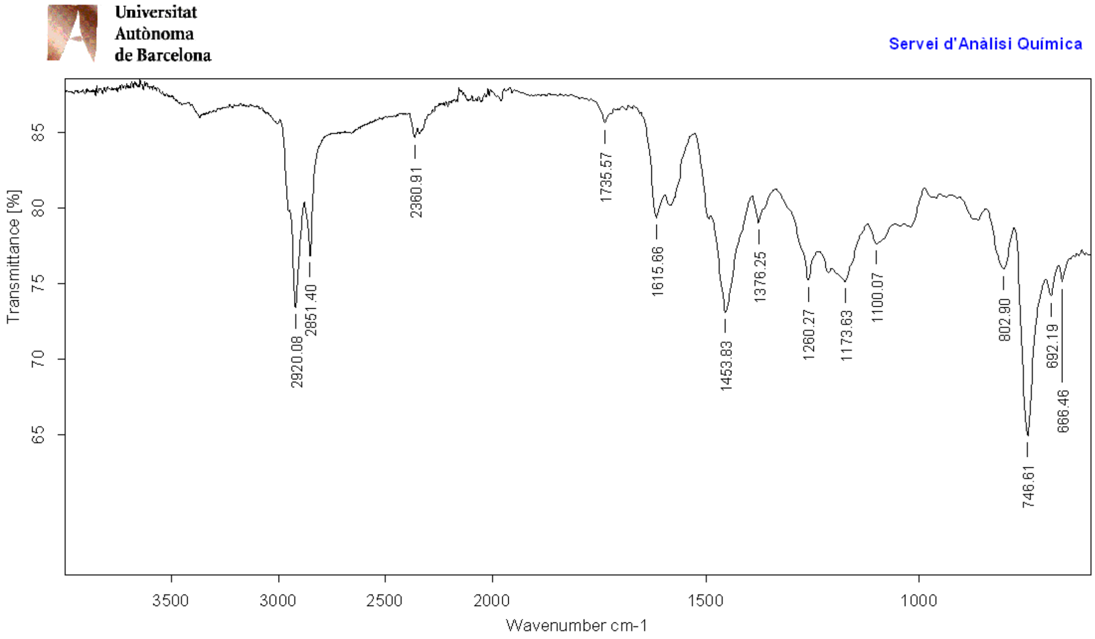

Figure S50. IR (ATR) υ (cm^{-1}) of polymer derived from **2**.

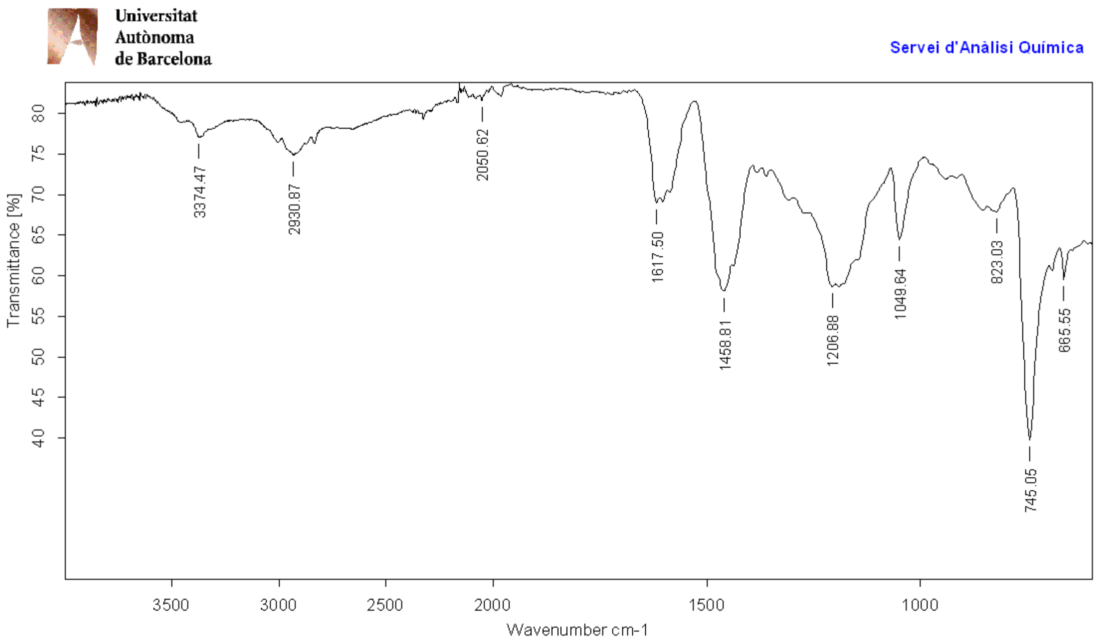

Figure S51. IR (ATR) υ (cm^{-1}) of polymer derived from **3**.

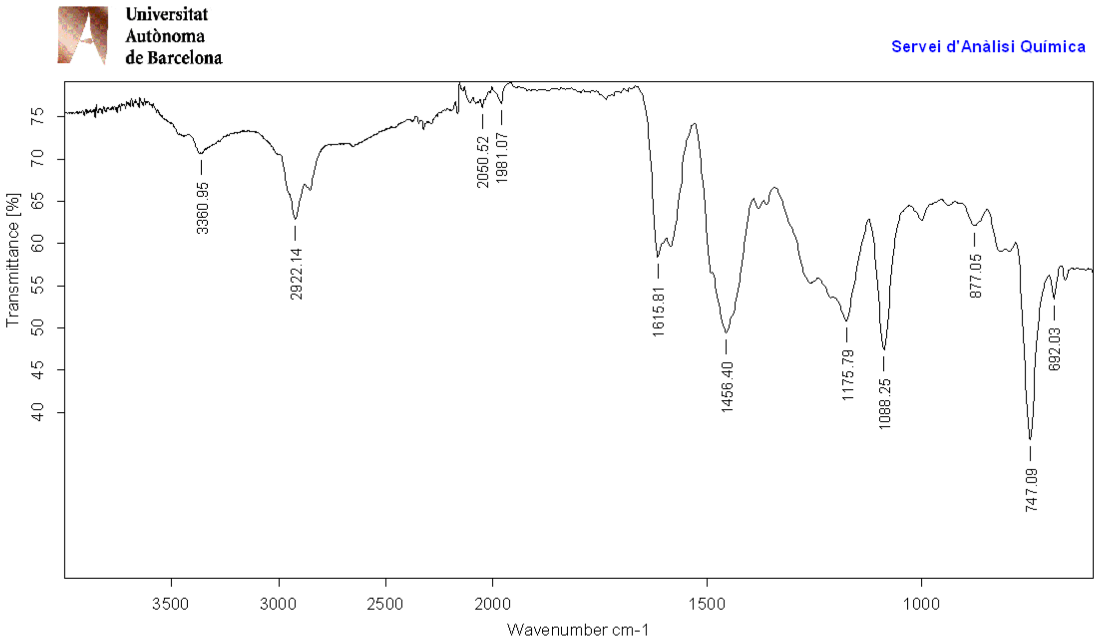

Figure S52. IR (ATR) υ (cm^{-1}) of polymer derived from **4**.

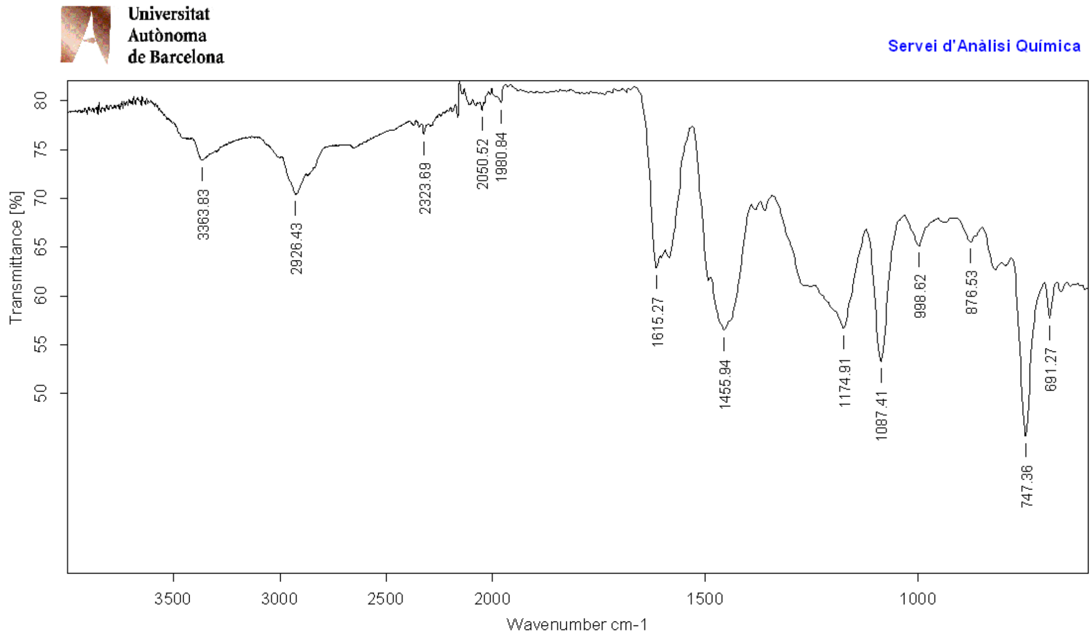

Figure S53. IR (ATR) υ (cm^{-1}) of polymer derived from **5**.

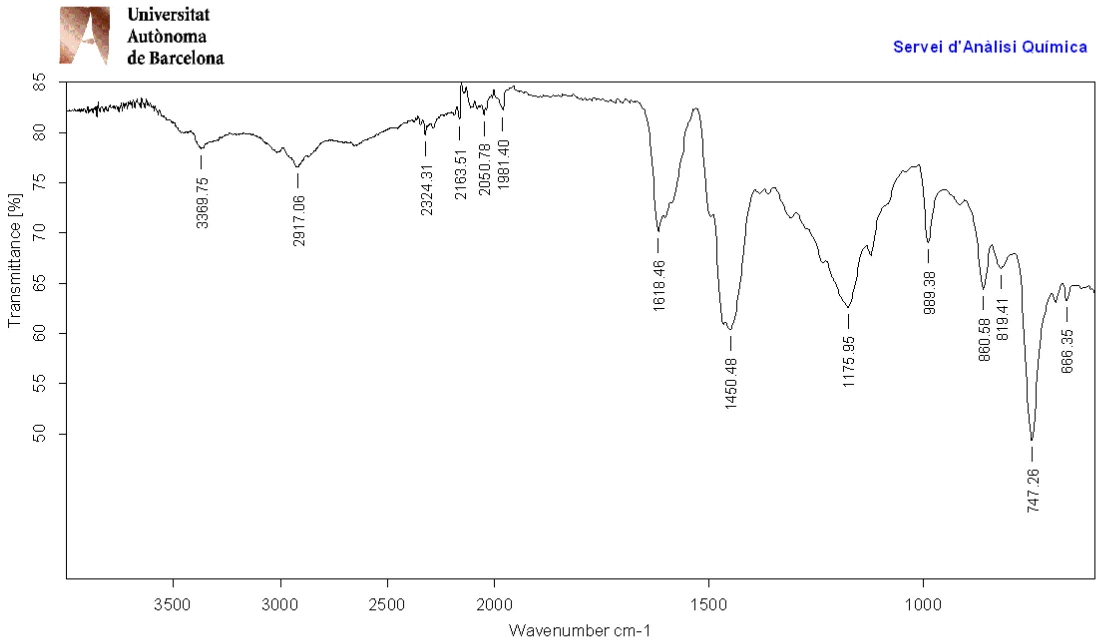

Figure S54. IR (ATR) υ (cm^{-1}) of polymer derived from **6**.

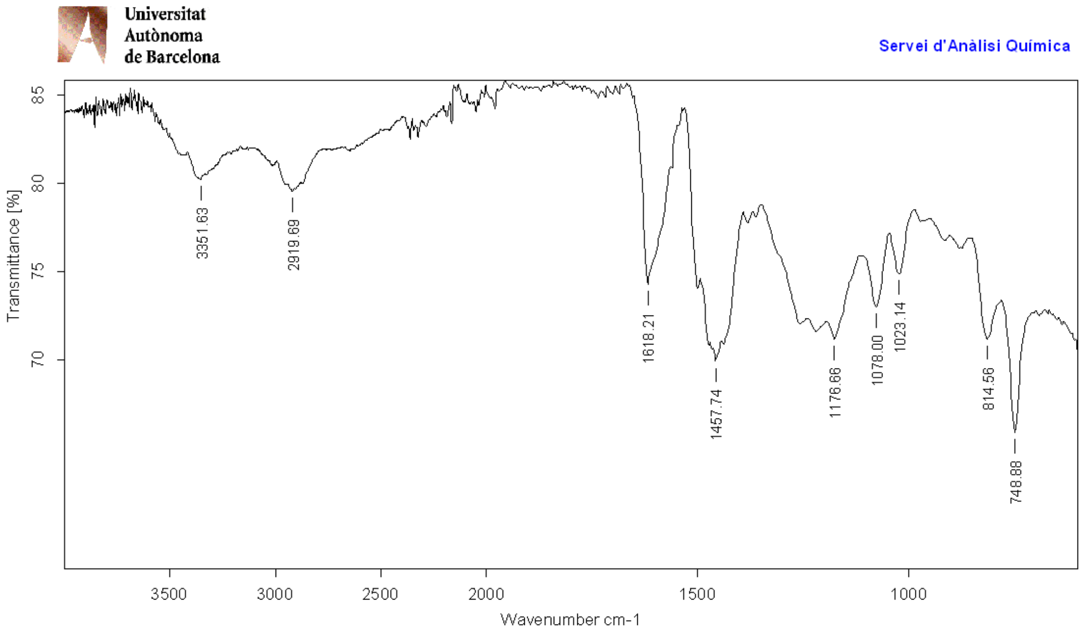

Figure S55. IR (ATR) υ (cm^{-1}) of polymer derived from **7**.

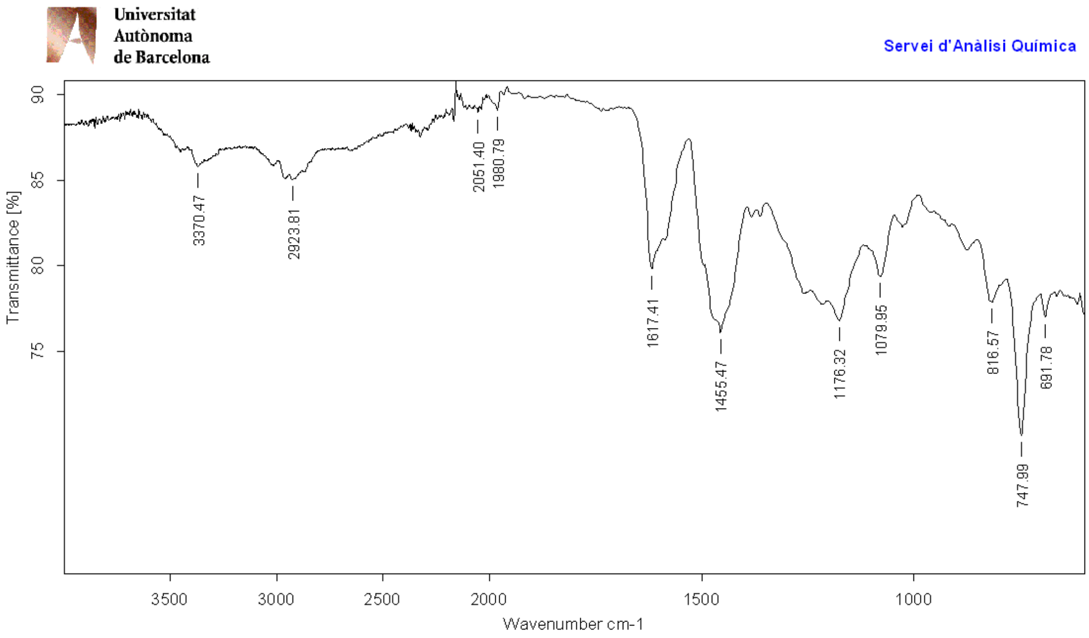

Figure S56. IR (ATR) υ (cm^{-1}) of polymer derived from **8**.

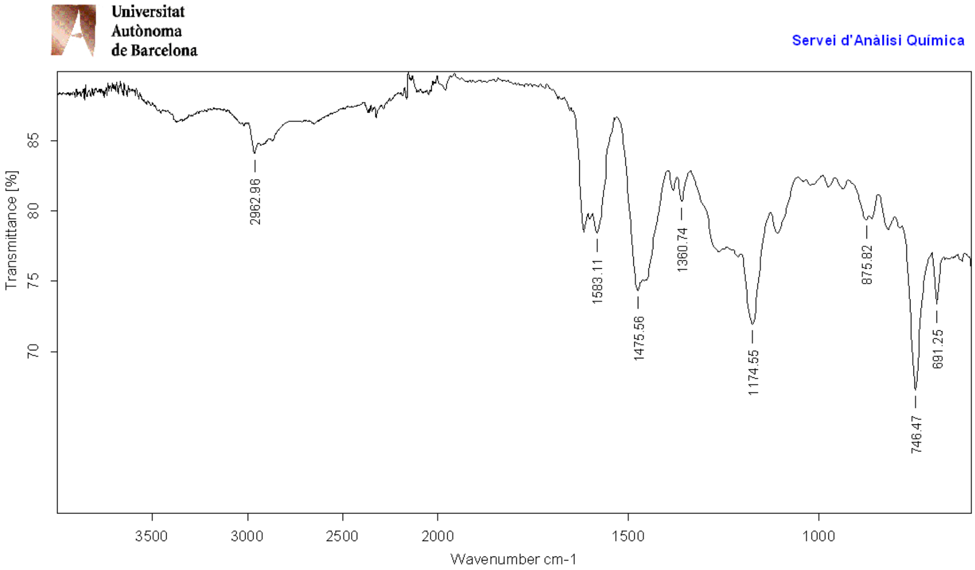

Figure S57. IR (ATR) υ (cm^{-1}) of polymer derived from **BPA-a**.

S11. ¹H-NMR spectra of soluble portion of polymers obtained from benzoxazines 1-8

After polymerization, obtained materials were grinded and DMSO-d_6 was added. The mixture was stirred for 1 h. The ¹H-NMR spectra of soluble parts were performed.

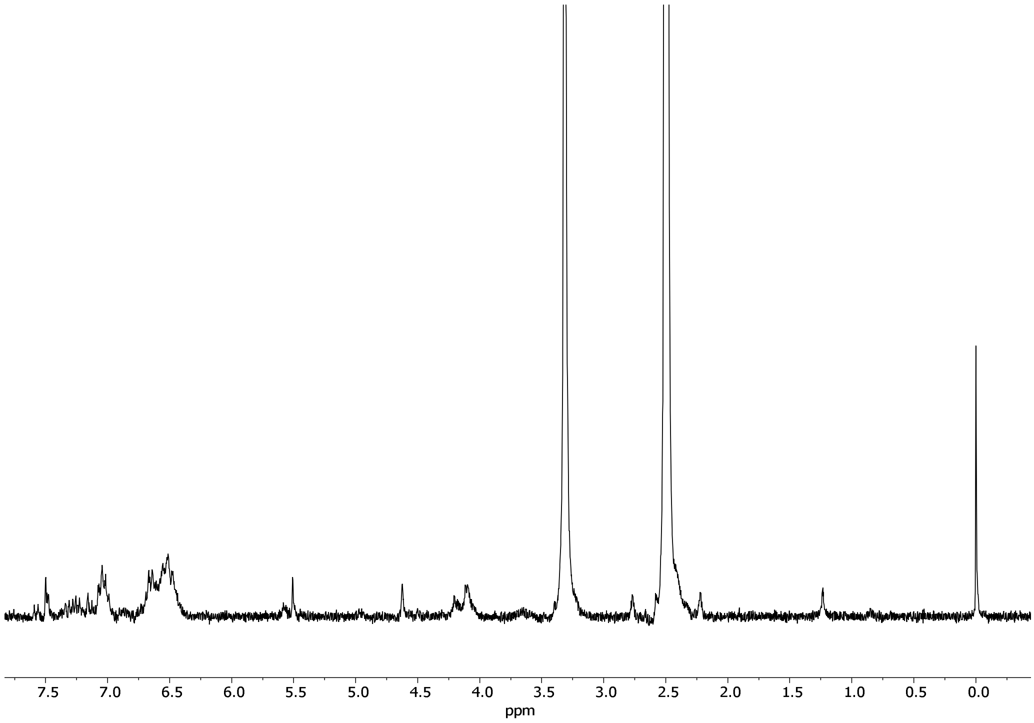

Figure S58. ¹H NMR (250 MHz) of **polymer** derived from **1** in DMSO-d_6.

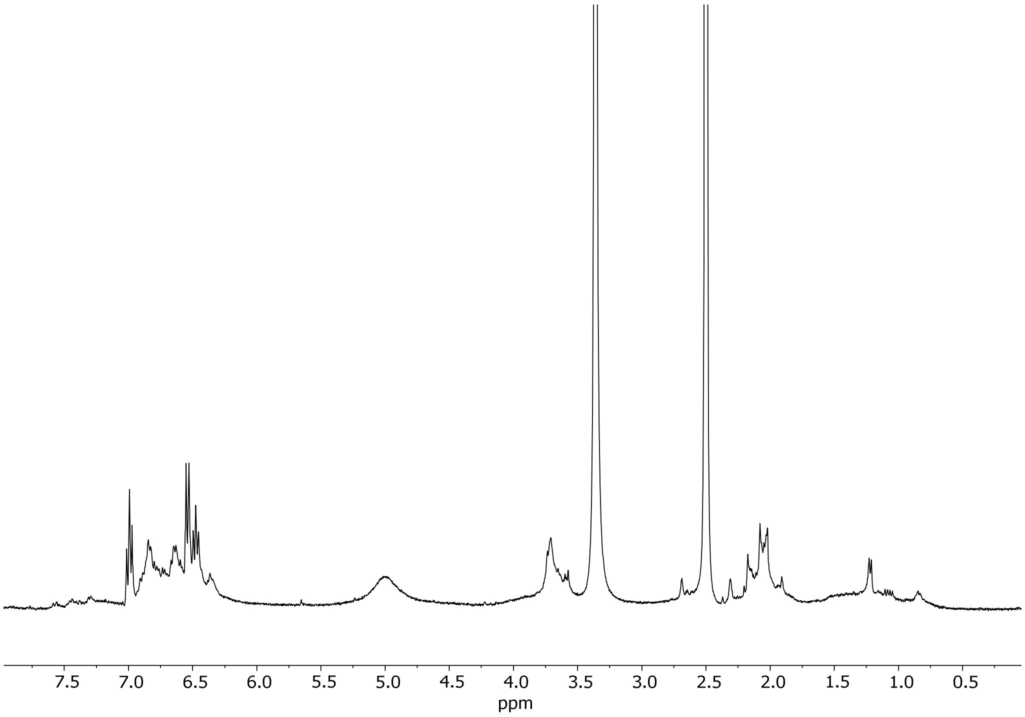

Figure S59. ¹H NMR (360 MHz) of **polymer** derived from **2** in DMSO-d_6.

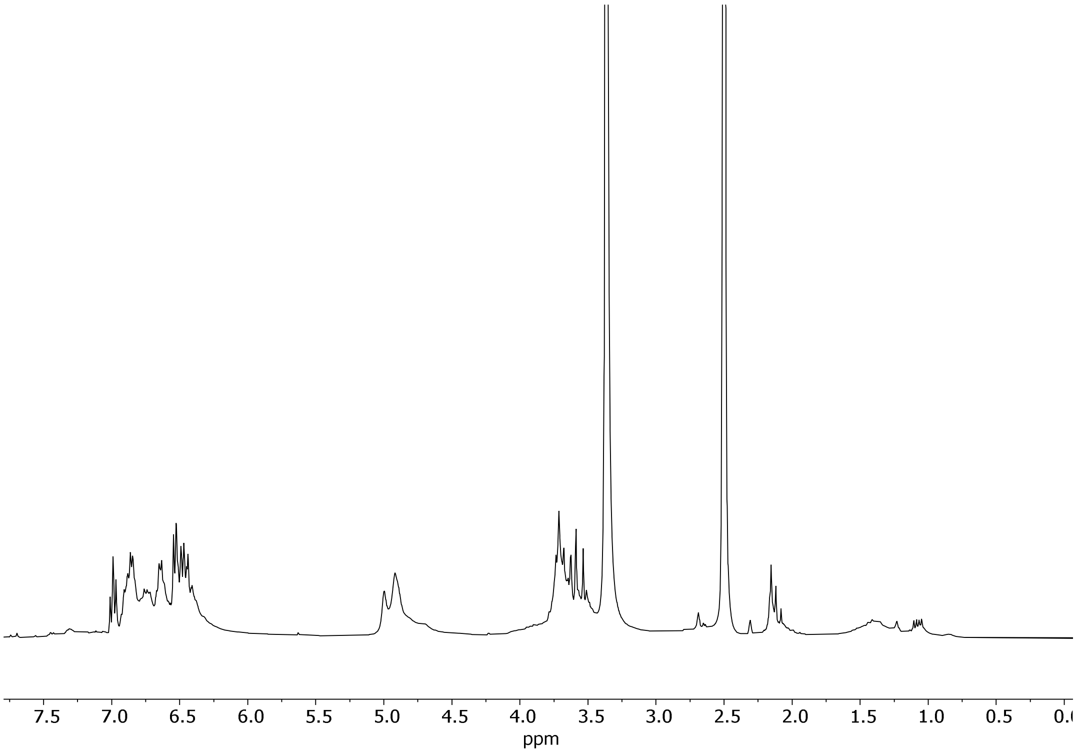

Figure S60. ^{1}H NMR (360 MHz) of **polymer** derived from **3** in DMSO-d_6.

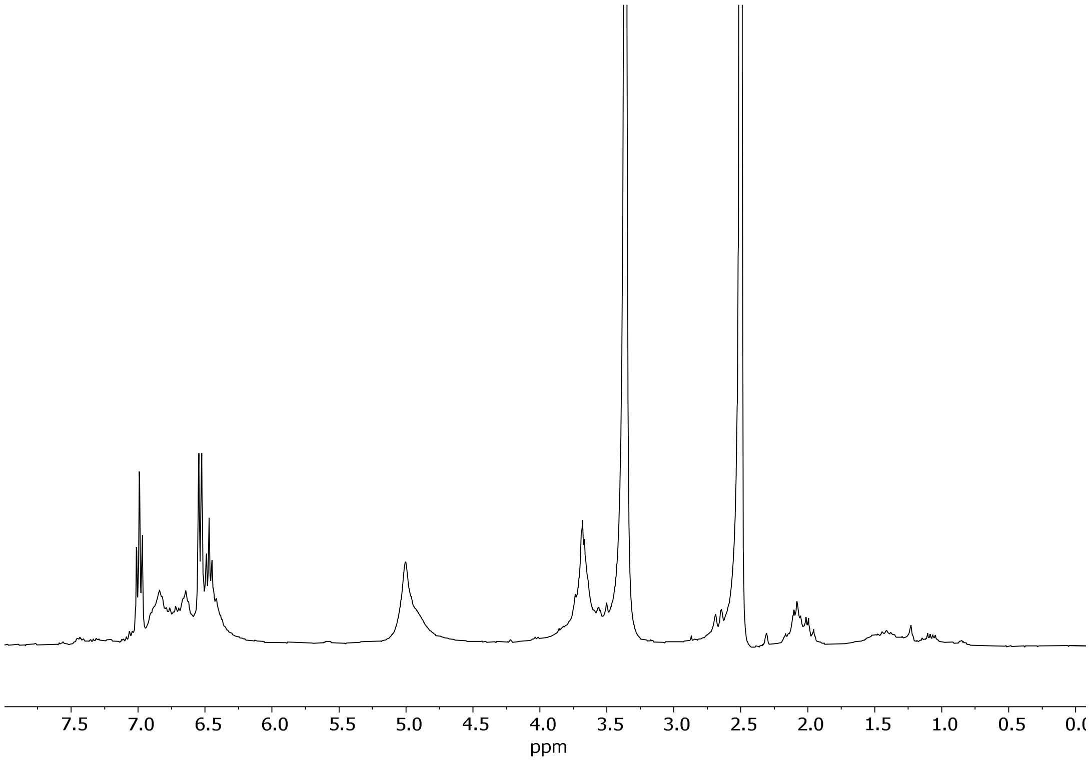

Figure S61. ^{1}H NMR (360 MHz) of **polymer** derived from **4** in DMSO-d_6.

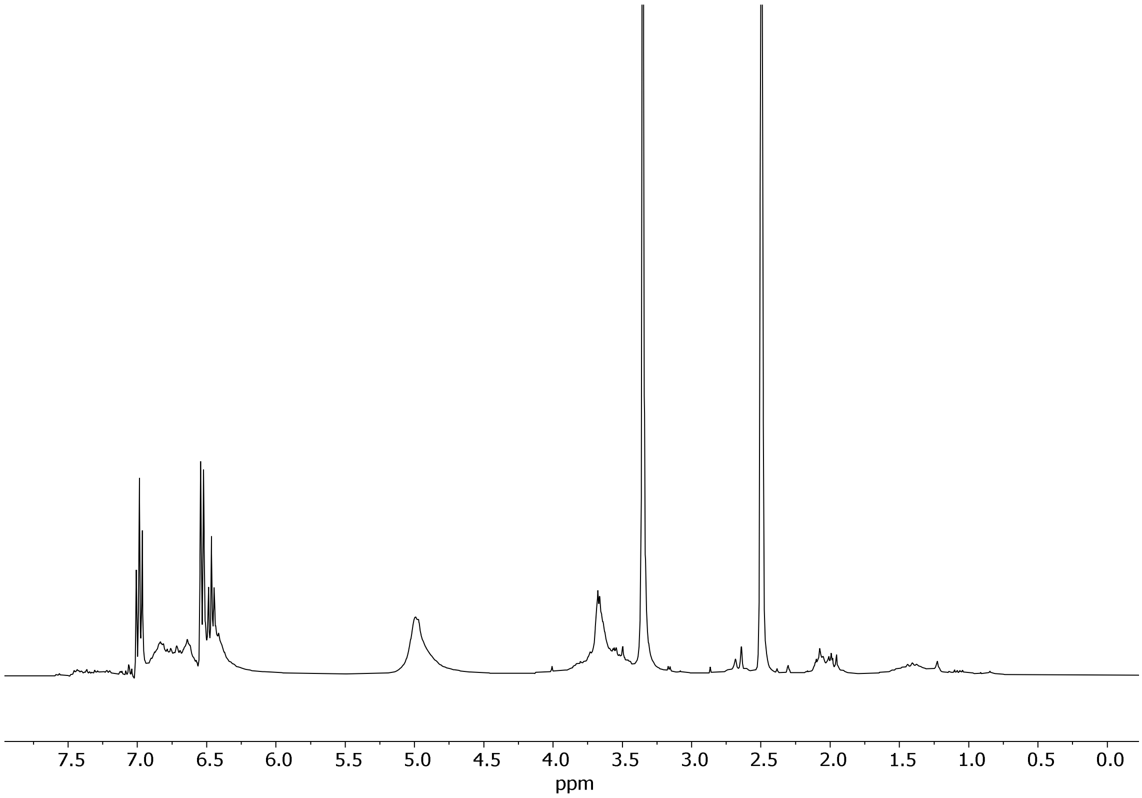

Figure S62. ^{1}H NMR (360 MHz) of **polymer** derived from **5** in DMSO-d_6.

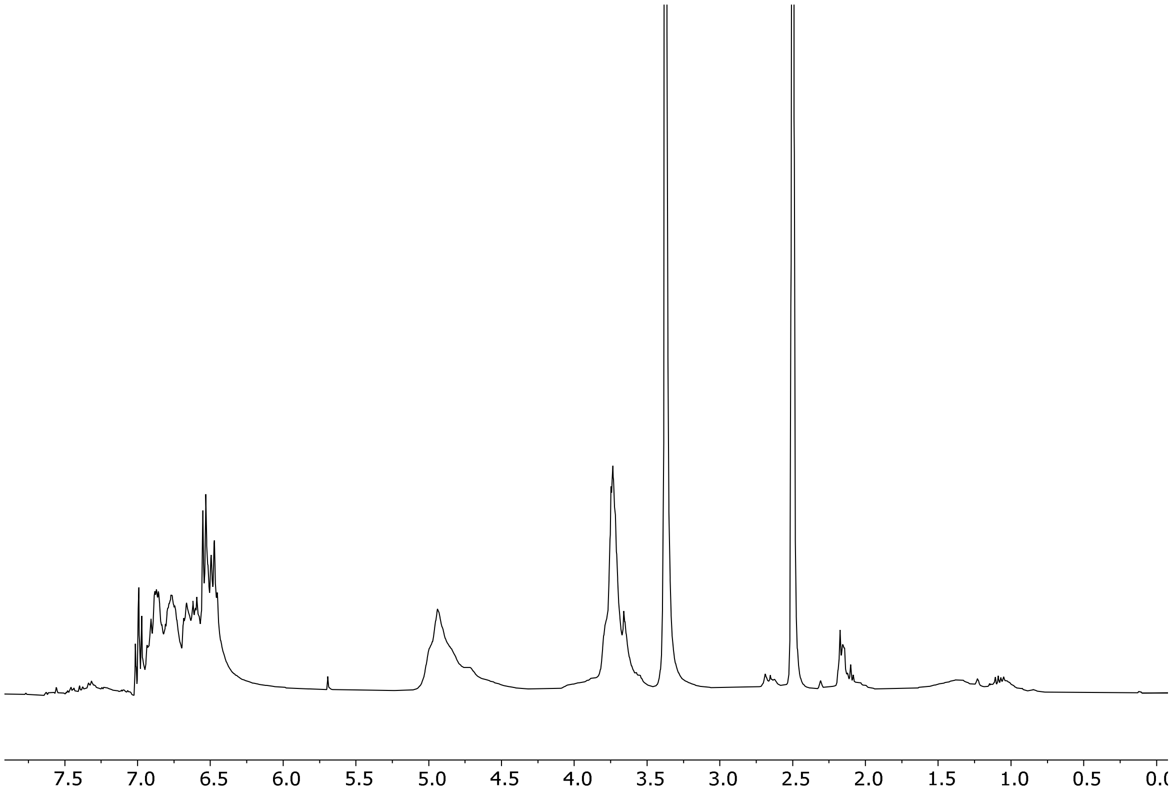

Figure S63. ^{1}H NMR (360 MHz) of **polymer** derived from **6** in DMSO-d_6.

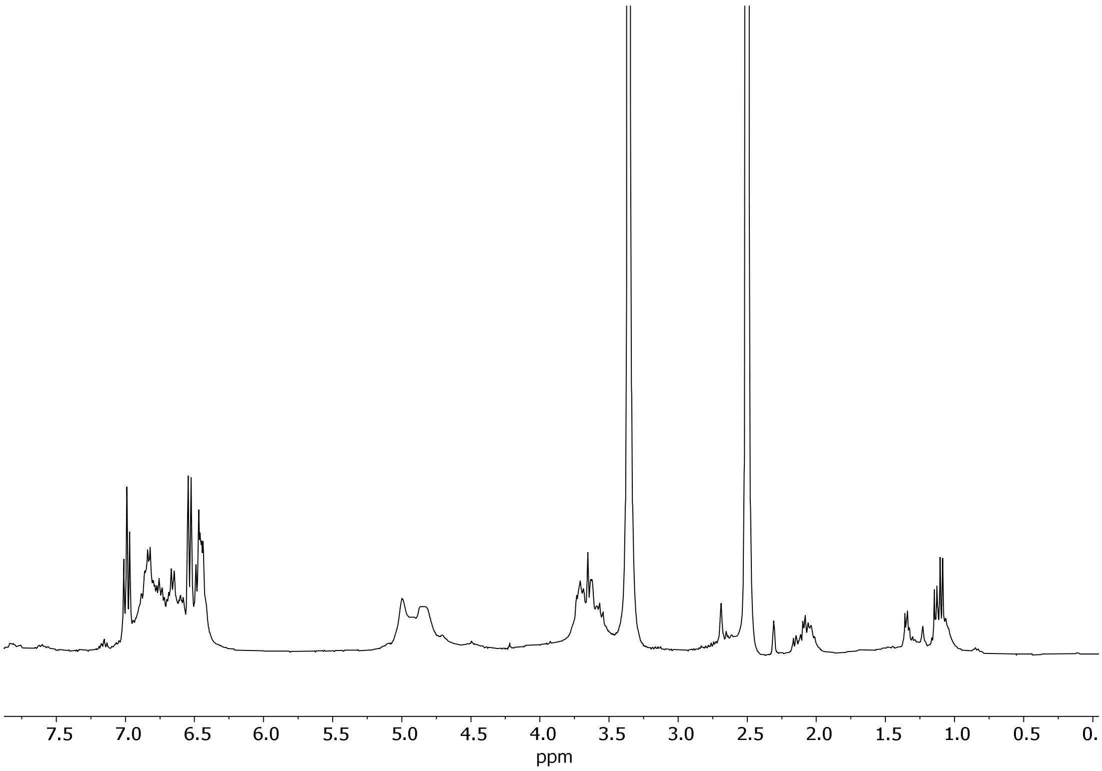

Figure S64. ^{1}H NMR (360 MHz) of **polymer** derived from **7** in DMSO-d_6.

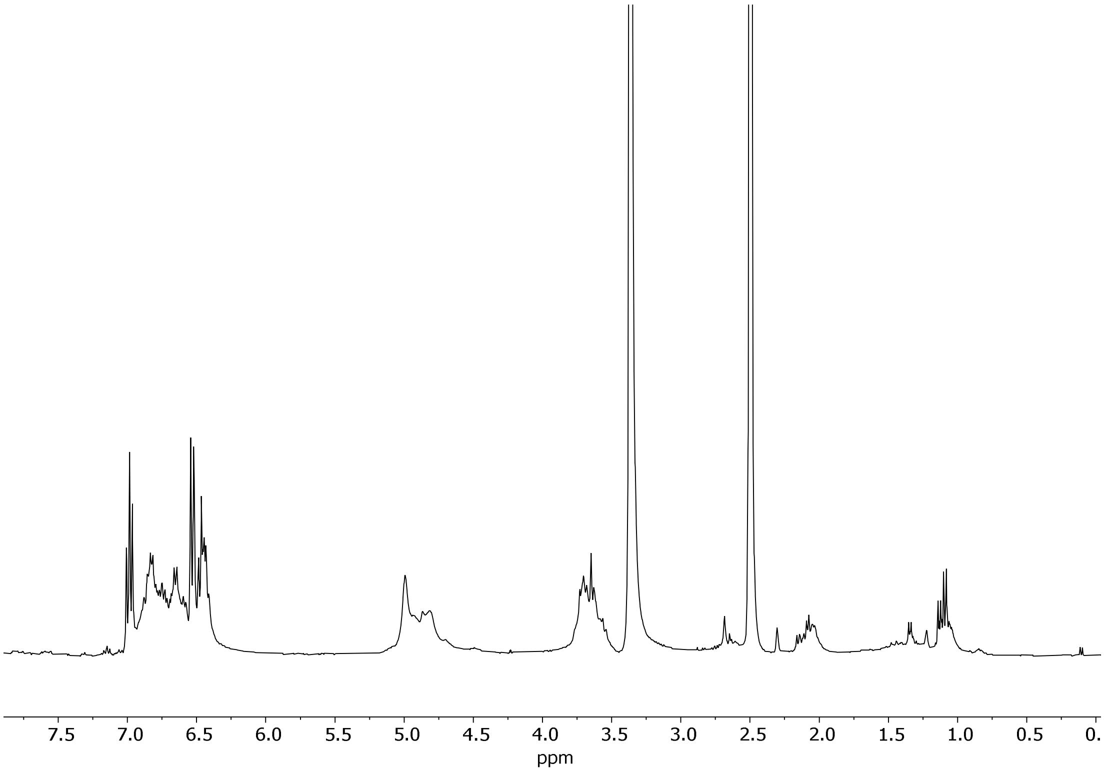

Figure S65. ^{1}H NMR (360 MHz) of **polymer** derived from **8** in DMSO-d_6.

Polymer derived from **BPA-a** was highly crosslinded and no fraction was soluble in DMSO-d_6. So, no remaining monomer was observed by ^{1}H NMR after washing the material with this solvent.

S12. References:

[1] Moloney, G. P.; Craik, D.J.; Iskander, M.N. Qualitative analysis of the oxazine ring of various benzoxazine and pyridooxazine derivatives with proton nuclear magnetic resonance spectroscopy. *J. Pharm. Sci.* **1992**, *81*, 692-697. DOI: https://doi.org/10.1002/jps.2600810721

[2] Martos, A.; Sebastián, R.M.; Marquet, J. Studies on the ring-opening polymerization of benzoxazines: Understanding the effect of the substituents. *Eur. Polym. J.* **2018**, *108*, 20-27. DOI: https://doi.org/10.1016/j.eurpolymj.2018.08.025

[3] Takeichi, T.; Nakamura, K.; Agag, T.; Muto, H. Synthesis of cresol-based benzoxazine monomers containing allyl groups and the properties of the polymers therefrom. *Desig. Monom. Polym.* **2004**, *7*, 727-740. DOI: https://doi.org/10.1163/1568555042474121

[4] Andreu, R.; Reina, J.A.; Ronda, J.C. Studies on the thermal polymerization of substituted benzoxazine monomers: Electronic effects. *J. Polym. Sci. A*, **2008**, *46*, 3353-3366. DOI: https://doi.org/10.1002/pola.22677
